# Lonidamine-1,3,4-oxadiazole derivatives with antiproliferative effects on HCT116 colon cancer cell lines: biological evaluation, ADMET, and computational studies

**DOI:** 10.1039/d5ra07852k

**Published:** 2026-03-11

**Authors:** Raveendra Madhukar Bhat, A. N. Priyadarshini, M. S. Sudhanva, Gangadhar V. Muddapur, Kawthar Alhussieni, Raman Kumar K, Rangappa S. Keri

**Affiliations:** a Centre for Nano and Material Sciences, Jain (Deemed-to-be University) Jain Global Campus, Jakkasandra Post, Kanakapura Road, Ramanagara District Bangalore Karnataka 562112 India sk.rangappa@jainuniversity.ac.in keriphd@gmail.com +918027577199 +919620667075; b Aurigene Pharmaceutical Services Bangalore Karnataka India; c Adichunchanagiri Institute for Molecular Medicine, Adichunchanagiri Institute of Medical Sciences, Adichunchanagiri University BG Nagara-571448 Karnataka India; d Department of Physics, KLE Technological University Hubballi-580031 Karnataka India; e Department of Pharmaceutical Chemistry, Faculty of Pharmacy, Universiti Malaya Kuala Lumpur 50603 Malaysia

## Abstract

In recent years, cancer has emerged as a significant challenge for healthcare systems, posing challenges to researchers to develop new treatments. Among various cancers, colorectal cancer is a major cause of death worldwide. To develop novel compounds that strongly inhibit colon cancer cells, lonidamine-1,3,4-oxadiazole derivatives 7(a–h) were designed and synthesized. The prepared compounds were characterized by various spectral techniques, including NMR (^1^H and ^13^C), mass spectra, and HPLC. Cytotoxicity tests conducted on a colorectal cancer cell line indicated that compound (7d) demonstrated significant antiproliferative effects, achieving the lowest IC_50_ value of 12.91 ± 1.58 µM, thereby making it the most effective among the compounds tested. This compound induces apoptosis, as evidenced by Hoechst/PI double staining, and mitigates cell migration, demonstrating its antiproliferative and antimigratory capabilities. Molecular docking, dynamics simulations, and DFT studies helped clarify the structure–activity relationship (SAR) and mechanisms of action. ADME and toxicity predictions also supported its drug-like properties. SAR analysis identified key substituents influencing activity, guiding further optimization. Overall, compound (7d) appears to be a promising candidate for colon cancer therapy, though additional studies are necessary to assess its clinical potential. These findings could be used for designing novel cancer therapeutic or preventive LND-derived agents.

## Abstract 2

Herein, we have employed a structure-based approach, designed and synthesized conjugates of lonidamine-1,3,4-oxadiazole, and evaluated its cytotoxicity profile on a colorectal cancer cell line.

## Graphical abstract

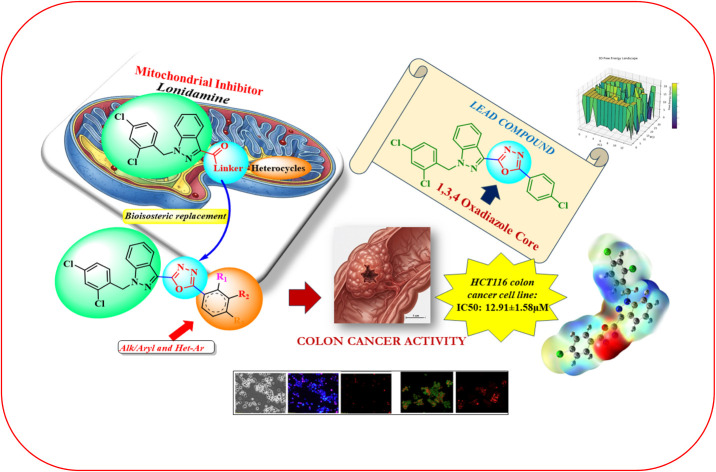

## 1 Introduction

Colon cancer, also known as colorectal cancer (CRC), is one of the most common types of cancer that originates in the colon of the digestive tract. It contributes to significant morbidity and mortality.^1^ CRC is the third most often diagnosed malignancy and the second largest cause of cancer-related mortality.^2,3^ In 2020, the global figures indicated 1.9 million new cases and fatalities, an increase from 1.8 million new cases and 861 000 deaths in 2018.^4,5^ Additionally, it is projected that 107 320 more cases will be identified by 2025, and approximately 53 000 individuals are expected to die from colon cancer in 2024.^6^ The worldwide burden of CRC is projected to rise by 60% by 2030, leading to an estimated 2.2 million new cases and 1.1 million deaths.^7^

Colon cancer frequently begins with the formation of tiny, benign clusters of cells known as polyps in the colon, which can eventually lead to cancer.^8^ Age, smoking, inflammatory bowel disease, inheritance, and dietary habits all have a substantial impact on the development of colon cancer.^9^ Colon cancer displays no visible early signs, and it is typically identified in its middle or late stages. Surgery, chemotherapy, immunotherapy, radiation, and targeted therapies are the most common treatment methods for colon cancer today.^10^ Among these, chemotherapy plays a crucial role throughout colon cancer treatment, especially for advanced cases.^11^ FDA-approved frontline chemotherapeutic drugs such as folinic acid, methotrexate, 5-fluorouracil, bevacizumab (avastin), oxaliplatin, and celecoxib (celebrex) are used to treat CRC.^12,13^ However, these medications have limitations such as adverse side effects, short half-lives, limited tumor selectivity, absorption in the upper GI tract, and drug resistance, which can lead to treatment failure and disease progression.^14,15^ As a result, there is a pressing need to find innovative treatment options with considerable activity and efficacy against CRC.

**Fig. 1:**
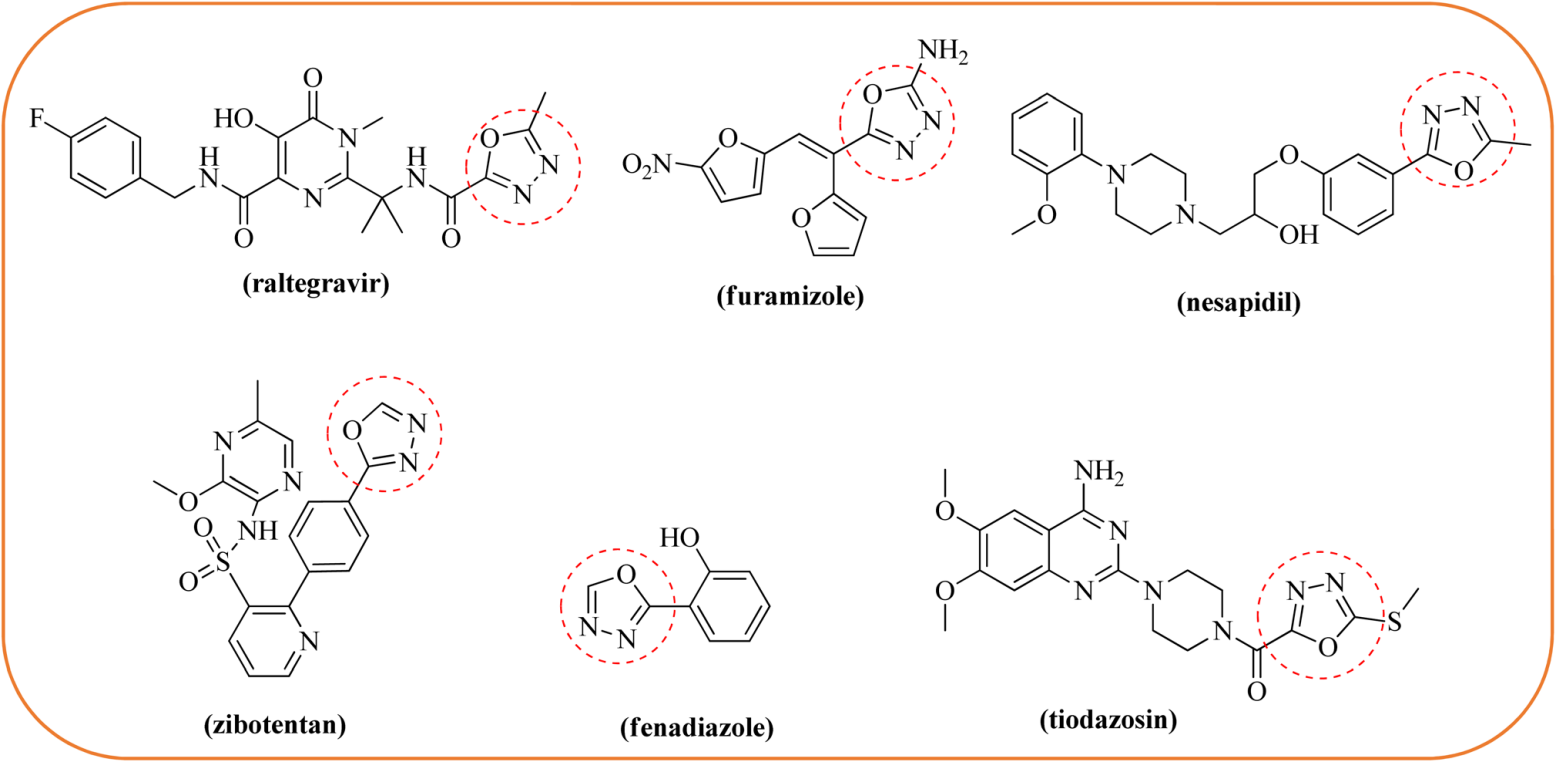
Commercially available drugs with a 1,3,4-oxadiazole moiety.

Oxadiazoles are novel heterocyclic compounds with substantial applications in synthetic and medicinal chemistry.^16^ Oxadiazoles have four regioisomers: 1,2,3-, 1,2,4-, 1,2,5-, and 1,3,4.^16^ Among these region isomers, 1,3,4-oxadiazoles play an important role in medicinal chemistry and display a wide range of biological actions,^17^ such as anti-tuberculosis,^18^ anticancer,^19^ antimalarial,^20^ antibacterial,^21^ anticonvulsant, antidiabetic,^22^ and anti-inflammatory.^23^ In addition, various medications having a 1,3,4-oxadiazole core are available in the market, including raltegravir (antiretroviral drug), furamizole (antibiotic drug), nesapidil (antiarrhythmic drug), zibotentan (anticancer agent), fenadiazole (hypnotic drug), and tiodazosin (adrenergic receptor antagonist)^24^ (Fig. 1). In contrast, lonidamine (LND) is an indazole derivative (1-[(2,4-dichlorophenyl) methyl]-1*H*-indazole-3-carboxylic acid) that was formerly employed as an anti-spermatogenic drug but is now known for its anti-tumor and pro-apoptotic properties.^25,26^ This drug has undergone pre-clinical testing and has been examined in Phase II and Phase III trials targeting lung cancer, and it has been demonstrated to be safe but with limited efficacy.^27^ When combined with other pharmacophores or chemotherapeutics, LND demonstrated increased anticancer effects in the treatment of breast, prostate, and ovarian tumors.^28,29^

With this in mind, and to minimize LND's side effects while improving its pharmacological profile and therapeutic efficacy, we've continued our efforts to develop new anticancer agents.^30,31^ We developed a rational strategy to replace LND's carboxyl functional group with a 1,3,4-oxadiazole bioisostere. This modification is anticipated to enhance the compound's pharmacokinetic and pharmacodynamic properties while preserving its mechanism of action. We also evaluated the *in vitro* anticancer activity against HCT116 colon cancer cell lines and conducted *in silico* analyses, including molecular docking simulations, density functional theory (DFT) assessments, ADME (absorption, distribution, metabolism, and excretion) evaluations, and toxicity studies. This analysis provided insights into how the compounds interact with the active site. We discussed the findings regarding structure–activity relationships and identified the most promising compounds as potential treatment options.

## 2 Design strategy

**Fig. 2:**
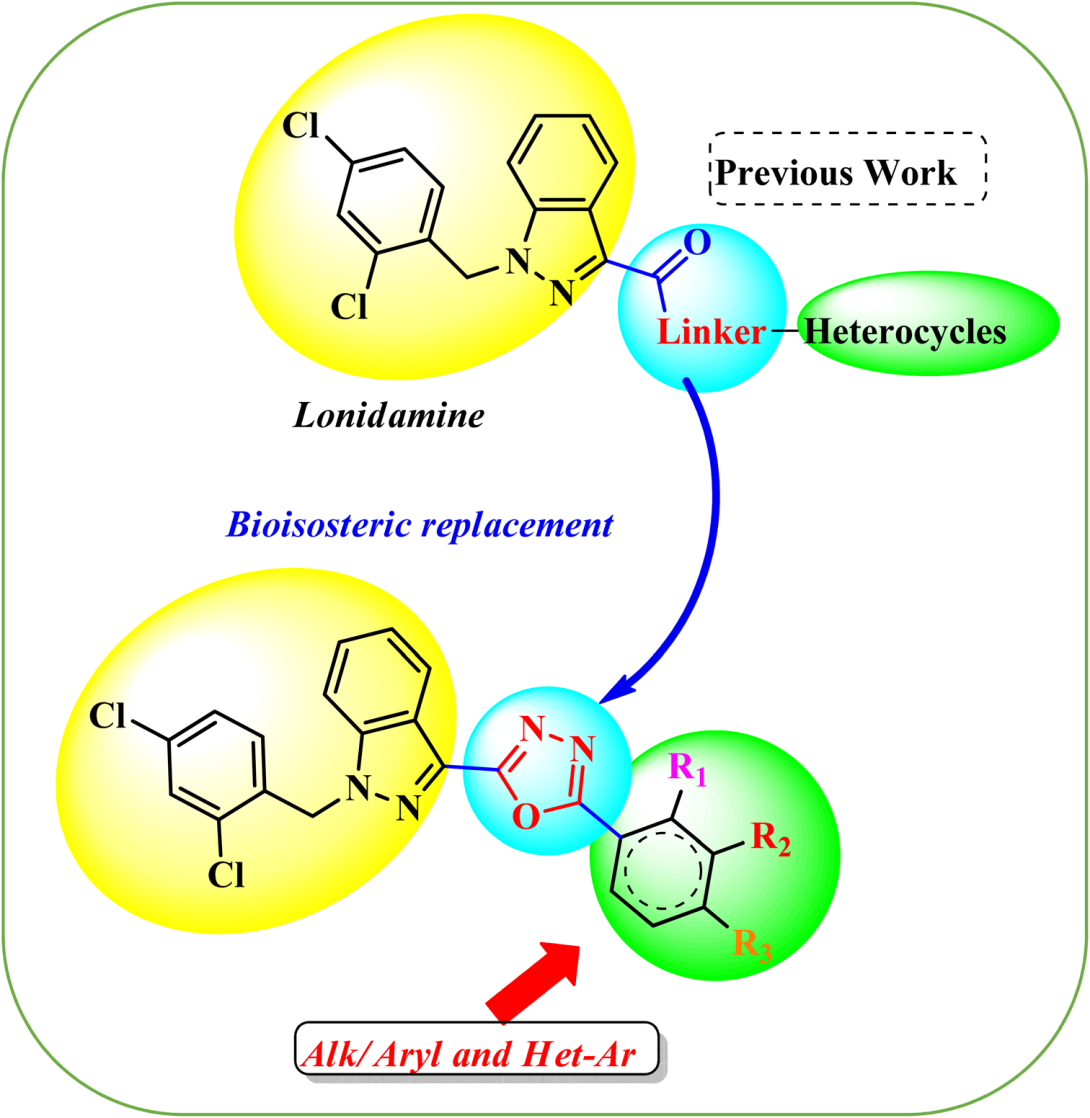
Design strategy for the targeted compounds.

LND is a well-known anti-cancer agent that disrupts glycolysis by inhibiting mitochondrial hexokinase, leading to energy depletion in tumor cells. Despite its promising activity, LND has been investigated for the treatment of various cancers such as colon cancer, breast cancer, non-small cell lung cancer (NSCLC), astrocytoma, and squamous cell carcinoma.^32^ Additionally, 1,3,4-oxadiazoles have demonstrated various mechanisms of action and have been involved in the discovery and development of anticancer drugs.^33,34^ Despite its promising activity, LND has poor water solubility and limited bioavailability, mainly because of the carboxylic acid (–COOH) group. To overcome these limitations, bioisosteric replacement strategies have been explored. The design strategy involves replacing the amide linker in LND with a 1,3,4-oxadiazole group to enhance metabolic stability and biological activity. This scaffold modification preserves the primary pharmacophoric activity of LND while allowing the insertion of a 1,3,4-oxadiazole at the terminal position. The redesigned scaffold allows for additional customization by selectively modifying substituents (R_1_, R_2_, R_3_) on aryl or heteroaryl rings to change electrical, lipophilic, and steric properties. The rational combination of LND and important 1,3,4-oxadiazole is predicted to improve target selectivity, binding affinity, and anti-proliferative effects *via* dual or increased interactions with biological targets (Fig. 2).

## 3 Results and discussion

### 3.1 Synthesis of novel bifunctional compounds for the study

**Scheme 1:**
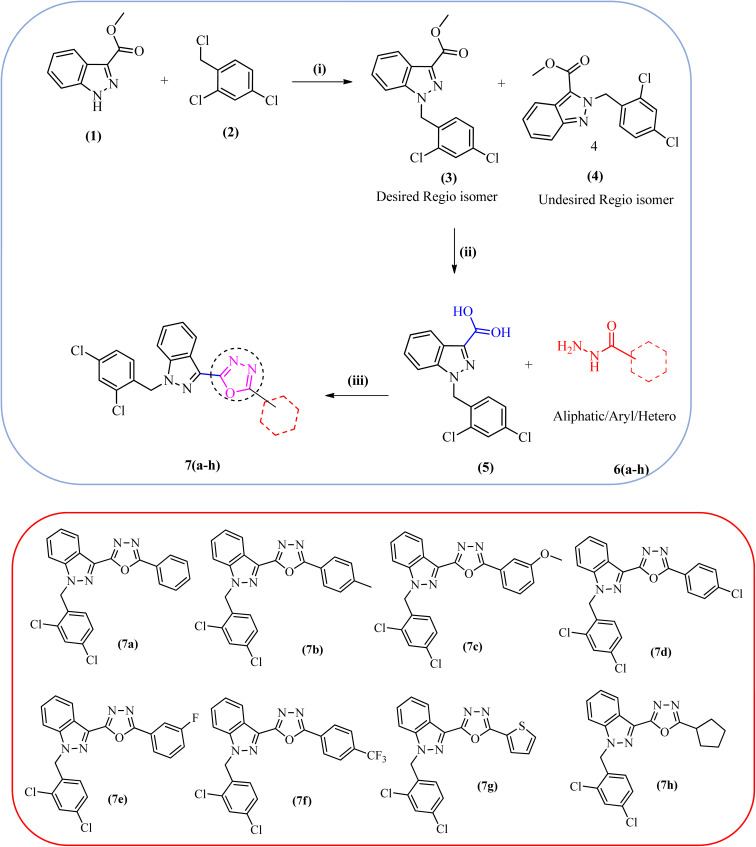
Synthetic scheme outlining the preparation of target compounds 7(a–h): (i) K_2_CO_3_, DMF, rt, 16 h, (ii) NaOH, THF: H_2_O, 70 °C, 2 h, (iii) POCl_3_ (neat), reflux.

A new series of lonidamine-1,3,4-oxadiazole compounds 7(a–h) were strategically designed and synthesized to enhance the therapeutic potential of the parent compound. The targeted compounds 7(a–h) were synthesized following the strategy outlined in Scheme 1. The synthesis commenced with the preparation of LND (5) from methyl 1*H*-indazole-3-carboxylate and 2,4-dichloro-1-(chloromethyl) benzene in the presence of potassium carbonate in DMF, using previously published approaches with minor modifications.^31^ In the next step, a series of structurally diverse hydrazides 6(a–h), including aliphatic, aromatic, and heterocyclic derivatives, were selected for condensation with LND (5). These hydrazides were chosen to introduce electronic and steric diversity into the final molecules, allowing for a comprehensive evaluation of structure–activity relationships (SAR). The reaction was carried out in the presence of phosphoryl chloride under a nitrogen atmosphere, facilitating cyclodehydration to form the desired LND-1,3,4-oxadiazole molecules 7(a–h). This design approach integrates the pharmacophoric properties of LND with the versatility and drug-like nature of the oxadiazole ring, offering a promising platform for the development of new anticancer agents.

### 3.2 Biological studies

Uncontrolled cell proliferation is a hallmark of cancer, leading to the formation of tumor masses through evasion of the programmed cell death mechanism. One promising approach in cancer therapy involves the use of small molecules to inhibit proliferation and impair the migratory and invasive potential of cancer cells, ultimately triggering cell death. In this study, we screened a library of synthesized small molecules for their anticancer activity against HCT116 colorectal cancer cells. We employed the MTT assay, a colorimetric technique that assesses mitochondrial activity to gauge cell viability and metabolic health. Table 1 displays the IC_50_ values of LND, as a reference, and synthesized compounds 7(a–h). The IC_50_ value, which indicates the concentration needed to inhibit 50% of cell viability, is a key measure of a compound's potency. Among the tested compounds, compound (7d) demonstrated the strongest anti-proliferative effect, showing an IC_50_ of 12.91 ± 1.58 µM, which indicates a significant inhibition of cell growth. The compounds (7c) and (7g) had higher potency than other compounds, with IC_50_ values of 15.43 ± 0.93 and 19.41 ± 0.68 µM, respectively (Table 1). Selectivity index of the screened drug was measured by comparing CC_50_ of small molecule against normal cells and IC_50_ against cancer cells (Table 1).

**Table 1:** IC_50_ value of the synthesized compounds 7(a–h)

IC_50_ (µM)			
Compounds	HCT116	HEK	Selectivity index
7a	25.29 ± 1.12	58.34 ± 1.78	2.31
7b	25.95 ± 1.32	52.75 ± 1.11	2.03
7c	15.43 ± 0.93	56.21 ± 1.39	3.64
7d	**12.91** ± **1.58**	72.72 ± 1.69	**5.63**
7e	22.23 ± 0.89	78.97 ± 1.53	3.55
7f	29.69 ± 1.95	89.81 ± 1.98	3.02
7g	19.41 ± 0.68	64.88 ± 1.27	3.34
7h	27.93 ± 1.88	62.38 ± 1.65	2.23
Lonidamine	88.45 ± 0.55	258.11 ± 3.21	2.92

#### 3.2.1 Compound 7d induces HCT116 cell death, hinders migration of colon carcinoma cells

**Fig. 3:**
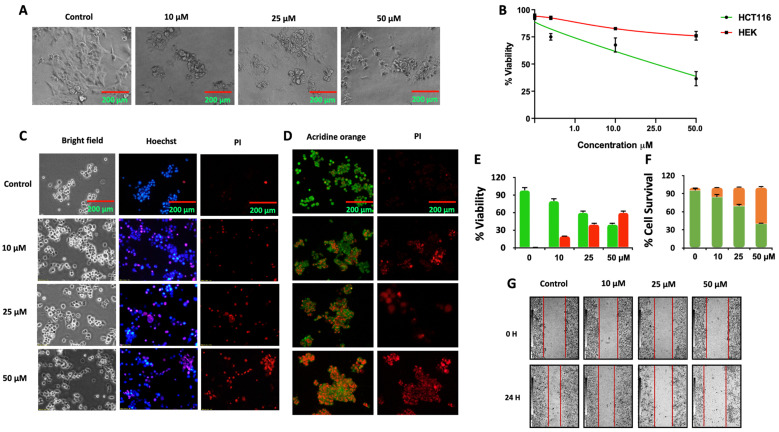
Compound 7d regresses cell proliferation, migration and induces cell death: HCT116 cells were treated with compound (7d) and subjected to anti-proliferative assays. (A) Brightfield images of HCT116 cells treated with compound (7d); (B) % viability of HCT116 treated with compound (7d); (C) Hoechst/PI analysis of HCT116 cells; (D) acridine orange/PI dual staining of HCT116 cells, (E) histogram graphs of % viable cells, (F) histogram graphs of % dead cells, (G) cell migration analysis. DMSO served as a negative control. All the assays were repeated thrice. All the images were captured using an Olympus CKX53 microscope with a scale bar of 200 µm.

The structural optimization of compound (7d), especially the incorporation of a chlorobenzene moiety into the oxadiazole scaffold, significantly improved its activity. Replacing the chlorine group with a methoxy substituent resulted in a slight decrease in potency, with an IC_50_ of 15.43 ± 0.93 µM. This emphasizes the importance of the R group in molecular efficiency. Other structural modifications at the R position additionally decreased activity, illustrating the chemical configuration's specificity. To assess the morphological effects and membrane integrity following treatment, bright field microscopy was performed. Cells treated with compound (7d) showed clear signs of altered morphology, suggesting the loss of viability (Fig. 3A).

The trypan blue dye exclusion assay further supported these observations. This assay differentiates the viable and nonviable cells by penetrating into the cytosol of the cells based on the membrane integrity. Compound (7d) induced a concentration-dependent reduction in viable cell number, confirming its cytotoxic effect (Fig. 3B). To further confirm the induction of cell death by compound (7d), Hoechst/PI dual staining was employed. Hoechst stains the nuclei of all cells, while PI selectively enters cells with compromised membranes, typically late apoptotic or necrotic cells. Treatment with compound (7d) led to visible nuclear deformations, membrane rupture, and chromatin condensation, indicating apoptotic cell death (Fig. 3C).

Similarly, acridine orange/PI (AO/PI) dual staining was conducted to distinguish between early apoptotic, late apoptotic, and necrotic cells (Fig. 3D). Acridine orange stains live and early apoptotic cells green, while PI stains late apoptotic stains due to compromised membrane integrity, further supporting the potent anti-proliferative effect of compound (7d) against colorectal cancer cells in a concentration-dependent manner (Fig. 3E and F). Cell migration and cell invasion play crucial roles in the metastasis of cancer cells to form secondary tumors. Interestingly, Compound (7d) mitigates the migration of colorectal cancer cells in a concentration-dependent manner, which was demonstrated by an *in vitro* migration assay (Fig. 3G). Compound (7d) not only restrains the proliferation of colorectal cancer cells but also hinders the migratory potential of colorectal cancer cells, exhibiting its potent anti-migratory potential and anti-proliferative potential.

### 3.3 Theoretical studies

#### 3.3.1 Molecular docking

**Fig. 4:**
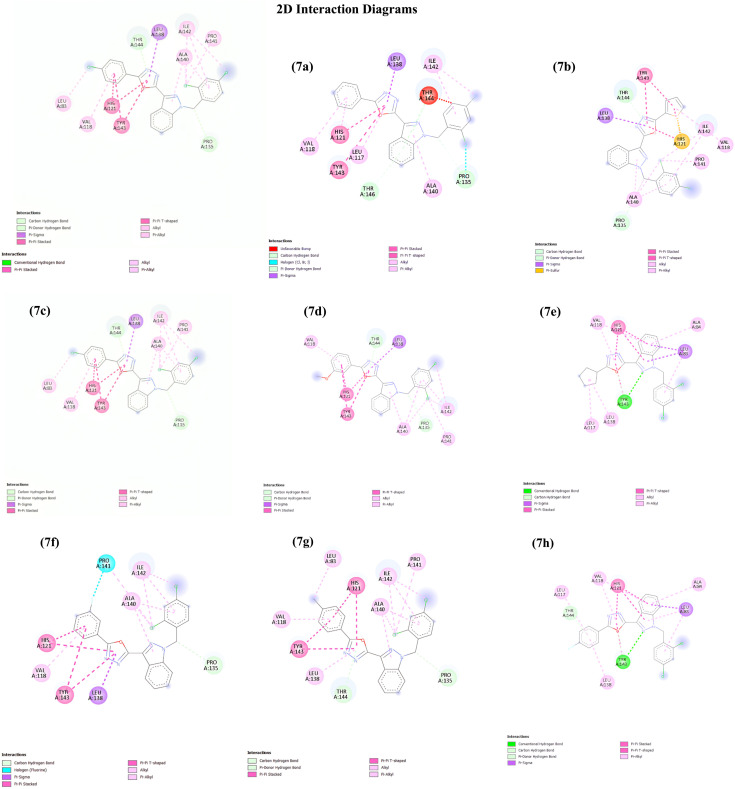
Docking analysis pose of different compounds bound to MMP2 (PDB ID: 7XJO). The chemical interactions are illustrated, highlighting the atoms of the compound and the interacting amino acids within the active site of the target protein.

Molecular docking was performed for the synthesized compounds 7(a–h) against the target protein. The binding affinities and interaction profiles were analyzed to evaluate the stability and specificity of ligand–protein complexes. The results revealed consistent hydrogen bonding, π–π interactions, hydrophobic contacts, and halogen bonds with key active site residues. Table 2 summarizes the interactions and docking scores of the synthesized compounds. The docking results revealed strong binding affinities for all compounds, with values ranging from −10.4 to −11.8 kcal mol^−1^. Compounds (7c) and (7g) exhibited the highest binding affinity (−11.8 kcal mol^−1^), suggesting strong stability within the active pocket. These ligands also displayed diverse interactions, including hydrogen bonds, π–π stacking, hydrophobic contacts, and halogen interactions. Notably, compound (7g) formed an additional π–sulfur interaction with HIS121, which may contribute to its enhanced binding. Conversely, compound (7h) showed an unfavorable bump with THR144, potentially reducing its binding stability despite a good binding affinity of −10.9 kcal mol^−1^. Overall, residues HIS121, TYR143, THR144, LEU138, ALA140, VAL118, and PRO135 emerged as critical hot spots for ligand recognition, stabilizing the complexes through a combination of polar and hydrophobic interactions. Importantly, when compared with the reference ligand LND (−8.1 kcal mol^−1^), all designed compounds 7(a–h) demonstrated superior binding affinities, ranging from −10.4 to −11.8 kcal mol^−1^. While LND interacted mainly through a hydrogen bond with TYR143, π–π stacking with HIS121, and hydrophobic stabilization by LEU83, LEU117, and VAL118, the designed ligands established more diverse and stronger interactions, including halogen bonding, π–sulfur contacts, and multiple hydrogen bonds. This indicates that the designed series not only improves binding energy but also enhances specificity and stability within the active site. Overall, the comparison highlights the potential of the designed ligands as more effective inhibitors than LND (Fig. 4).

**Table 2:** Docking scores and interactions of synthesized compounds 7(a–h)

Compounds	Binding affinity (kcal mol^−1^)	H-bonds (residues)	π–π interactions	Hydrophobic (alkyl/π-alkyl)	Halogen	Other
7a	−11.2	TYR143	HIS121, LEU83	LEU117, LEU138, VAL118	Cl–LEU83	—
7b	−10.4	THR144	HIS121, TYR143	LEU138, ALA140, ILE142, PRO141	Cl–PRO135	—
7c	−11.8	PRO141	HIS121, TYR143	LEU138, ALA140, ILE142	Cl–PRO135	—
7d	−10.8	TYR143	HIS121, LEU83	LEU117, LEU138, VAL118	Cl–LEU83	—
7e	−10.5	THR144, PRO135	HIS121, TYR143	LEU138, ALA140, ILE142, PRO141	—	—
7f	−11.2	THR144	HIS121, TYR143	LEU138, ALA140, ILE142, PRO141	Cl–PRO135	—
7g	−11.8	THR144, PRO135	HIS121, TYR143	LEU138, ALA140, ILE142, PRO141	Cl–PRO135	π–sulfur (HIS121)
7h	−10.9	THR144, PRO135	HIS121, TYR143	LEU117, LEU138, ALA140, ILE142	Cl–PRO135	Unfavorable bump (THR144)
Lonidamine (positive control)	−8.1	TYR143 (H-bond)	HIS121 (π–π stacked)	LEU83, LEU117, VAL118 (alkyl, π-alkyl)	—	Reference interactions

#### 3.3.2 Molecular dynamics simulation

##### 3.3.2.1 Root-mean-square deviation

**Fig. 5:**
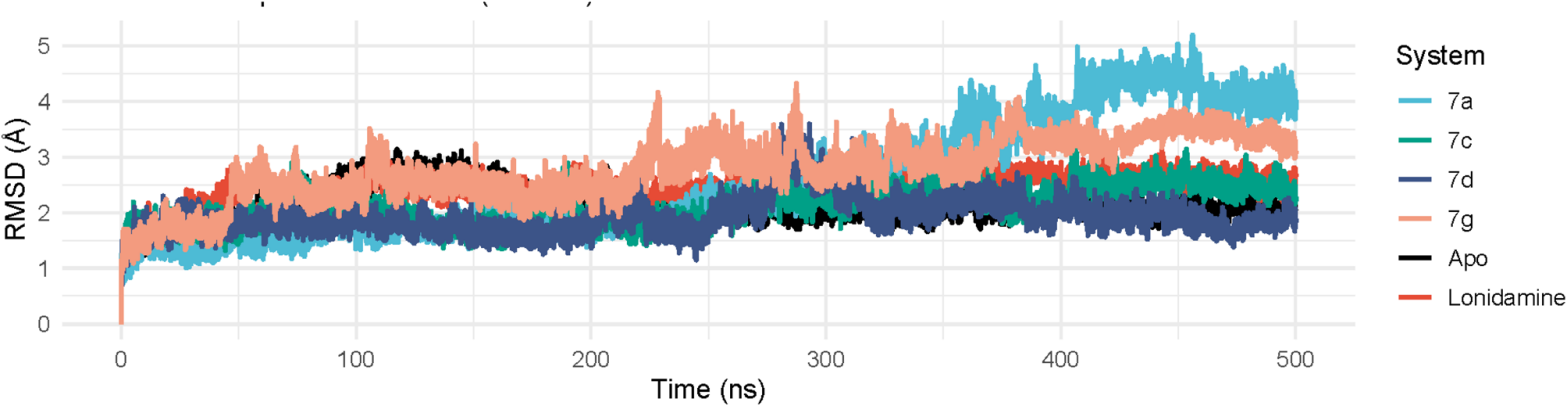
The RMSD curves of protein–ligand complexes of compounds 7a, 7c, 7d, and 7g.

Molecular dynamics (MD) simulations were carried out to evaluate the structural stability and binding behavior of the MMP2–7XJO complex with lonidamine (LND) as the positive control and the screened ligands proposed as colon cancer inhibitors. The stability of each protein–ligand system was assessed using root-mean-square deviation (RMSD) across the simulation trajectory. The MMP2–7XJO–LND complex displayed noticeable fluctuations before reaching a relatively stable regime, with RMSD values stabilizing after ∼50 ns at approximately 0.19–0.28 nm (mean ≈ 0.25 ± 0.03 nm) (Fig. 5). In contrast, the complexes formed with compounds (7c) and (7d) exhibited consistently lower RMSD profiles over the same period, remaining within 0.16–0.26 nm (7c; mean ≈ 0.22 ± 0.03 nm) and 0.16–0.27 nm (7d; mean ≈ 0.20 ± 0.03 nm), indicating reduced conformational drift and improved dynamic stability compared with LND. Conversely, compound (7g) showed higher deviations (approximately 0.22–0.36 nm, mean ≈ 0.29 ± 0.04 nm), suggesting a less stable complex under the simulated conditions, while compound (7a) exhibited larger variability overall despite intermittent low RMSD values (mean ≈ 0.27 ± 0.10 nm). Collectively, these RMSD trends support tighter and more persistent binding for 7d (best), followed by 7c, relative to the reference inhibitor LND, highlighting compound 7d as the most promising candidate for further validation as an MMP2 inhibitor with potential relevance to colon cancer therapy.

##### 3.3.2.2 Root-mean-square fluctuation (RMSF)

**Fig. 6:**
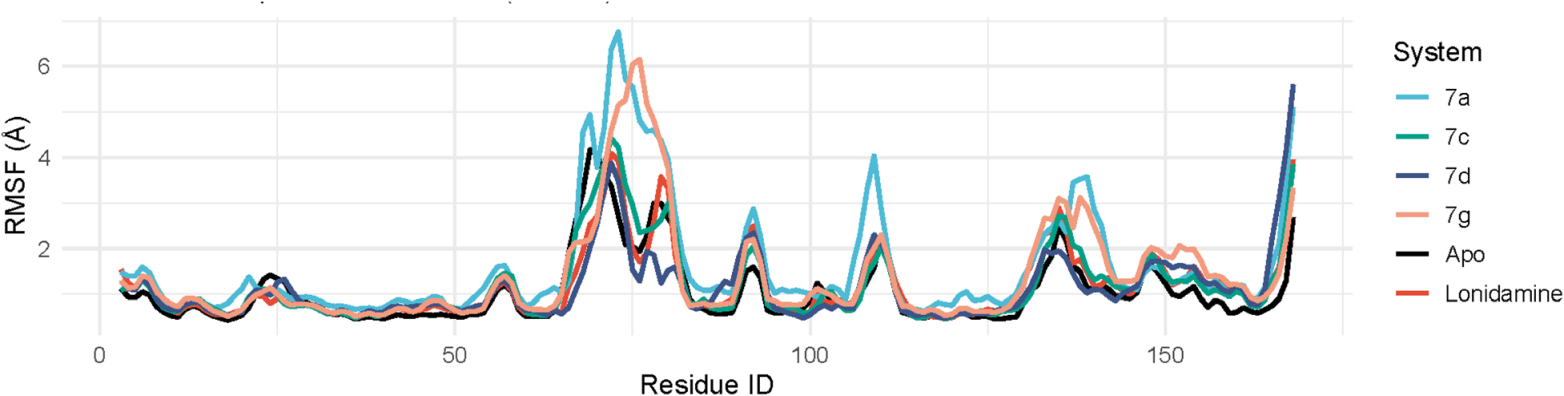
The RMSF curves of protein–ligand complexes of compounds 7a, 7c, 7d, and 7g.

The RMSF profile indicates that the protein remains largely stable across the trajectory, with flexibility concentrated in two main regions. The dominant dynamic hotspot is the loop spanning GLU69–ASP80 (GLU69, HIS70, GLY71, ASP72, GLY73, TYR74, PRO75, PHE76, ASP77, GLY78, LYS79, ASP80), which shows the highest residue mobility and represents the primary “breathing” segment of the structure. Across the simulated systems, the maximum fluctuations occur within this loop for most conditions: 7g peaks at PHE76 (6.1517 Å), 7c peaks at ASP72 (4.4247 Å), 7a peaks at GLY73 (6.7580 Å), and lonidamine peaks at ASP72 (4.0913 Å), confirming that residues ASP72/GLY73/TYR74/PRO75/PHE76 are repeatedly the most flexible core of this region. In contrast, 7d shows its strongest peak at the C-terminus (ASP168 = 5.5965 Å), with elevated motion also at PRO167, consistent with the expected intrinsic flexibility of terminal residues (SER166–PRO167–ASP168) (Fig. 6). A secondary moderate-mobility site is observed around PRO135–MET139, appearing notably in the lonidamine system (*e.g.*, PRO135), suggesting an additional loop/turn region that may respond to ligand-dependent conformational changes. Overall, the RMSF pattern supports a stable structural core with localized flexibility dominated by the GLU69–ASP80 loop, while terminal residues (166–168) contribute condition-dependent fluctuations, particularly in 7d.

##### 3.3.2.3 Radius of gyration (*R*_g_)

The radius of gyration (*R*_g_) was evaluated over 500 ns to assess the global compactness and overall structural stability of the protein in the simulated systems (7a, 7c, 7d, 7g, and lonidamine). Overall, most systems maintained a relatively stable *R*_g_ around ∼14.9–15.0 Å, indicating preservation of the global fold with only minor fluctuations. In particular, 7c (14.93 ± 0.10 Å) and 7d (14.92 ± 0.12 Å) exhibited the lowest variability, suggesting that these complexes remain consistently compact throughout the simulation. The lonidamine-bound system also retained a stable compact profile (14.95 ± 0.12 Å), supporting minimal ligand-induced global expansion.

**Fig. 7:**
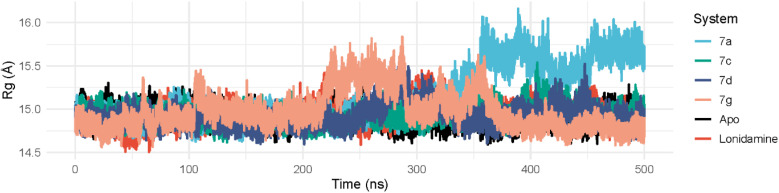
The radius of gyration analysis of compounds 7a, 7c, 7d, and 7g.

By contrast, the 7a trajectory displayed the most pronounced time-dependent change in compactness (15.15 ± 0.32 Å overall). During the first 0–300 ns, 7a remained comparable to the other systems (14.93 ± 0.09 Å), but a clear transition occurred after 300 ns, where *R*_g_ increased to a higher state (15.48 ± 0.25 Å) and remained elevated through the late-stage window (350–500 ns: 15.59 ± 0.16 Å), with occasional excursions reaching 16.16 Å (Fig. 7). This pattern suggests a distinct global expansion event in 7a, consistent with ligand-associated or conformational rearrangements that increase the overall molecular dimensions. The 7g system showed intermediate variability (14.98 ± 0.22 Å) with transient expansions early to mid-trajectory (maximum 15.84 Å), followed by a more compact late-stage behavior (350–500 ns: 14.85 ± 0.16 Å). Taken together, the *R*_g_ analysis indicates that the protein remains globally stable and compact in most systems (7c, 7d, and lonidamine), whereas 7a undergoes a marked compactness shift toward a more expanded conformational state, potentially reflecting ligand-dependent opening or broader structural reorganization.

##### 3.3.2.4 Molecular mechanics/generalized Born surface area (MM/GBSA) binding free energy analysis

The results from the MM/GBSA calculations indicate that the protein's binding affinity varies significantly across the different systems. System 7a exhibited the most favorable binding with a total free energy (Δ*G*_total_) of −33.17 ± 2.81 kcal mol^−1^, reflecting a stronger binding interaction. This system showed substantial contributions from both the van der Waals (Δ*E*_vdW_ = −44.12 ± 3.25 kcal mol^−1^) and electrostatic (Δ*E*_ele_ = −6.89 ± 2.67 kcal mol^−1^) components, which were balanced by a solvation free energy (Δ*G*_solv_ = 17.84 ± 2.10 kcal mol^−1^) that did not significantly reduce its overall stability. In comparison, 7c demonstrated weaker binding (Δ*G*_total_ = −27.57 ± 4.88 kcal mol^−1^) with more significant contributions from solvation (Δ*G*_solv_ = 15.96 ± 5.97 kcal mol^−1^), suggesting that while the van der Waals and electrostatic contributions (Δ*E*_vdW_ = −35.29 ± 5.60 kcal mol^−1^, Δ*E*_ele_ = −8.24 ± 5.06 kcal mol^−1^) were favorable, the solvation free energy did not contribute sufficiently to stabilize the protein–ligand complex. Similarly, 7d showed a balanced energy distribution (Δ*G*_total_ = −30.33 ± 2.43 kcal mol^−1^), with moderate contributions from van der Waals and electrostatic energies, although the solvation energy (Δ*G*_solv_ = 18.12 ± 2.71 kcal mol^−1^) was slightly higher compared to 7a. The 7g system (Δ*G*_total_ = −32.95 ± 3.02 kcal mol^−1^) exhibited a similar trend with substantial van der Waals and electrostatic energy contributions, but with a less favorable solvation energy (Δ*G*_solv_ = 17.99 ± 2.70 kcal mol^−1^) (Table 3). This suggests that the system may exhibit slightly weaker binding than 7A, but still maintains a strong affinity. Finally, the lonidamine-bound system showed the highest solvation energy (Δ*G*_solv_ = 26.46 ± 3.15 kcal mol^−1^), but its overall binding affinity was still relatively strong (Δ*G*_total_ = −33.22 ± 2.90 kcal mol^−1^), which indicates that the ligand's influence on the protein–ligand binding dynamics is substantial despite the high solvation contributions.

**Table 3:** MM/GBSA binding free energy and energetic components (kcal mol^−1^, mean ± SD)^a^

Systems	Δ*E*_vdW_	Δ*E*_ele_	Δ*E*_GB_	Δ*E*_surf_	Δ*G*_gas_	Δ*G*_solv_	Δ*G*_total_
7a	−44.12 ± 3.25	−6.89 ± 2.67	20.97 ± 2.13	−3.13 ± 0.22	−51.01 ± 3.37	17.84 ± 2.10	−33.17 ± 2.81
7c	−35.29 ± 5.60	−8.24 ± 5.06	18.93 ± 6.28	−2.97 ± 0.41	−43.53 ± 9.66	15.96 ± 5.97	−27.57 ± 4.88
7d	−44.72 ± 2.86	−3.73 ± 3.41	21.53 ± 2.72	−3.41 ± 0.16	−48.45 ± 3.67	18.12 ± 2.71	−30.33 ± 2.43
7g	−43.68 ± 3.11	−7.26 ± 2.89	21.08 ± 2.82	−3.10 ± 0.27	−50.93 ± 4.11	17.99 ± 2.70	−32.95 ± 3.02
Lonidamine	−42.01 ± 2.63	−17.68 ± 3.65	29.37 ± 3.24	−2.90 ± 0.17	−59.68 ± 4.85	26.46 ± 3.15	−33.22 ± 2.90

a Δ*E*_vdW_: van der Waals energy; Δ*E*_ele_: electrostatic energy; Δ*G*_GB_: solvation-free energy change part based on the generalized Born model; Δ*E*_surf_: free energy change contributed by the surface area; Δ*G*_gas_: change in free energy in the gas phase; Δ*G*_solv_: solvation free energy change; Δ*G*_binding_: total free energy change. Relationship: Δ*G*_total_ = Δ*G*_gas_ + Δ*G*_solv_ = Δ*E*_vdW_ + Δ*E*_ele_ + Δ*E*_GB_ + Δ*E*_surf_.

#### 3.3.3 Principal component analysis

##### 3.3.3.1 2D projection of trajectories

**Fig. 8:**
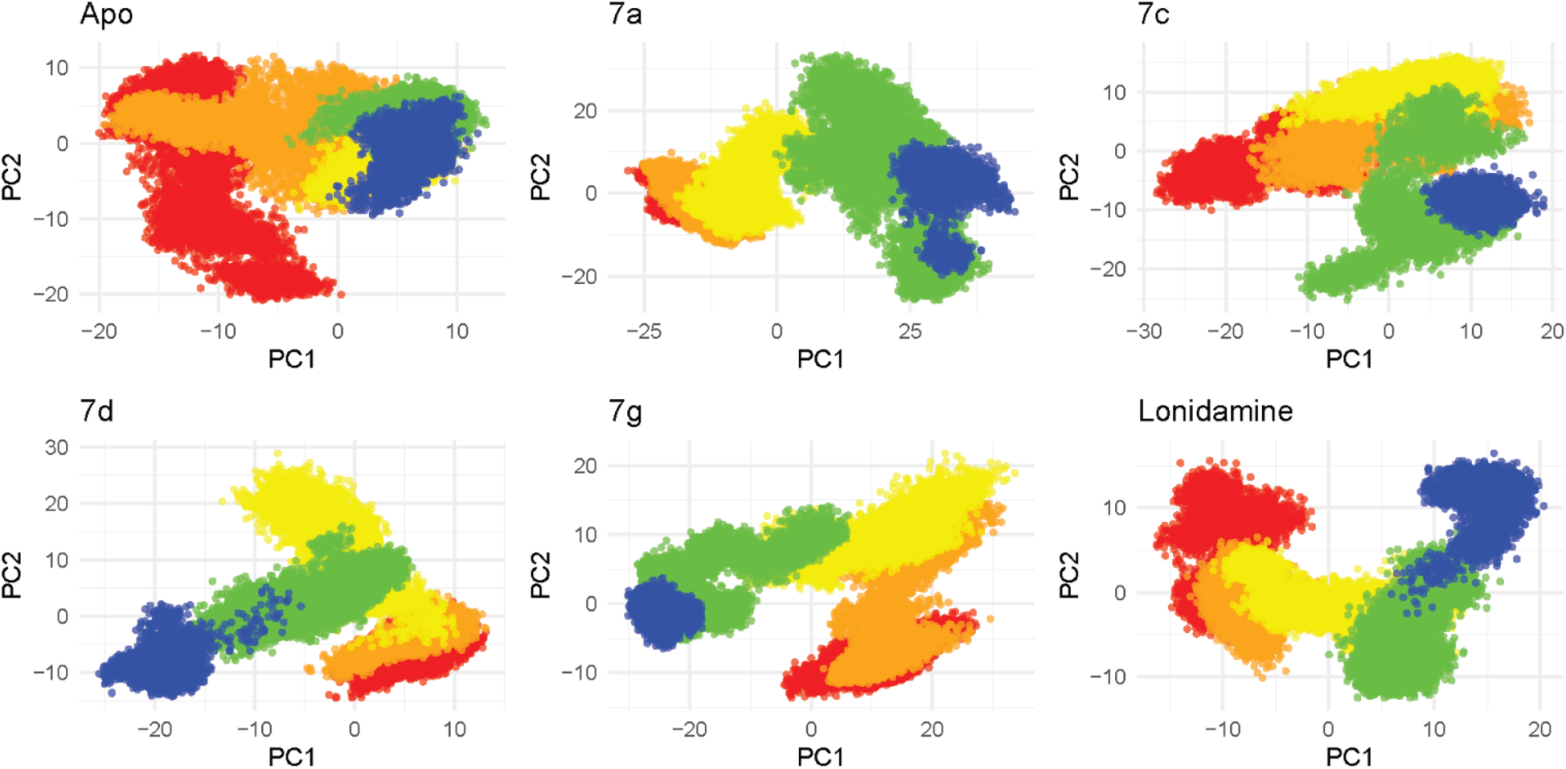
2D projection of trajectory compounds 7a, 7c, 7d, and 7g.

Principal component analysis (PCA) of the MD trajectories (50 000 frames) demonstrated ligand-dependent reshaping of the sampled conformational landscape. The apo system exhibited the most compact distribution in the PC1–PC2 subspace (total variance, Var(PC1) + Var(PC2) = 94.120), whereas ligand binding increased dispersion to varying extents. Among the complexes, 7a showed the broadest sampling (total variance = 448.276; PC1 range −28.041 to 44.659; PC2 range −25.760 to 33.218), followed by 7g (total variance = 316.281), indicating substantially enhanced conformational heterogeneity relative to apo. Compounds 7c and 7d occupied intermediate conformational space (157.984 and 131.239, respectively), while lonidamine displayed a comparatively confined distribution (116.614). A two-center separation check further supported distinct population structure for 7a and 7g (center distances 37.77 and 31.74), consistent with sampling of multiple dominant conformational regions, whereas 7d showed the weakest separation (13.14), indicating more limited partitioning within the reduced space (Fig. 8).

##### 3.3.3.2 Free energy landscape (FEL)

**Fig. 9:**
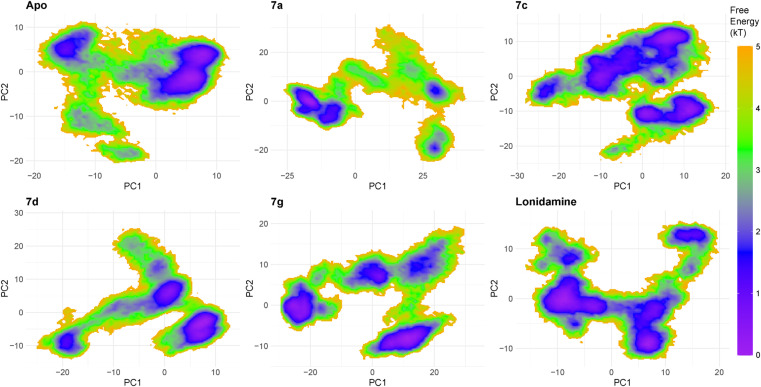
FEL 3D of compounds 7a, 7c, 7d and 7g.

The FEL projected onto PC1–PC2 revealed ligand-dependent reshaping of the conformational basins. The apo system displayed multiple low-energy minima, indicating the presence of more than one metastable conformation, although these states were confined within a comparatively bounded region of PC space. In contrast, 7a produced the most expanded and distinctly partitioned landscape, with well-separated low-energy basins across negative and positive PC1, supporting pronounced multi-state behavior. Compound 7g similarly exhibited several low-energy minima distributed across PC space, consistent with dynamic sampling of multiple substates and potential interconversion along connecting corridors. Compounds 7c and 7d showed intermediate landscapes with two to several dominant basins, suggesting moderate conformational heterogeneity under ligand binding. lonidamine displayed a dominant low-energy basin accompanied by secondary minima, indicating stabilization of a preferred conformational state while retaining access to alternate substrates (Fig. 9).

### 3.4 *In silico* evaluation of physicochemical and ADME properties

A series of compounds 7(a–h) were evaluated based on their physicochemical properties, pharmacokinetic profiles, drug-likeness, and synthetic accessibility to assess their potential as orally active drug candidates using SwissADME.^35^ The key parameters analyzed included hydrogen bond acceptors (HBA), hydrogen bond donors (HDA), topological polar surface area (TPSA), lipophilicity (log *P*), aqueous solubility (log *S*), gastrointestinal absorption (GIA), skin permeability (log *K*_p_), Lipinski's rule compliance, and synthetic accessibility (SA) (Table 4). All of these compounds fulfilled Lipinski's rule of five, suggesting excellent drug-likeness. Notably, compound (7h) emerged as the most promising option, with no violations of Lipinski's rule,^36^ strong gastrointestinal absorption, and a modest log *P* (5.09), indicating a desirable balance of hydrophilicity and lipophilicity. In contrast, compound (7f) had the highest lipophilicity (log *P* = 6.11) and the lowest water solubility (log *S* = −7.48), which corresponded to its low anticipated oral absorption. Despite complying with Lipinski's requirements, this makes it a less favorable candidate. All compounds have TPSA values ≤ 140 Å^2^, indicating acceptable membrane permeability. Compound (7g) exhibited the largest TPSA (84.98 Å^2^), which may affect its permeability, although it maintained high GIA.^37^ The synthetic accessibility (SA) scores varied from 3.17 to 3.37 across the series, indicating that all compounds are reasonably easy to synthesis, with compounds (7a) and (7e) having slightly lower SA values (3.17), indicating a more favorable synthetic profile.^38^ In terms of skin permeability, all compounds had relatively low log *K*_p_ values (−4.65 to −5.72), which is typical for drug-like molecules and not a limiting factor for drug development. Overall, compound (7h) has the most balanced and positive profile across all parameters tested, making it a promising candidate for additional preclinical testing.

**Table 4:** Predicted ADME parameters of compounds

Comp.	Physiochemical properties					Pharmacokinetics		Drug likeness		Medicinal chemistry
	HBA	HDA	TPSA	Log *P*_o_/*w*	Log *S*	GIA	Log *K*_p_	Lipinski	Vio	SA
7a	4	0	56.74	5.11	−6.56	High	−4.89	Yes	1	3.17
7b	4	0	56.74	5.51	−6.94	High	−5.72	Yes	1	3.31
7c	5	0	65.97	5.14	−6.73	High	−5.09	Yes	1	3.29
7d	4	0	56.74	5.66	−7.22	High	−4.65	Yes	1	3.18
7e	5	0	56.74	5.40	−6.67	High	−4.93	Yes	1	3.18
7f	7	0	56.74	6.11	−7.48	Low	−4.68	Yes	1	3.32
7g	4	0	84.98	5.10	−6.87	High	−5.12	Yes	1	3.24
7h	4	0	56.74	5.09	−6.56	High	−4.84	Yes	0	3.37

**Fig. 10:**
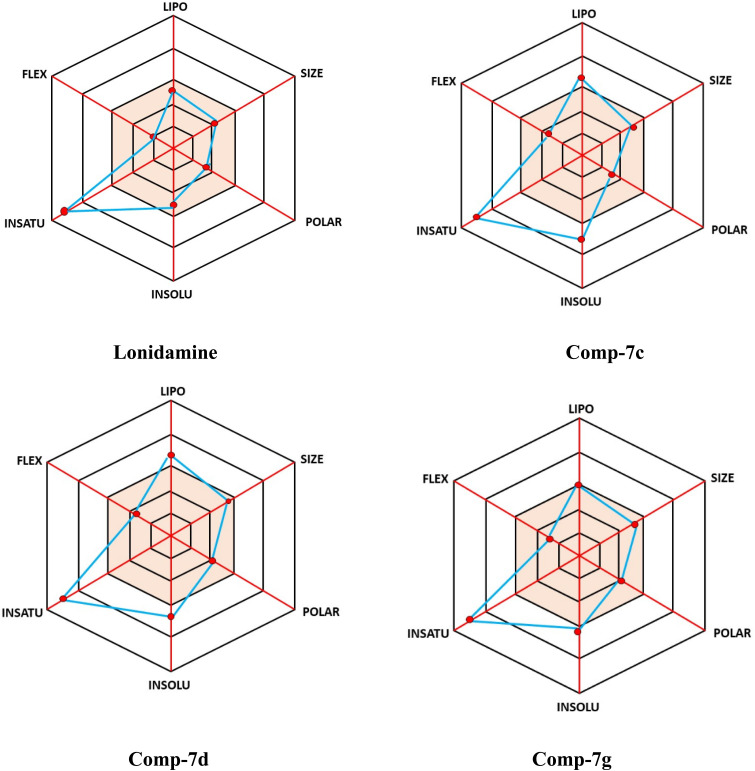
Physiochemical space for the oral bioavailability of LND and compounds 7c, 7d, and 7g.

Bioavailability radar plots displaying six essential physicochemical parameters related to oral bioavailability: lipophilicity (LIPO), size, polarity (POLAR), solubility (INSOLU), saturation (INSATU), and molecular flexibility (FLEX). The pink region shows the ideal drug-likeness range. The radar plot analysis offers a clear visual comparison of the biological and physicochemical profiles of LND and its analogues. LND has a balanced but moderate profile across most evaluated parameters, which reflects its established but limited activity. The analogues show clear differences, highlighting how structural changes impact their properties. Among the analogues, compound (7d) stands out with the best profile. It has higher scores across several descriptors, suggesting it may be more potent and drug-like. Compound (7c) shows moderate improvements over LND but is still less effective than (7d). Compound (7g) presents a different pattern, demonstrating strong activity in some areas while performing weaker in others, indicating potential selectivity. The other analogues show varying trends, suggesting that adjusting substituents can lead to different activity and toxicity outcomes. In summary, the radar analysis indicates that substitution patterns significantly affect the physicochemical and biological behavior of the oxadiazole derivatives. Compound (7d) is the most promising analogue when compared to LND (Fig. 10).

**Fig. 11:**
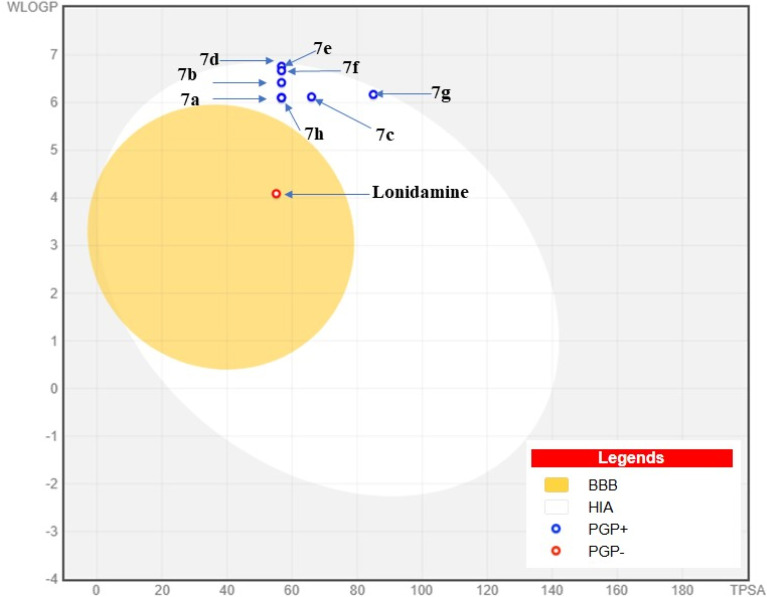
The BOILED-Egg diagram illustrates the predicted gastrointestinal absorption (HIA) and blood–brain barrier (BBB) penetration of lonidamine and 7(a–h).

The BOILED-Egg diagram provides a visual prediction of the gastrointestinal absorption (HIA) and blood–brain barrier (BBB) penetration for compounds 7(a–h) and LND.^39^ In this plot, the white region (egg white) represents compounds likely to be absorbed in the gastrointestinal tract, while the yellow region (yolk) indicates the potential to cross the blood–brain barrier. Compounds 7(a–h) are expected to have high gastrointestinal absorption, indicated by the white ellipse. However, they stay outside the BBB penetration zone, marked by the yellow region, which means there is limited exposure to the central nervous system (CNS). Notably, compound (7d), the most active in the series, is well within the optimal HIA region and outside the BBB zone. This suggests it has good oral absorption and a lower chance of causing side effects in the CNS (Fig. 11).

### 3.5 Toxicity

A theoretical analysis of the toxicity of the LND derivatives 7(a–h), alongside the LND, was conducted using Protox-II,^40^ with the results presented in Table 5. The LD_50_ values show that all tested compounds fall within toxicity class 4, which means they are harmful if swallowed, according to the GHS classification. Most compounds show an inactive profile for hepatotoxicity, carcinogenicity, mutagenicity, and cytotoxicity. Only a few predictions of immunotoxicity or hepatotoxicity are noted, such as for (7c) and (7e). Notably, compound (7d), the most active of the sequence, has a completely inert toxicity profile across all endpoints. This provides a sufficient safety margin for future development.

**Table 5:** Toxicity predication of compounds 7(a–h)

Comp. code	Predicted LD_50_ (mg kg^−1^)	Predicted toxicity class	Hepatotoxicity	Carcinogenicity	Immunotoxicity	Mutagenicity	Cytotoxicity
5	900	4	Active (0.53)	Inactive (0.84)	Inactive (0.98)	Inactive (0.86)	Inactive (0.67)
7a	1140	4	Inactive (0.58)	Inactive (0.65)	Inactive (0.96)	Inactive (0.68)	Inactive (0.78)
7b	1140	4	Inactive (0.57)	Inactive (0.66)	Inactive (0.97)	Inactive (0.68)	Inactive (0.76)
7c	1140	4	Active (0.54)	Inactive (0.66)	Active (0.90)	Inactive (0.63)	Inactive (0.70)
7d	1140	4	Inactive (0.58)	Inactive (0.65)	Inactive (0.91)	Inactive (0.68)	Inactive (0.78)
7e	1140	4	Active (0.57)	Inactive (0.65)	Active (0.63)	Inactive (0.66)	Inactive (0.78)
7f	1140	4	Inactive (0.54)	Inactive (0.63)	Inactive (0.85)	Inactive (0.64)	Inactive (0.76)
7g	1140	4	Inactive (0.54)	Inactive (0.65)	Inactive (0.98)	Inactive (0.62)	Inactive (0.74)
7h	1140	4	Inactive (0.53)	Inactive (0.64)	Inactive (0.94)	Inactive (0.62)	Inactive (0.76)

**Fig. 12:**
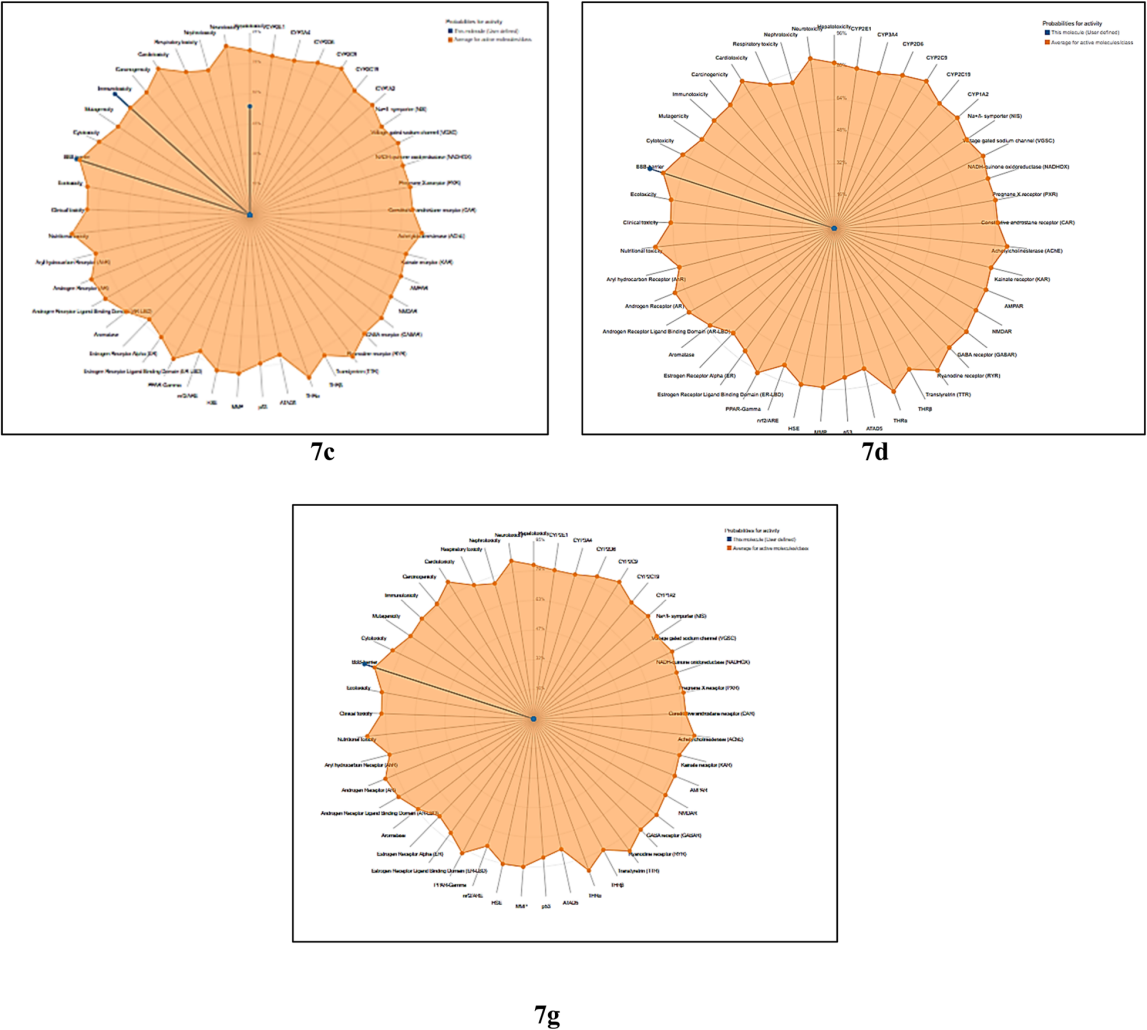
Radar plots representing the predicted biological activity profiles of compounds 7c, 7d, and 7g using the ProTox-II platform. The shaded regions indicate the probability scores of each molecule (blue) relative to the average probability of active molecules (orange) across various toxicity endpoints and receptor targets.

The toxicity prediction models for compounds (7c), (7d), and (7g) revealed significant variances in toxicity profiles. Compound (7c) had probable hepatotoxicity (probability 0.54) and immunotoxicity (0.90), but it was neither carcinogenic, mutagenic, or cytotoxic. It also confirmed blood–brain barrier (BBB) permeability with high confidence (0.90). In contrast, compound (7d) demonstrated a superior profile, being inactive for all examined endpoints except BBB permeability (0.95), indicating that it is safer than (7c) (Table 6 and Fig. 12). Also, the compounds (7c), (7d), and (7g) shows clear safety trends within the group. Compound (7c) is non-carcinogenic, non-mutagenic, and non-cytotoxic. However, it has potential liver toxicity and significant immune-related risks, which may limit how it can be used therapeutically. On the other hand, compound (7d) has a better profile. It shows inactivity in all major toxicity areas and has strong permeability through the blood–brain barrier, making it suitable for targeting the central nervous system. Among the three, compound (7g) has the most appealing profile, with all toxicity end points predicted to be inactive, including immune toxicity, and a high chance of penetrating the blood–brain barrier. Overall, these findings suggest that (7g) is the safest and most promising candidate. Compound (7d) also has positive qualities, while (7c) raises concerns due to its immune and liver risks.

**Table 6:** The toxicity model for the compounds 7c, 7d, and 7g

Classification	Target	Shorthand	Prediction			Probability		
			7c	7d	7g	7c	7d	7g
Organ toxicity	Hepatotoxicity	Dili	Active	Inactive	Inactive	0.54	0.58	0.54
Toxicity end points	Carcinogenicity	Carcino	Inactive	Inactive	Inactive	0.66	0.65	0.65
Toxicity end points	Immunotoxicity	Immuno	Active	Inactive	Inactive	0.90	0.91	0.98
Toxicity end points	Mutagenicity	Mutagen	Inactive	Inactive	Inactive	0.63	0.68	0.62
Toxicity end points	Cytotoxicity	Cyto	Inactive	Inactive	Inactive	0.70	0.78	0.74
Toxicity end points	BBB-barrier	BBB	Active	Active	Active	0.90	0.95	0.94

### 3.6 Plausible structural–activity relationship (SAR) study

**Fig. 13:**
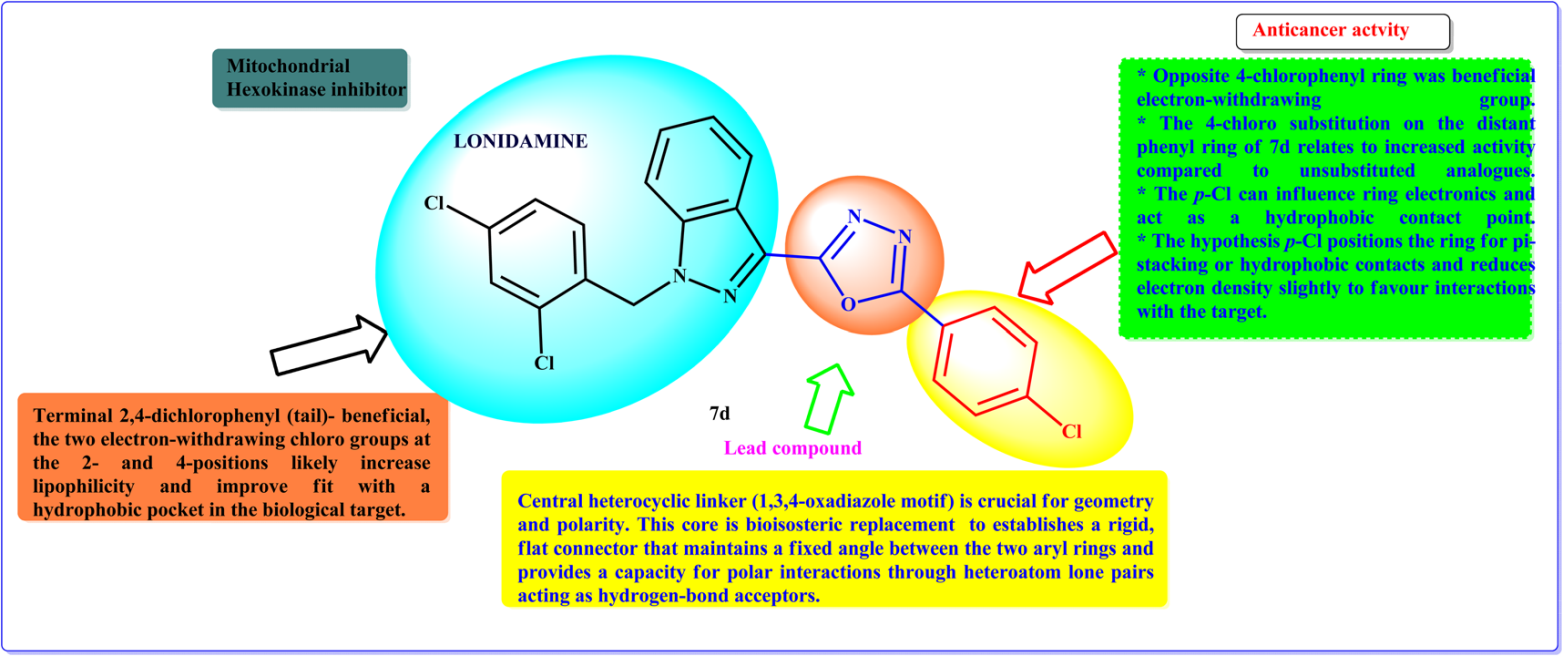
Plausible SAR of lonidamine-1,3,4-oxadiazole derivatives.

The SAR is based on the synthesized compounds indicated in Scheme 1 and their correlation with biological activity (Table 1). The cytotoxic effect study provided vital insights into the SAR that governs their anticancer efficacy. Compound (7d) is the most active analogue of the LND-1,3,4-oxadiazole family, and its potency stems from three structural properties. The terminal 2,4-dichlorophenyl group enhances lipophilicity and hydrophobic fit inside the binding pocket. This corresponds to the peak activity recorded. The distal 4-chlorophenyl ring enhances hydrophobic contacts and alters electronic properties, promoting π-stacking and target engagement. The central oxadiazole linker is important because it keeps the two aryl rings aligned and offers hydrogen-bond acceptor capability. All of these features work together to provide an ideal shape and polarity for high binding affinity. This explains why (7d) exhibits more activity than its other counterparts. However, while the numerous-chloro substituents improve potency, they may have a deleterious impact on solubility and metabolic clearance. This emphasizes the importance of careful optimization in achieving a balance between efficacy and drug-like features (Fig. 13).

## 4 Conclusion

This study focuses on the design and synthesis of a series of lonidamine and 1,3,4-oxadiazole derivatives by substituting the amide linker with a 1,3,4-oxadiazole scaffold. The compounds were evaluated for their ability to inhibit the growth of the HCT116 colorectal cancer cells. Among the screened compounds, compound (7d) exhibited significant anticancer activity, showing the lowest IC_50_ value of 12.91 ± 1.58 µM, in contrast to the parent compound LND, which had an IC_50_ of 88.45 ± 0.55 µM. The induction of apoptosis by compound (7d) against HCT116 confirms its role as a potent anticancer agent. Fluorescence microscopic studies showed that the compound induces cell death through apoptosis, not *via* necrosis. Additional computational studies, including molecular docking, molecular dynamics simulations, and DFT analysis, provided insights into how these compounds interact with the target protein, supporting their biological activity. All designed ligands 7(a–h) showed stronger binding affinities (−10.4 to −11.8 kcal mol^−1^) than the positive control LND (−8.1 kcal mol^−1^). Compounds (7c) and (7g) proved to be the most potent, exhibiting various stabilizing interactions. DFT analysis provided insights into the chemical reactivity and binding potential of the compounds, while molecular electrostatic potential maps highlighted favorable interaction sites. Furthermore, ADME profiling and toxicity predictions indicated excellent pharmacokinetic properties and acceptable safety margins, suggesting these molecules as promising drug candidates. Structure–activity relationship analysis identified key structural features necessary for activity, providing useful guidance for designing improved analogs. Overall, this research identifies compound (7d) as a promising lead for further optimization and preclinical testing in the treatment of colorectal cancer. Future studies (*in vivo*) aimed at enhancing the cytotoxicity of these compounds will be published later.

## 5 Experimental

### 5.1 Chemistry

#### 5.1.1 General methods and materials

Analytical-grade reagents were purchased from Sigma-Aldrich, Fluka, and Acros and were used as supplied. Solvents were dried according to standard methods. The chemical reactions were monitored by TLC using alumina plates coated with silica gel 60 F254 (Merck). Column chromatography separations were performed on CombiFlash by using a Silicycle RediSep® flash column. The melting points (m.p.) were measured with a Leica Galen III hot stage apparatus and are uncorrected. The ^1^H and ^13^C NMR spectra were recorded on a Bruker Avance Neo 400 MHz spectrometer with a 5 mm BBO/H–F broadband Prodigy cryoprobe with Topspin 4.1.1 software. Chemical shifts (d) are reported in ppm from the standard internal reference tetramethylsilane (TMS). The following abbreviations are used: s = singlet, d = doublet, t = triplet, and m = multiplet. Mass spectra (ESI-MS) were performed on a Shimadzu_2020 and Agilent_1290 mass spectrometer equipped with an ESI ion source, operated in the positive and negative ion mode. The ^1^H NMR and ^13^C NMR spectra of the final compounds are presented as SI material.

#### 5.1.2 General procedure for the preparation of 1-(2,4-dichlorobenzyl)-1*H*-indazole-3-carboxylic acid/lonidamine (5)

In the typical process of synthesis, methyl 1*H*-indazole-3-carboxylate (1) (56.79 mmol) were dissolved in 100 mL of acetone and added K_2_CO_3_ (68.15 mmol), and stirred for 20 min. 2,4 dichlorobenzyl chloride (62.46 mmol) and reaction mixture was heated at 60 °C for 16 h. Upon completion of the reaction mixture was cooled to room temperature and filtered. The filtrate was concentrated and products were isolated by using Combi-flash® Redisep column chromatography eluting with 7–8% EtOAc/*n*-hexane to obtain solid, 2-(3,5-dichlorobenzyl)-2*H*-indazole-3-carboxylate, regioisomer −1 (3) (undesired, minor) (2.1 g, 11%, white solid) followed by elution with 25–30% EtOAc/*n*-hexane to obtain methyl 1-(2,4-dichlorobenzyl)-1*H*-indazole-3-carboxylate regioisomer-2 (4) (desired, major) as a white solid. Yield: 84%. ^1^H NMR (400 MHz, CDCl_3_-*d*) *δ*: 8.27 (td, *J* = 1.0, 8.1 Hz, 1H), 7.45–7.27 (m, 4H), 7.08 (dd, *J* = 2.1, 8.4 Hz, 1H), 6.68 (d, *J* = 8.5 Hz, 1H), 5.78 (s, 2H), 4.05 (s, 3H). The desired product regioisomer-2 (4), which was isolated from step-1 [methyl 1-(2,4-dichlorobenzyl)-1*H*-indazole-3-carboxylate] (44.90 mmol) was hydrolyzed by using 2 M aqueous NaOH (150 mL) in THF (100 mL) heated at 60 °C for 4 h. Upon completion of the reaction mixture (reaction is monitored by TLC) was concentrated and solvents are removed and obtained residue was acidified with 3 M HCl. The precipitated solid was filtered and dried under vacuum to give pure 1-(2,4-dichlorobenzyl)-1*H*-indazole-3-carboxylic acid (lonidamine) (5) as a white solid. Yield: 90%; m.p.205 °C; ^1^H NMR (400 MHz, DMSO-*d*_6_) *δ*: 13.13 (br s, 1H), 8.12 (d, *J* = 8.2 Hz, 1H), 7.81 (d, *J* = 8.5 Hz, 1H), 7.70 (d, *J* = 2.2 Hz, 1H), 7.50 (ddd, *J* = 1.0, 7.0, 8.4 Hz, 1H), 7.39 (dd, *J* = 2.2, 8.4 Hz, 1H), 7.35 (t, *J* = 7.2 Hz, 1H), 6.96 (d, *J* = 8.4 Hz, 1H), 5.84 (s, 2H); MS-ESI (*m*/*z*): 320.95 (M + 1)^+^; HPLC: 98.54%; RT: 4.541 min.^31^

#### 5.1.3 General procedure preparation of lonidamine-1,3,4-oxadiazole compounds 7(a–h)

The commercially available aliphatic/aryl/hetero hydrazides 6(a–h) and 1-(2,4-dichlorobenzyl)-1*H*-indazole-3-carboxylic acid (lonidamine) (5) were dissolved in POCl_3_ (10 mL), and the reaction mixture was heated to 110 °C for 4–6 hours under an argon atmosphere (monitored by TLC). After the completion of the reaction (monitored by TLC), excess POCl_3_ was removed under vacuum, and the reaction mixture was quenched with saturated NaHCO_3_ solution and extracted with EtOAc (10 mL × 3). The organic layer was washed with brine, dried with anhydrous Na_2_SO_4_, and concentrated. The obtained crude product was purified by reverse-phase prep-purification to give corresponding LND-1,3,4-oxadiazole molecules 7(a–h).

##### 5.1.3.1 2-(1-(2,4-Dichlorobenzyl)-1*H*-indazol-3-yl)-5-phenyl-1,3,4-oxadiazole (7a)


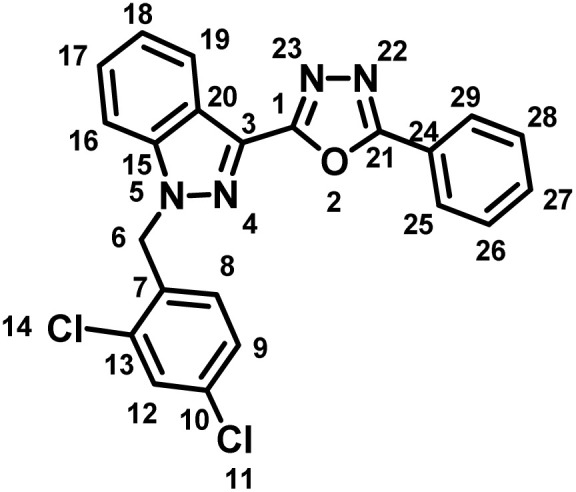
(2,4-Dichlorobenzyl)-1*H*-indazole-3-carboxylic acid (lonidamine) (5) and benzohydrazide (6a) afforded the pure title product as white solid. Yield; 32%; m.p 191–193 °C; ^1^H NMR (400 MHz, DMSO-*d*_6_) *δ* = 8.33 (d, *J* = 8.2 Hz, 1H, indazole-H-16, 19), 8.17–8.09 (m, 2H), 7.91 (d, *J* = 8.7 Hz, 1H), 7.73–7.59 (m, 5H), 7.48 (t, *J* = 7.2 Hz, 1H), 7.40 (dd, *J* = 2.2, 8.4 Hz, 1H), 7.03 (d, *J* = 8.4 Hz, 1H, H-8), 5.95 (s, 2H, –CH_2_–, H-6). ^13^C NMR (101 MHz, DMSO-*d*_6_) *δ* = 163.46, 159.60, 140.91, 133.45, 133.24, 133.19, 132.14, 130.93, 129.62, 129.52, 129.13, 127.94, 127.83, 126.77, 123.51, 123.15, 121.51, 121.12, 110.91, 49.90. MS-ESI (*m*/*z*): 420.90 (M + 1)^+^; HPLC: 92.19%; RT: 5.942 min.

##### 5.1.3.2 2-(1-(2,4-Dichlorobenzyl)-1*H*-indazol-3-yl)-5-(*p*-tolyl)-1,3,4-oxadiazole (7b)


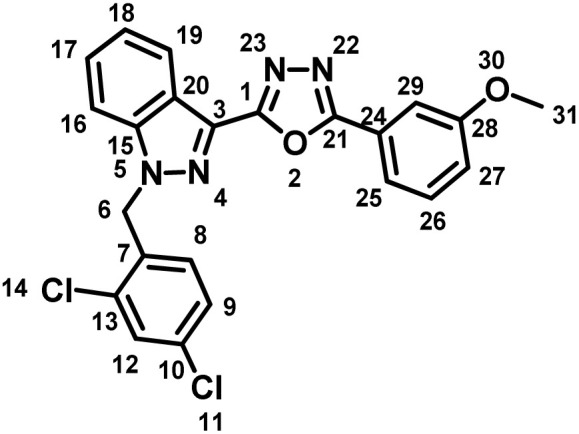
(2,4-Dichlorobenzyl)-1*H*-indazole-3-carboxylic acid (5) and 4-methylbenzohydrazide (6b) afforded the pure title product as an off-white solid. Yield: 43%; m.p.186–187 °C; ^1^H NMR (400 MHz, DMSO-*d*_6_) *δ* = 8.32 (d, *J* = 8.1 Hz, 1H, indazole-H-16, 19), 8.01 (d, *J* = 8.2 Hz, 2H), 7.90 (d, *J* = 8.7 Hz, 1H), 7.72 (d, *J* = 2.2 Hz, 1H), 7.60 (t, *J* = 7.7 Hz, 1H), 7.49–7.43 (m, 3H), 7.40 (dd, *J* = 2.2, 8.4 Hz, 1H), 7.02 (d, *J* = 8.4 Hz, 1H, H-8), 5.94 (s, 2H, –CH_2_–, H-6), 2.45–2.40 (m, 3H, –CH_3_, H-30). ^13^C NMR (101 MHz, DMSO-*d*_6_) *δ* = 164.04, 159.85, 142.83, 141.40, 133.94, 133.70, 131.38, 130.55, 130.17, 129.62, 128.41, 128.33, 127.22, 123.96, 121.98, 121.62, 120.88, 111.37, 50.38, 21.67. MS-ESI (*m*/*z*): 435.0 (M + 1)^+^; HPLC: 98.28%; RT: 6.076 min.

##### 5.1.3.3 2-(1-(2,4-Dichlorobenzyl)-1*H*-indazol-3-yl)-5-(3-methoxyphenyl)-1,3,4-oxadiazole (7c)


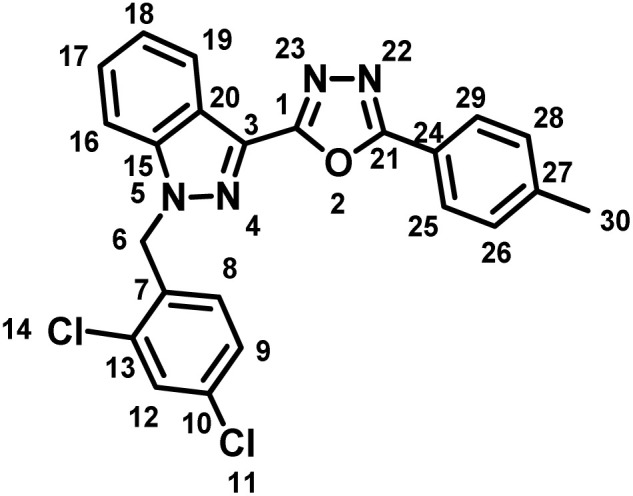
(2,4-Dichlorobenzyl)-1*H*-indazole-3-carboxylic acid (5) and 3-methoxybenzohydrazide (6c) afforded the pure title product as an off white solid. Yield: 63%; m.p.176–178 °C; ^1^H NMR (400 MHz, DMSO-*d*_6_) *δ* = 8.18 (d, *J* = 8.2 Hz, 1H, indazole-H-16, 19), 7.98–7.86 (m, 3H), 7.72 (d, *J* = 2.1 Hz, 1H), 7.70–7.63 (m, 2H), 7.58 (t, *J* = 7.6 Hz, 1H), 7.50–7.36 (m, 2H), 7.08 (d, *J* = 8.4 Hz, 1H, H-8), 5.95 (s, 2H, –CH_2_–, H-6), 3.88 (s, 3H, –OCH_3_, H-31). ^13^C NMR (101 MHz, DMSO-*d*_6_) *δ* = 165.42, 160.03, 150.21, 141.02, 133.81, 133.57, 133.37, 132.91, 131.22, 131.15, 130.23, 129.14, 127.82, 127.50, 127.04, 126.82, 123.95, 123.16, 122.65, 121.33, 111.08, 52.42, 50.11. MS-ESI (*m*/*z*): 452.0 (M + 2)^+^; HPLC: 98.33%; RT: 6.694 min.

##### 5.1.3.4 2-(4-Chlorophenyl)-5-(1-(2,4-dichlorobenzyl)-1*H*-indazol-3-yl)-1,3,4-oxadiazole (7d)


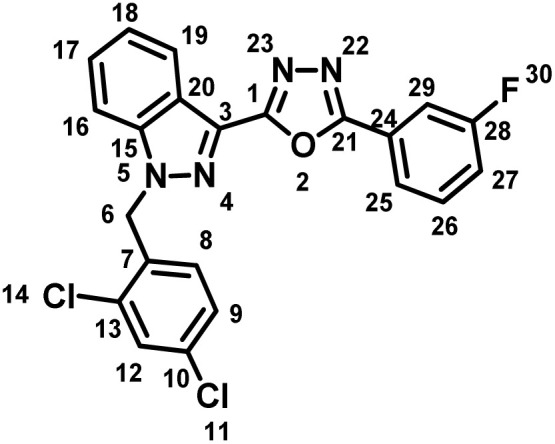
(2,4-Dichlorobenzyl)-1*H*-indazole-3-carboxylic acid (5) and 3-methoxybenzohydrazide (6d) afforded the pure title product as an off white solid. Yield: 51%; m.p.167–168 °C; ^1^H NMR (400 MHz, DMSO-*d*_6_) *δ* = 8.32 (d, *J* = 8.2 Hz, 1H, indazole-H-16, 19), 8.13 (d, *J* = 8.7 Hz, 2H), 7.91 (d, *J* = 8.7 Hz, 1H), 7.76–7.67 (m, 3H), 7.61 (t, *J* = 7.5 Hz, 1H), 7.50–7.43 (m, 1H), 7.40 (dd, *J* = 2.1, 8.4 Hz, 1H), 7.04 (d, *J* = 8.4 Hz, 1H, H-8), 5.94 (s, 2H, –CH_2_–, H-6). ^13^C NMR (101 MHz, DMSO-*d*_6_) *δ* = 162.73, 159.73, 140.91, 136.83, 133.48, 133.27, 133.15, 130.99, 129.67, 129.51, 129.14, 128.59, 127.96, 127.83, 123.55, 122.06, 121.50, 121.09, 110.94, 49.92. MS-ESI (*m*/*z*): 454.85 (M + 1)^+^; HPLC: 94.89%; RT: 8.585 min.

##### 5.1.3.5 2-(1-(2,4-Dichlorobenzyl)-1*H*-indazol-3-yl)-5-(3-fluorophenyl)-1,3,4-oxadiazole (7e)


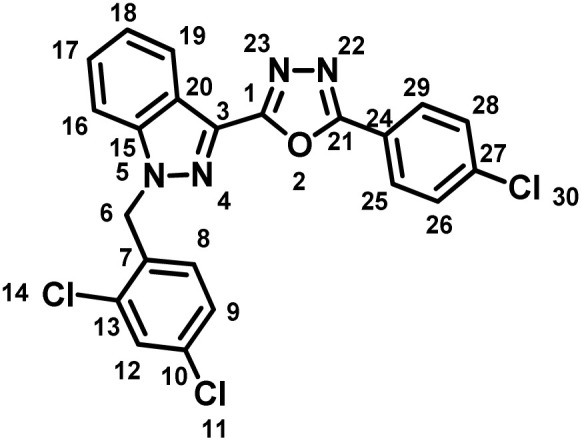
(2,4-Dichlorobenzyl)-1*H*-indazole-3-carboxylic acid (5) and 3-methoxybenzohydrazide (6e) afforded the pure title product as an off-white solid. Yield: 44%; m.p.162–164 °C; ^1^H NMR (400 MHz, DMSO-*d*_6_) *δ* = 8.34 (d, *J* = 8.2 Hz, 1H, indazole-H-16, 19), 8.01–7.85 (m, 2H), 7.76–7.68 (m, 2H), 7.62 (br t, *J* = 7.8 Hz, 1H), 7.57–7.32 (m, 4H), 7.03 (d, *J* = 8.5 Hz, 1H, H-8), 5.95 (s, 2H, –CH_2_–, H-6); ^19^F NMR (376 MHz, DMSO-*d*_6_) *δ* = −111.23 (s, 1F); ^13^C NMR (101 MHz, DMSO-*d*_6_) *δ* = 183.61, 161.22 (d, *J* = 273.2 Hz, 1C), 140.92, 133.48, 133.25, 133.15, 131.94 (br d, *J* = 8.0 Hz, 1C), 130.94, 129.47, 129.14, 127.91 (br d, *J* = 13.1 Hz, 1C), 127.19, 125.69 (br d, *J* = 90.8 Hz, 1C), 123.59, 123.12 (d, *J* = 2.9 Hz, 1C), 121.53, 121.13, 119.24, 119.03, 113.51 (br d, *J* = 23.3 Hz, 1C), 110.94, 49.94. MS-ESI (*m*/*z*): 438.7 (M + 1)^+^; HPLC: RT: 96.09%; RT: 5.881 min.

##### 5.1.3.6 2-(1-(2,4-Dichlorobenzyl)-1*H*-indazol-3-yl)-5-(4-(trifluoromethyl)phenyl)-1,3,4-oxadiazole (7f)


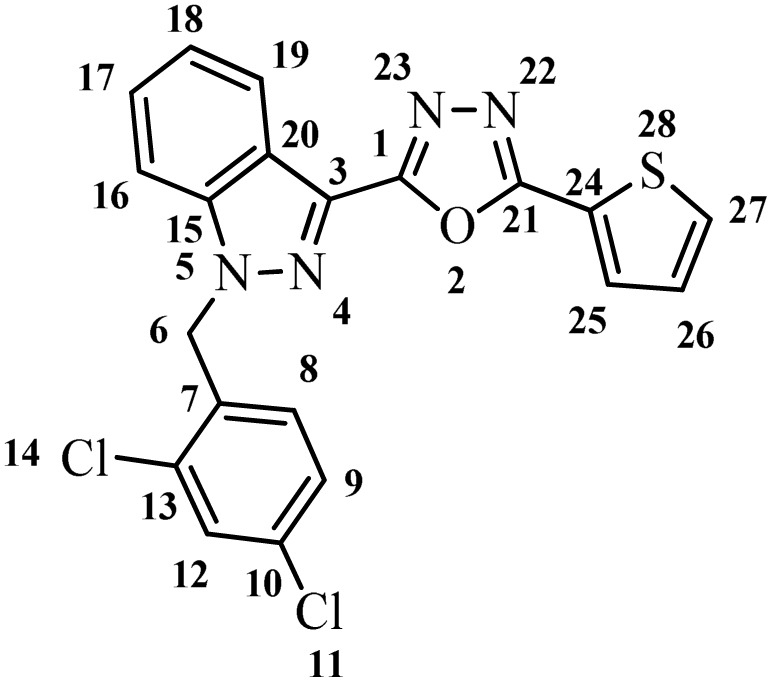
(2,4-Dichlorobenzyl)-1*H*-indazole-3-carboxylic acid and 4-trifluoromethyl benzohydrazide (6f) afforded the pure title product a white solid. Yield: 28%; m.p.196–198 °C; ^1^H NMR (400 MHz, DMSO-*d*_6_) *δ* = 8.33 (d, *J* = 8.2 Hz, 3H), 8.01 (d, *J* = 8.4 Hz, 2H), 7.93 (d, *J* = 8.7 Hz, 1H), 7.73 (d, *J* = 2.2 Hz, 1H), 7.62 (t, *J* = 7.8 Hz, 1H), 7.48 (t, *J* = 7.5 Hz, 1H), 7.41 (dd, *J* = 2.2, 8.4 Hz, 1H), 7.05 (d, *J* = 8.4 Hz, 1H, H-8), 5.96 (s, 2H, –CH_2_–, H-6), ^19^F NMR (376 MHz, DMSO-*d*_6_) *δ* = −61.59 (s, 1F)·^13^C NMR (101 MHz, DMSO-*d*_6_) *δ* = 162.46, 160.09, 140.94, 133.49, 133.28, 133.14, 131.74, 131.43, 130.99, 129.43, 129.15, 127.91 (br d, *J* = 16.0 Hz, 1C), 127.66, 126.99, 126.45 (br d, *J* = 3.6 Hz, 1C), 123.62, 121.55, 121.08, 110.98, 49.96. MS-ESI (*m*/*z*): 488.9 (M + 1)^+^; HPLC: 88%; RT: 6.177 min.

##### 5.1.3.7 2-(1-(2,4-Dichlorobenzyl)-1*H*-indazol-3-yl)-5-(thiophen-2-yl)-1,3,4-oxadiazole (7g)


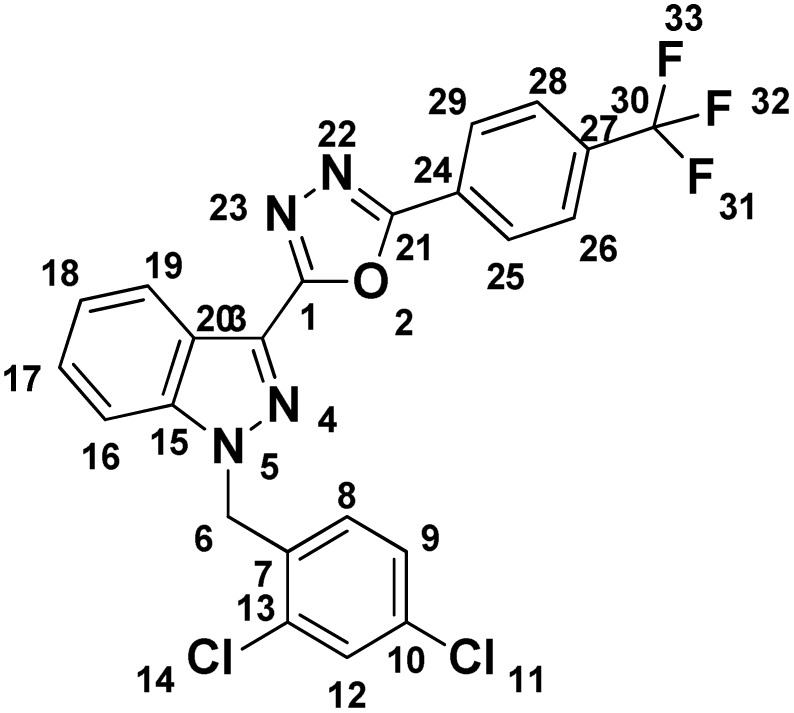
(2,4-Dichlorobenzyl)-1*H*-indazole-3-carboxylic acid (5) and thiophene 2-carbohydrazide (6g) afforded the pure title product a white solid. Yield: 35%; m.p.173–174 °C; ^1^H NMR (400 MHz, DMSO-*d*_6_) *δ* = 8.30 (d, *J* = 8.1 Hz, 1H), 8.00 (dd, *J* = 1.2, 5.0 Hz, 1H), 7.96 (dd, *J* = 1.2, 3.7 Hz, 1H), 7.90 (d, *J* = 8.5 Hz, 1H), 7.72 (d, *J* = 2.1 Hz, 1H), 7.61 (ddd, *J* = 1.0, 7.1, 8.4 Hz, 1H), 7.46 (t, *J* = 7.3 Hz, 1H), 7.39 (dd, *J* = 2.2, 8.4 Hz, 1H), 7.33 (dd, *J* = 3.7, 4.9 Hz, 1H), 7.01 (d, *J* = 8.4 Hz, 1H, H-8), 5.95 (s, 2H, –CH_2_–, H-6); ^13^C NMR (101 MHz, DMSO-*d*_6_) *δ* = 159.84, 159.00, 140.93, 133.48, 133.25, 133.20, 130.91, 130.73, 129.45, 129.16, 128.94, 128.00, 127.85, 124.04, 123.57, 121.52, 121.13, 110.94, 104.42, 49.94. MS-ESI (*m*/*z*): 426.85 (M + 1)^+^; HPLC: 94.9%; RT: 6.728 min.

##### 5.1.3.8 2-Cyclopentyl-5-(1-(2,4-dichlorobenzyl)-1*H*-indazol-3-yl)-1,3,4-oxadiazole (7h)


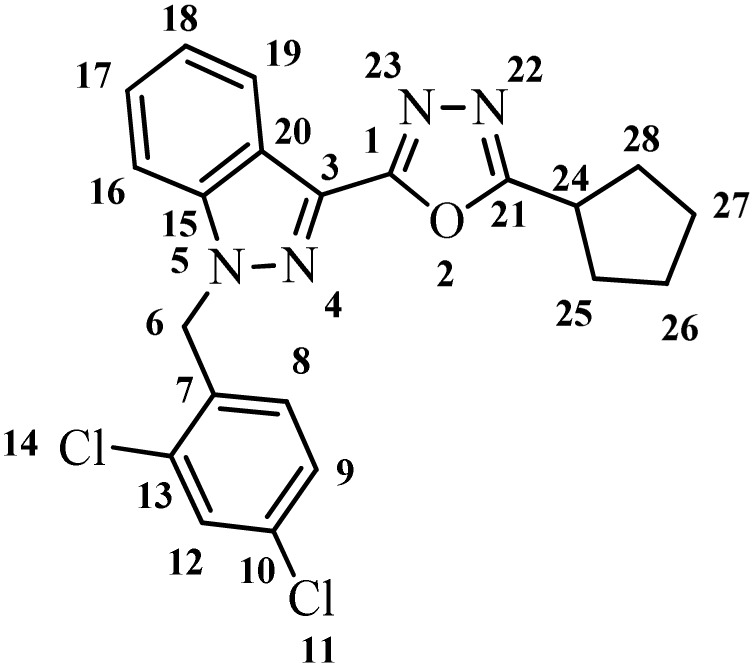
(2,4-Dichlorobenzyl)-1*H*-indazole-3-carboxylic acid (5) and cyclopentane carbohydrazide (6h) afforded the pure title product a white solid. Yield: 37%; m.p.180–182 °C; ^1^H NMR (400 MHz, DMSO-*d*_6_) *δ* = 8.22 (d, *J* = 8.2 Hz, 1H), 7.87 (d, *J* = 8.7 Hz, 1H), 7.75–7.67 (m, 1H), 7.63–7.53 (m, 1H), 7.48–7.36 (m, 2H), 6.98 (d, *J* = 8.4 Hz, 1H, H-8), 5.90 (s, 2H, –CH_2_–, H-6), 3.56–3.39 (m, 1H, –CH–, H-24), 2.16–2.07 (m, 2H, –CH_2_–, H-25), 2.02–1.85 (m, 2H, –CH_2_–, H-28), 1.80–1.63 (m, 4H, –CH_2_–, H-26, 27). ^13^C NMR (101 MHz, DMSO-*d*_6_) *δ* = 169.26, 159.42, 140.85, 133.42, 133.22, 130.90, 129.84, 129.11, 127.84, 127.84, 123.32, 121.31, 121.05, 110.82, 49.80, 35.12, 30.55, 25.07. MS-ESI (*m*/*z*): 412.8 (M + 1)^+^; HPLC: 92.73%; RT: 6.964 min.

### 5.2 Biological activity

#### 5.2.1 Cell lines and culture conditions

The cancer cell lines were purchased from National Centre of Cell Science (NCCS, Pune) and authenticated with STR analysis and the cells were tested for mycoplasma contaminations. Cells were cultured in IMDM and DMEM, respectively, with 2 mM l-glutamine (Thermo Fisher Scientific, Inc.; Waltham, MA, USA) containing 10% FBS (Gibco; Grand Island, NY, USA). Cells were cultured in a humidified incubator with 5% CO_2_ at 37 °C.

#### 5.2.2 MTT and trypan blue dye exclusion assay

MTT and trypan blue dye exclusion assays were performed as described in ref. 41. Briefly, cells treated with different concentrations of compound (7d) (1, 10, 25, and 50 µM) were incubated and subjected to MTT and trypan blue dye assays with DMSO serving as a negative control. The plates were subjected to spectrophotometric absorbance at 570 nm using a Tecan Microplate Reader (Tecan Instruments, Switzerland). The trypan blue-treated cells were counted using the Thermo Fisher Countess III FL for calculating the percentage of live and dead cells. The data were analyzed in Microsoft Excel.

#### 5.2.3 Selectivity index

The selectivity index (SI) was determined by comparing the cytotoxicity of the small molecule in normal cells (Hek) with its inhibitory activity against colon carcinoma cells (HCT116). Cells were seeded in 96-well plates and treated with increasing concentrations of the compound for 48 h under standard culture conditions. Cell viability was measured using the MTT assay, and CC_50_ and IC_50_ values were calculated from nonlinear dose–response curves. The SI was calculated as the ratio of CC_50_ to IC_50_. All experiments were performed in triplicate, and results were expressed as mean ± SD.

#### 5.2.4 Apoptotic analysis by Hoechst/PI staining

The Hoechst/PI double staining assay was conducted following the protocol described in ref. 42. Briefly, HCT116 cells were seeded at a density of 1 × 10^5^ cells per well in 6-well plates and allowed to attach overnight. Subsequently, they were treated with increasing concentrations of compound (7d) and incubated for 48 hours before undergoing Hoechst/PI double staining.

#### 5.2.5 Apoptotic analysis by acridine orange/PI dual staining

HCT116 cells were seeded, incubated overnight, and then treated with increasing concentrations of compound (7d) and allowed to incubate for 48 h. Cells were collected, washed with PBS, and subjected to apoptotic analysis by acridine orange/PI double staining and imaged.

#### 5.2.6 Migration assay/wound healing assay

HCT116 cells were seeded at a density of 2 × 10^5^ cells per 12-well plate and left to attach overnight. The cells were allowed to grow until they reached complete confluency. Subsequently, a scar was created using a sterile tip, and the cells were treated with increasing concentrations of compound (7d). After 24 h of incubation, the cells were imaged to assess the extent of scar/wound recovery.

### 5.3 Computational studies

#### 5.3.1 Molecular docking

The protein structure of MMP2 (PDB ID: 7XJO) was obtained, with crystallographic water molecules and other ions removed. The protein was prepared by adding polar hydrogens and assigning Kollman charges using AutoDock Tools. To predict the active site, a preliminary “blink docking” was performed to identify the potential binding site. Following this, specific docking calculations were conducted within a predefined grid box was created to encompass the active site residues, ensuring full coverage of the catalytic pocket. Ligands, including compounds 7(a–h) and the reference ligand lonidamine, were generated, energy-minimized, and converted into the PDBQT format using OpenBabel. Molecular docking was carried out using AutoDock Vina 1.2.2,^43^ with docking runs automated *via* the batch script “Execute.bat”.^44^ The best-ranked pose for each ligand was selected based on binding energy, along with visual inspection of hydrogen bonding, π–π stacking, halogen interactions, and hydrophobic contacts.

#### 5.3.2 Molecular dynamics simulation

Molecular dynamics (MD) simulations were conducted to evaluate the microscopic stability and conformational behavior of the apoprotein and protein–ligand complexes. All simulations were performed using AMBER 22.^45^ The protein was parameterized using the FF19SB force field,^46^ while ligand parameters were generated using the General AMBER Force Field (GAFF2).^47^ Ligand atomic partial charges were assigned using the AM1-BCC method *via* the Antechamber module. Each system was solvated in an explicit TIP3P water model within a truncated octahedral box, maintaining a minimum distance of 10 Å between the solute and box boundaries. Appropriate numbers of Na^+^ and Cl^−^ ions were added to neutralize the system and achieve electrostatic stability. Energy minimization was performed in two stages to remove steric clashes and unfavorable contacts. Initially, solvent molecules and counterions were minimized while restraining the solute, followed by full system minimization without positional restraints. After minimization, systems were gradually heated from 0 K to 300 K under constant volume (NVT ensemble) for 100 ps while applying positional restraints to the protein backbone. Subsequently, a 100 ps equilibration under constant pressure (NPT ensemble) at 300 K and 1 atm was conducted to stabilize system density and pressure. Temperature was controlled using a Langevin thermostat, and pressure was regulated under periodic boundary conditions. Long-range electrostatic interactions were treated using the Particle Mesh Ewald (PME) method with a 10 Å cutoff for non-bonded interactions. All covalent bonds involving hydrogen atoms were constrained using the SHAKE algorithm, allowing a 2 fs integration time step. Following equilibration, a 500 ns production MD simulation was performed for each system at 300 K and 1 atm. Trajectories were recorded at defined intervals for subsequent structural and dynamic analyses, including RMSD, RMSF, radius of gyration (*R*_g_), principal component analysis (PCA) using cpptraj.^48^ The first two principal components were converted into the probability density and visualized as a free energy landscape using the FEL calculation package^49^ and crucial interval calculation package.^50^ Molecular Mechanics Generalized Born Surface Area (MM/GBSA) was given for the crucial interval at the basin of the FEL *via* MMPBSA.py package.^51^

## Author contributions

Rangappa Keri: resources, writing – original draft, project administration, funding acquisition, data curation, conceptualization methodology, funding acquisition. Raveendra Madhukar Bhat: data curation, conceptualization methodology, data curation. Priyadarshini A. N.: methodology, visualization, validation. Dr Sudhanva M. S.: visualization, resources, formal analysis, data curation. Gangadhar V Muddapur: software, methodology, formal analysis. Kawthar Alhussinei: software, methodology, formal analysis. Raman Kumar K: visualization.

## Conflicts of interest

The authors declared that they have no conflicts of interest.

## Abbreviations

ADMET: Absorption, distribution, metabolism, excretion, and toxicity
AO: Acridine orange
ANOVA: Analysis of variance
ADP: Adenosine diphosphate
BBB: Blood brain barrier
CNS: Central nervous system
CRC: Colorectal cancer
DFT: Density functional theory
DNA: Deoxyribonucleic acid
FBS: Fetal bovine serum
FDA: Food and drug administration
FEL: Free energy landscape
GCO: Global cancer observatory
h: Hour
HK-2: Hexokinase 2
HOMO: Highest occupied molecular orbital
HPLC: High-performance liquid chromatography
IARC: International agency for research on cancer
LND: Lonidamine
LUMO: Lowest unoccupied molecular orbital
IC_50_: Half-maximal inhibitory concentration
MEP: Molecular electrostatic potentials
µg: Micro gram
mL: Milli litre
m.p.: Melting points
MTT: 3-(4,5-Dimethylthiazol-2-yl)-2,5-diphenyltetrazolium bromide
M.W.: Molecular weight
NMR: Nuclear magnetic resonance
NSCLC: Non-small cell lung cancer
OLED: Organic light emitting diode
PCA: Principal component analysis
PDB: Protein data bank
PI: Propidium iodide
PROTAC: Proteolysis targeting chimeras
*R*_g_
: Radius of gyration
RMSD: Root mean square deviation
RMSF: Root mean square fluctuation
ROS: Reactive oxygen species
SAR: Structure activity relationship
SASA: Solvent accessible surface area
SEM: standard error of the mean
TLC: Thin layer chromatography
TMS: Tetramethylsilane
TPSA: Topological polar surface area
WHO: World health organization

## Supplementary Material

RA-016-D5RA07852K-s001

## Data Availability

All the data is contained in the manuscript. More data can be obtained from the corresponding author through request email. Supplementary information (SI) is available. See DOI: https://doi.org/10.1039/d5ra07852k.
