# Supplementary material for: Lonidamine-1,3,4-oxadiazole derivatives with antiproliferative effects on HCT116 colon cancer cell lines: biological evaluation, ADMET, and computational studies
Source: RSC Adv. 2026 Mar 11;16(15):13624–43. doi: 10.1039/d5ra07852k (PMC12977971; doi:10.1039/d5ra07852k)
Supplement: RA-016-D5RA07852K-s001 [file RA-016-D5RA07852K-s001.pdf]

Supplementary Information (SI) for RSC Advances.
This journal is © The Royal Society of Chemistry 2026

Supporting Information

Title: Lonidamine-1,3,4-oxadiazole derivatives with antiproliferative effects on HCT116 colon cancer cell lines: Biological evaluation, ADMET, toxicity and computational studies

Author: Raveendra Madhukar Bhat,[1,2] Priyadarshini A.N.,[3] Sudhanva M. S.[3], Gangadhar V. Muddapur,[4] Kawthar Alhussieni,[5] Raman Kumar K.,[2] Rangappa S. Keri[1]*

[1]Centre for Nano and Material Sciences, Jain (Deemed-to-be University), Jain Global Campus, Kanakapura, Bangalore, Karnataka, India – 562112

[2]Aurigene Pharmaceutical Services, Bangalore, Karnataka, India.

[3]Adichunchanagiri Institute for Molecular Medicine, Adichunchanagiri Institute of Medical Sciences, Adichunchanagiri University, BG Nagara-571448 Karnataka, India.

[4]Department of Physics, KLE Technological University, Hubballi-580031, Karnataka, India.

[5]Department of Pharmaceutical Chemistry, Faculty of Pharmacy, Universiti Malaya, Kuala Lumpur 50603, Malaysia

Contents

1. **Spectral copies of ^{1}H, ^{13}C, ^{19}F NMR and LC-MS of compounds 7(a-h)**
2. **Molecular Docking results of compounds 7(a-h)**
3. **Bioavailability radar chart of all derivatives from the Swiss ADME web server**
4. **HPLC data**
5. **DFT Studies**
6. **Video of molecular simulation study**

1. Spectral copies of ^{1}H, ^{13}C, ^{19}F NMR and LC-MS of compounds 7(a-h)

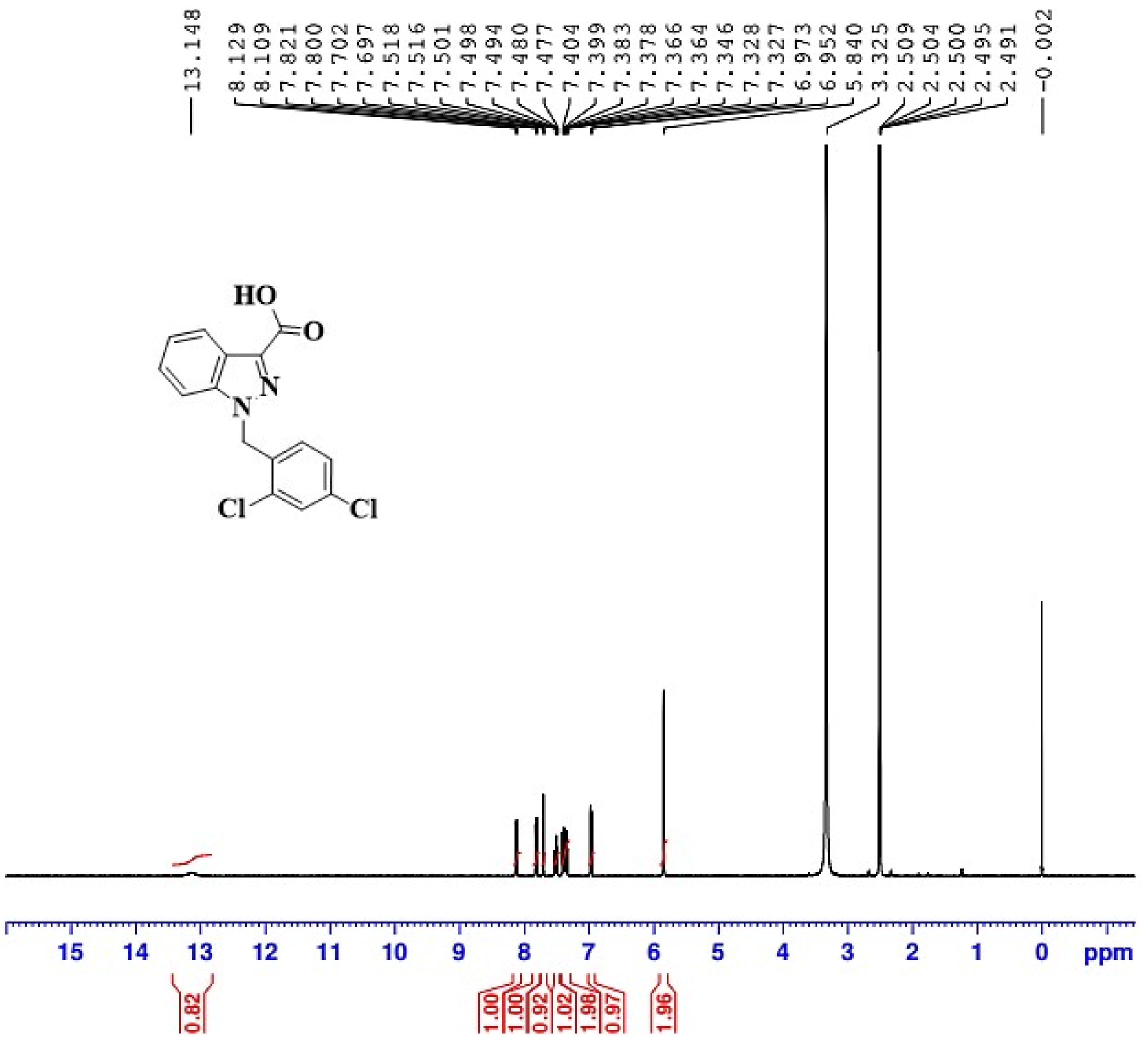

Fig S1: ^{1}HNMR spectra of 1-(2,4-dichlorobenzyl)-1*H*-indazole-3-carboxylic acid **(Lonidamine)** (**5**)

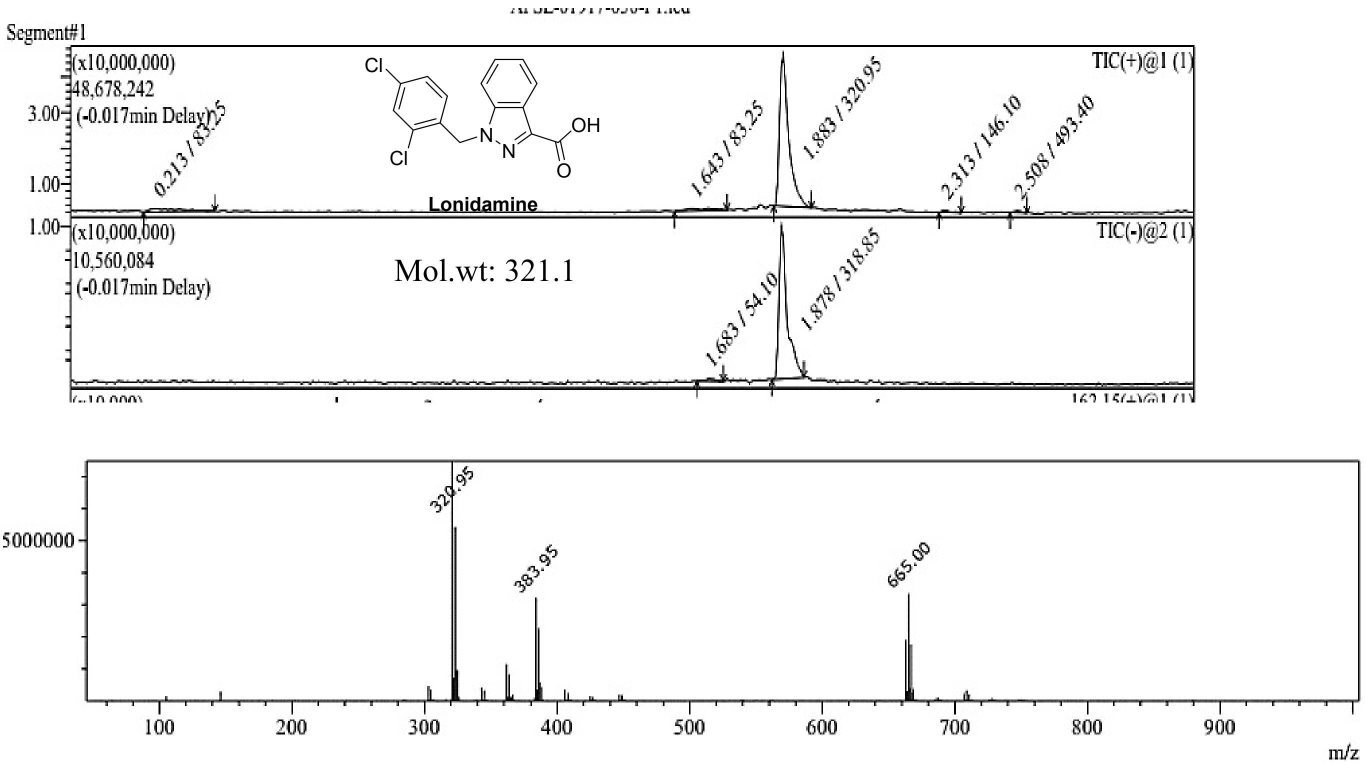

Fig S2: LCMS of 1-(2, 4-dichlorobenzyl)-1*H*-indazole-3-carboxylic acid **(Lonidamine)** (**5**)

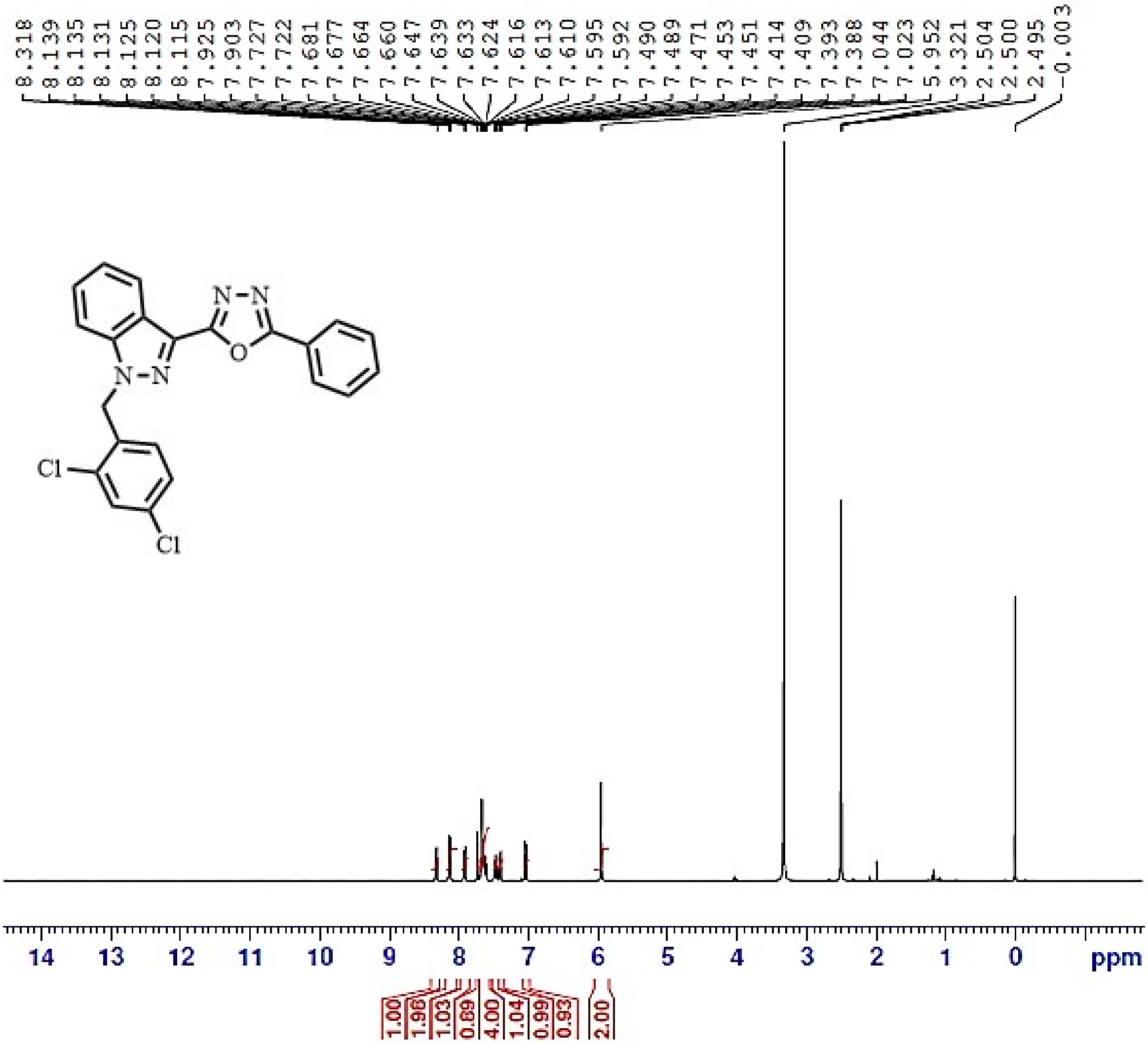

Fig S3: ^{1}HNMR spectra of 2-(1-(2,4-dichlorobenzyl)-1*H*-indazol-3-yl)-5-phenyl-1,3,4-oxadiazole **(7a)**

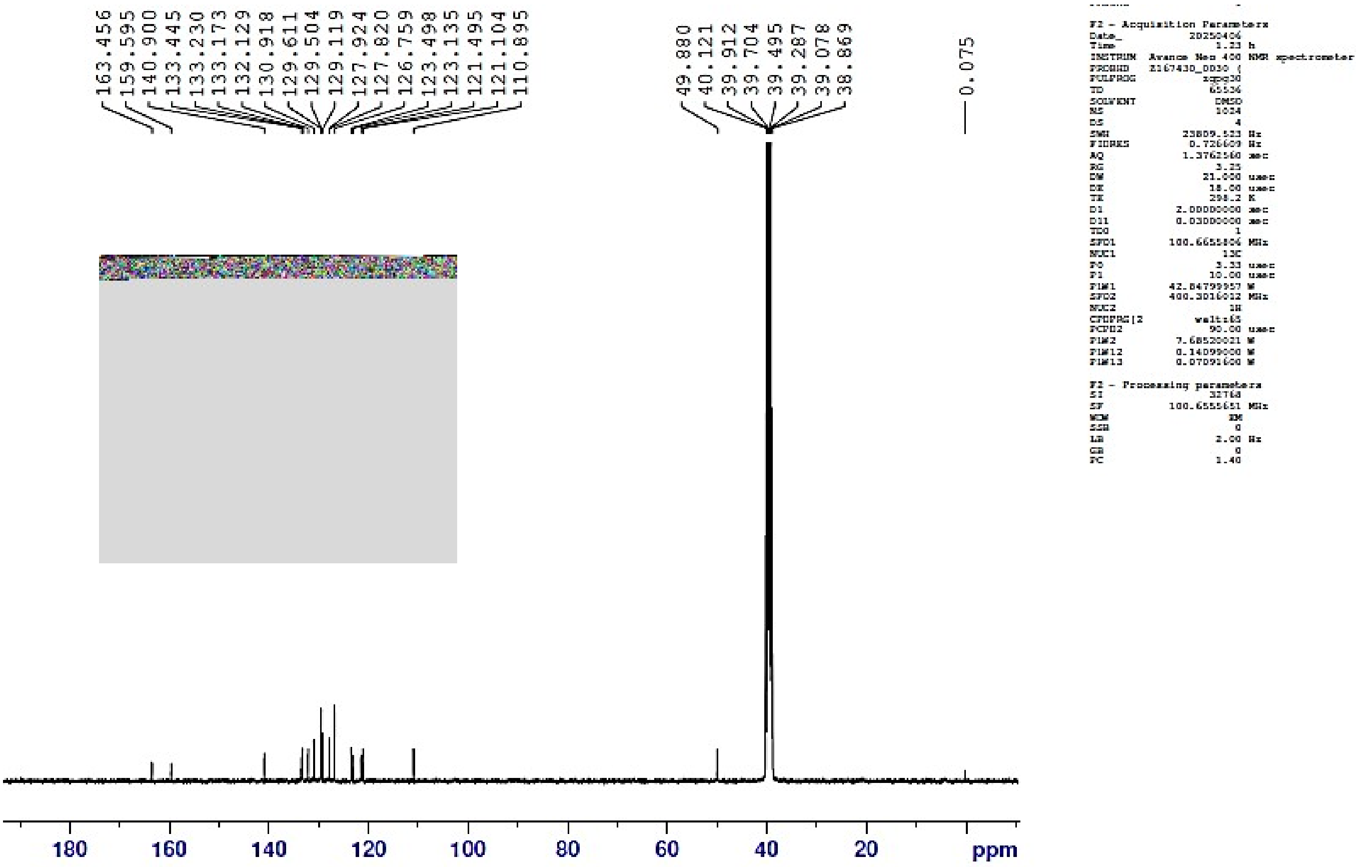

Fig S4: ^{13}C NMR spectra of 2-(1-(2,4-dichlorobenzyl)-1*H*-indazol-3-yl)-5-phenyl-1,3,4-oxadiazole **(7a)**

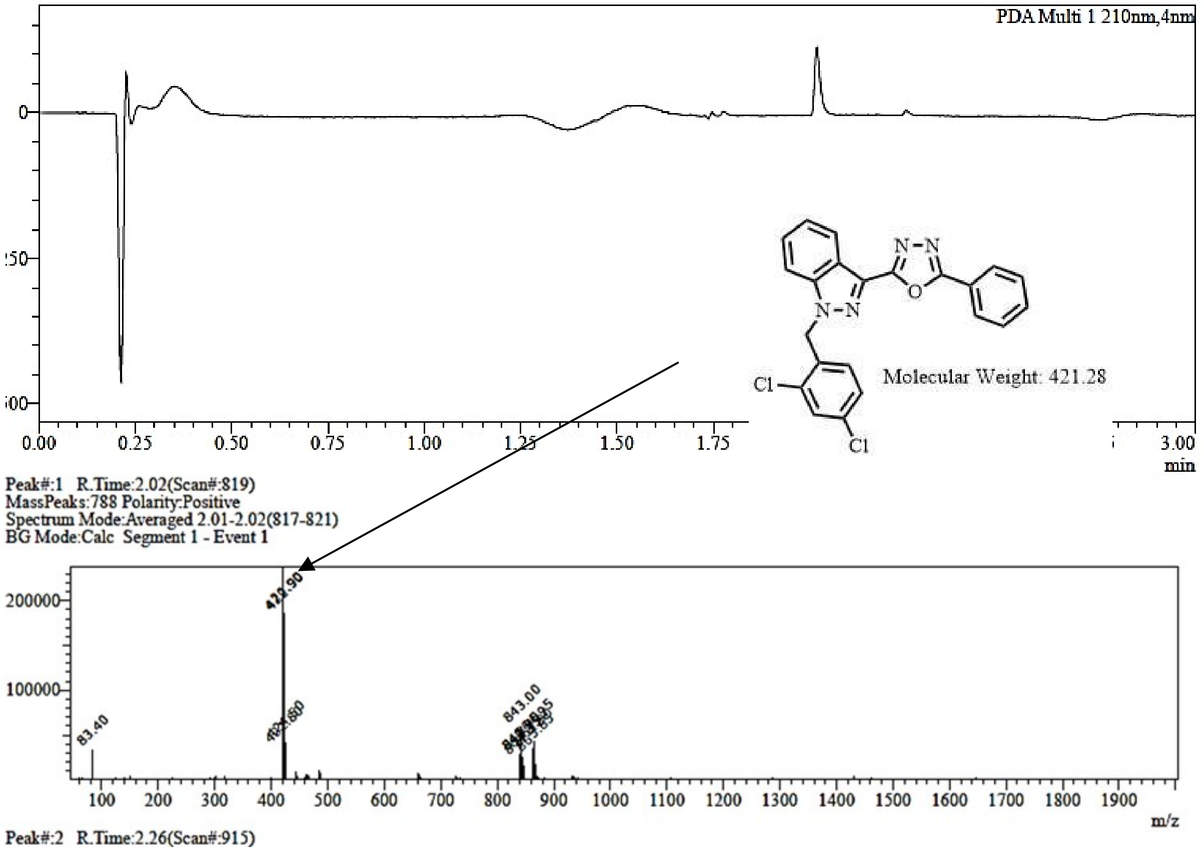

Fig S5: LCMS of 2-(1-(2,4-dichlorobenzyl)-1*H*-indazol-3-yl)-5-phenyl-1,3,4-oxadiazole **(7a)**

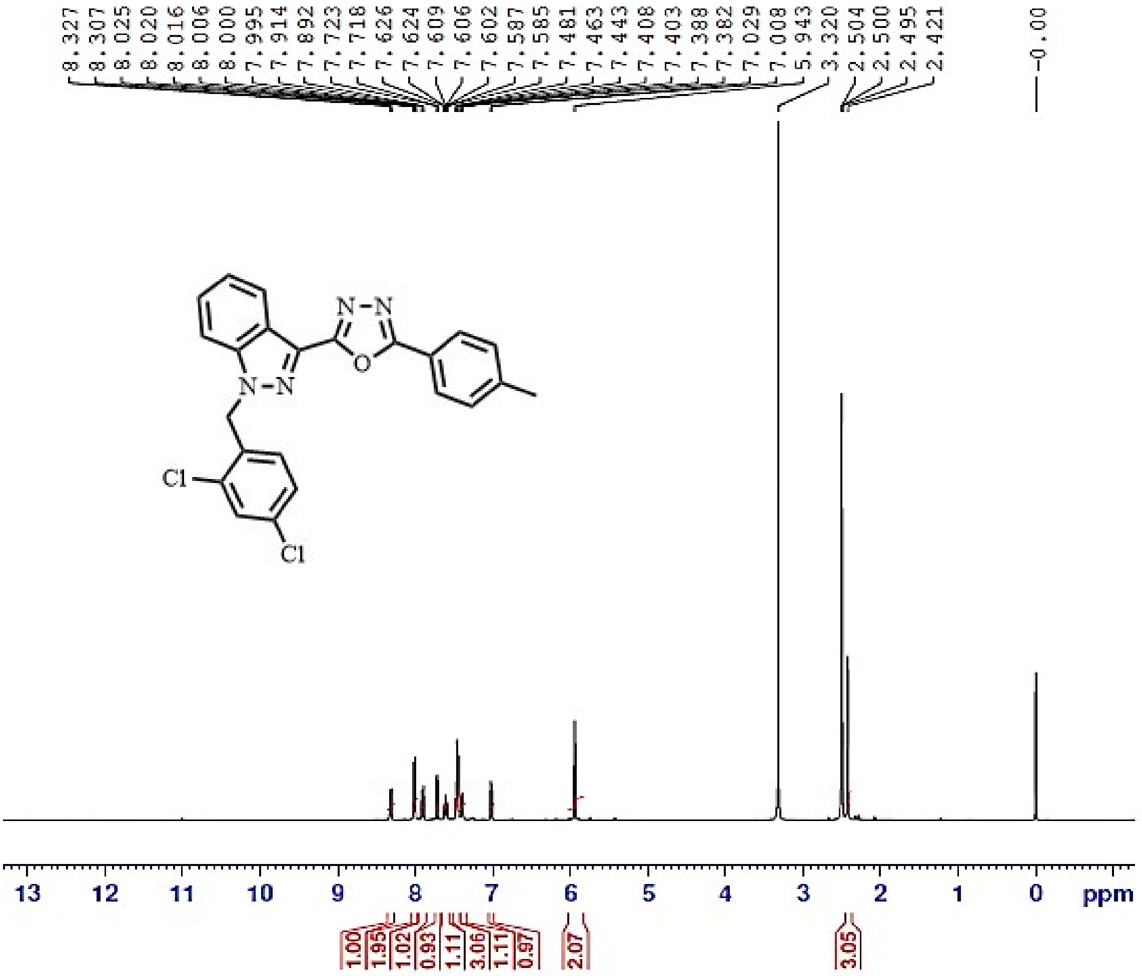

Fig S6: ^{1}H NMR spectra 2-(1-(2,4-dichlorobenzyl)-1*H*-indazol-3-yl)-5-(p-tolyl)-1,3,4-oxadiazole **(7b)**

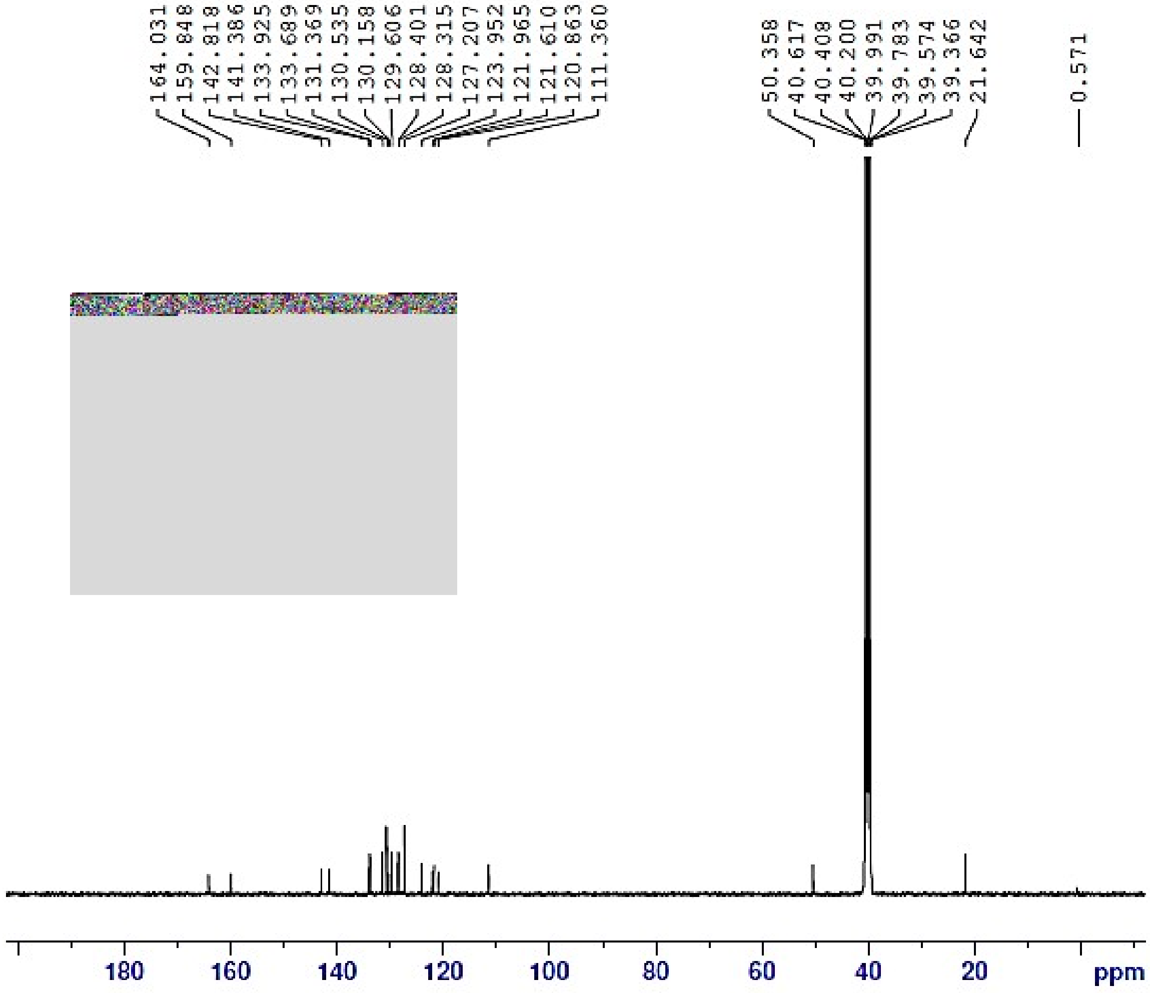

Fig S7: ^{13}C NMR spectra of 2-(1-(2,4-dichlorobenzyl)-1*H*-indazol-3-yl)-5-(p-tolyl)-1,3,4-oxadiazole **(7b)**

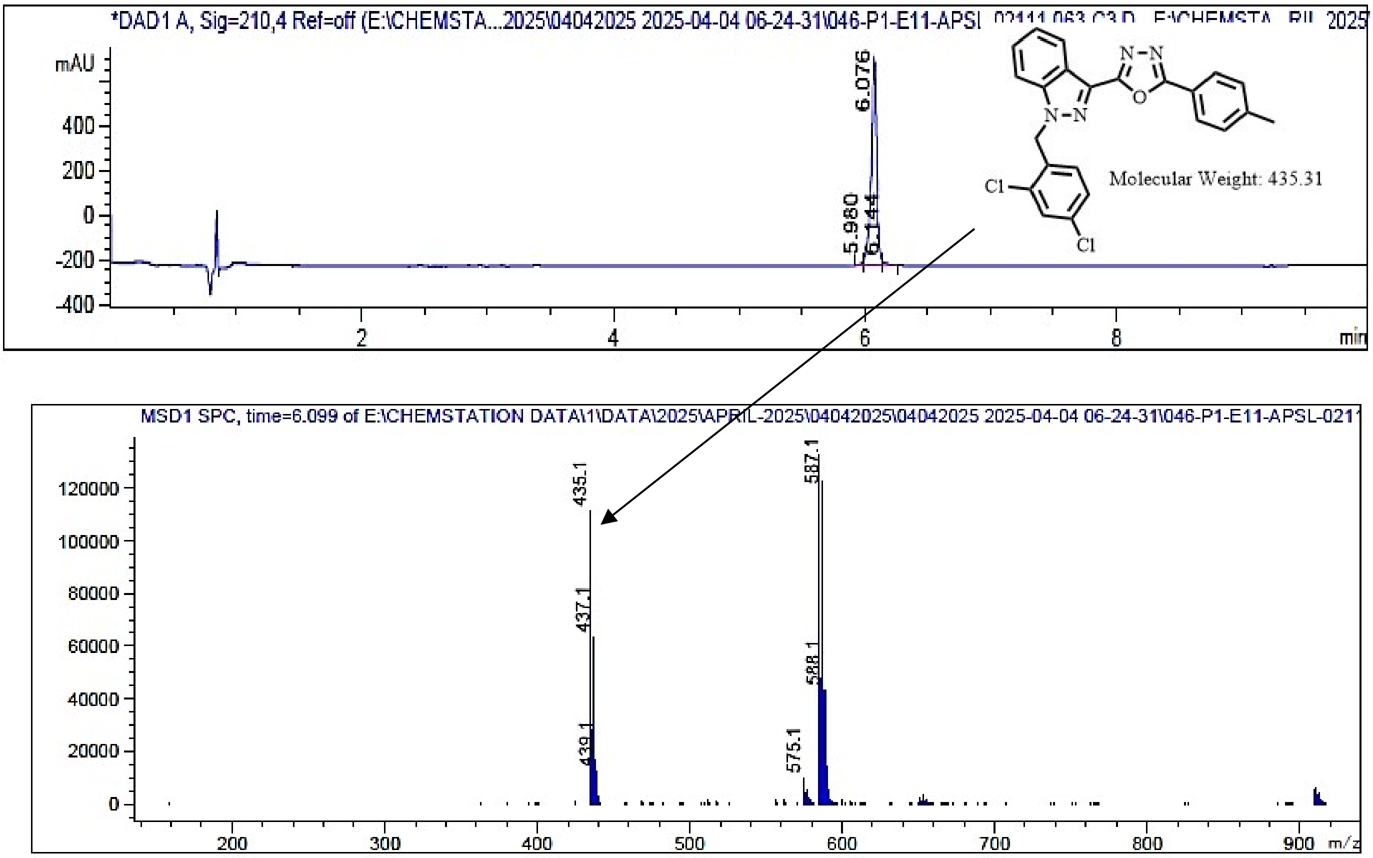

Fig S8: LCMS of 2-(1-(2,4-dichlorobenzyl)-1*H*-indazol-3-yl)-5-(p-tolyl)-1,3,4-oxadiazole **(7b)**

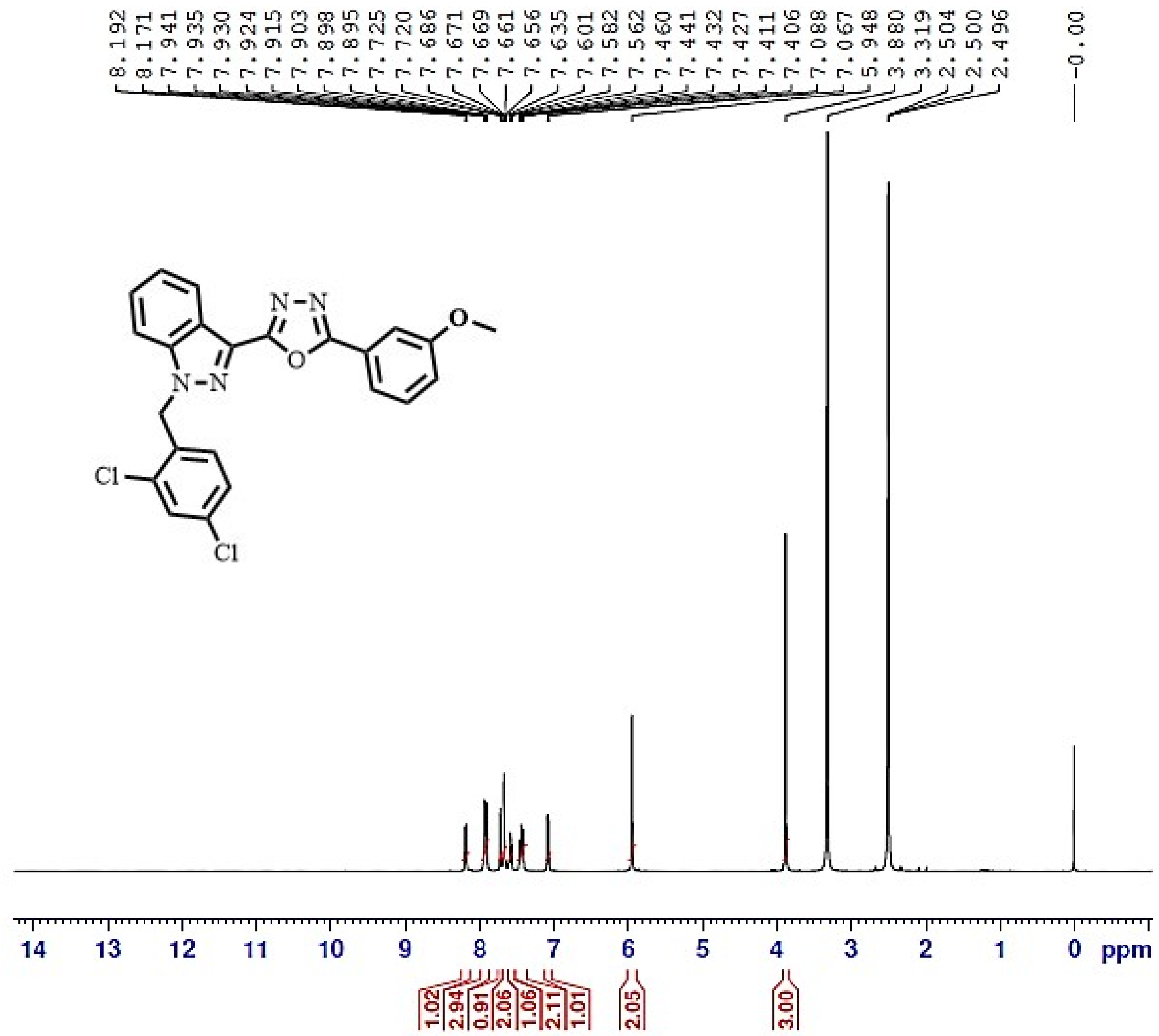

Fig S9: ^{1}H NMR spectra of 2-(1-(2,4-dichlorobenzyl)-1*H*-indazol-3-yl)-5-(3-methoxyphenyl)-1,3,4-oxadiazole **(7c)**

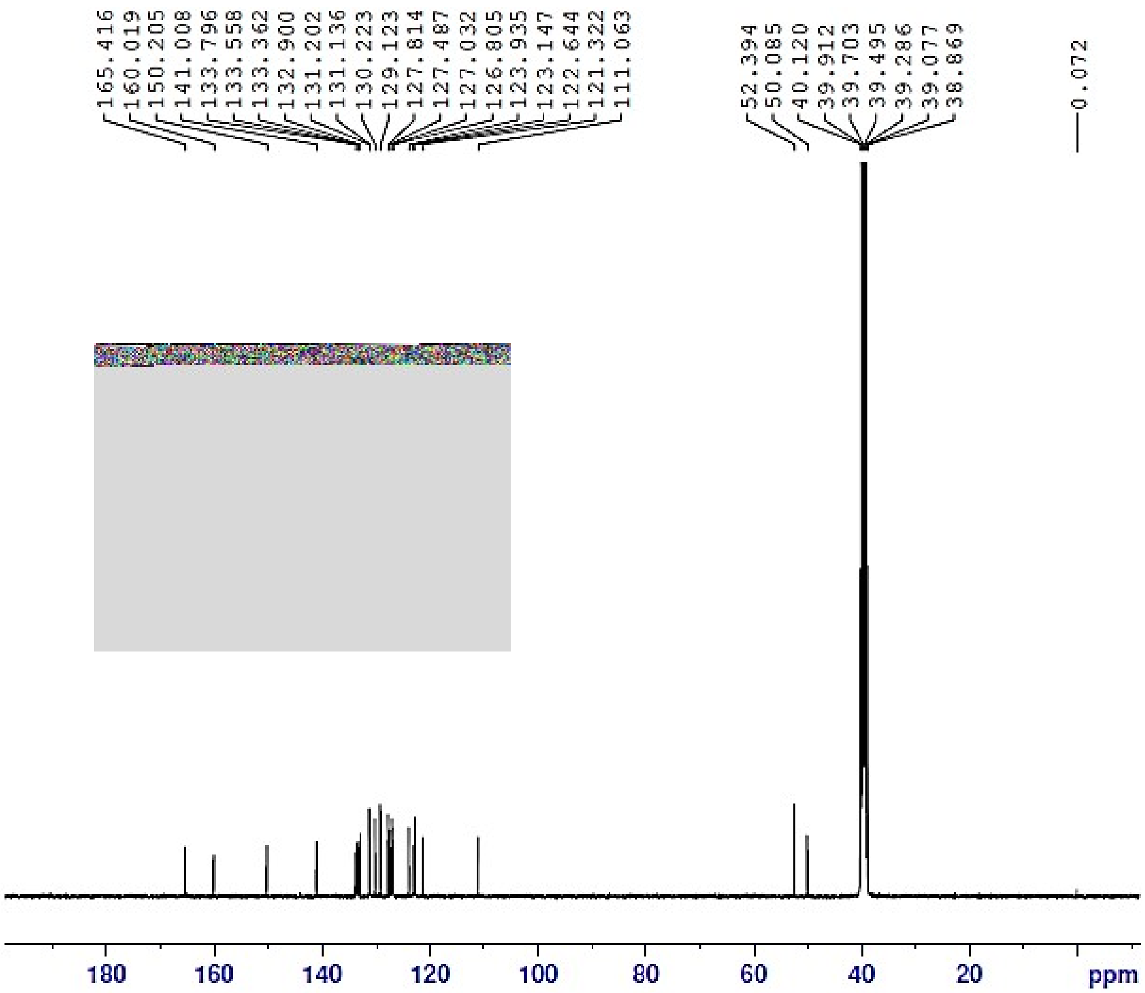

Fig S10: ^{13}CNMR spectra of 2-(1-(2,4-dichlorobenzyl)-1*H*-indazol-3-yl)-5-(3-methoxyphenyl)-1,3,4-oxadiazole **(7c)**

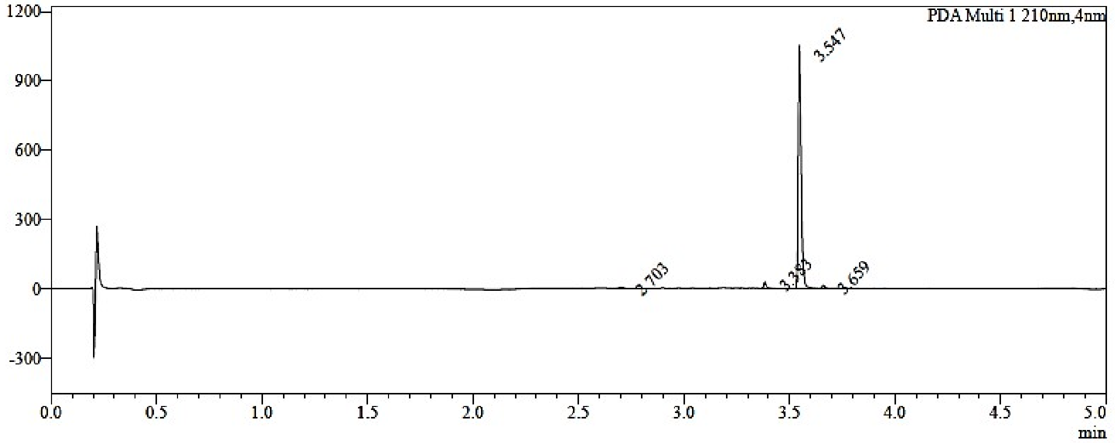

Peak Table
PDA Ch1 210nm

Peak#	Ret. Time	Area	Area%
1	2.703	11801	1.077
2	3.383	23093	2.108
3	3.547	1049280	95.795
4	3.659	11161	1.019
Total		1095336	100.000

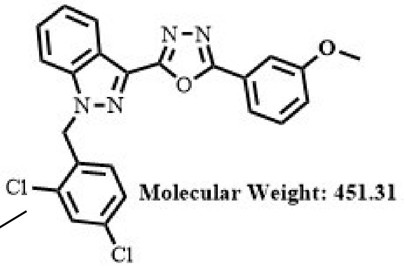

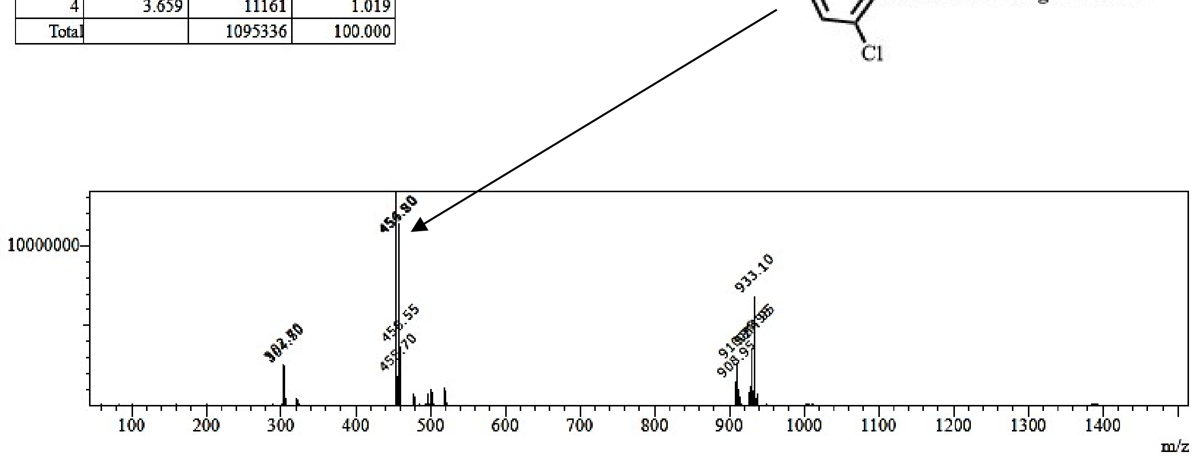

Fig S11: LCMS of 2-(1-(2,4-dichlorobenzyl)-1*H*-indazol-3-yl)-5-(3-methoxyphenyl)-1,3,4-oxadiazole **(7c)**

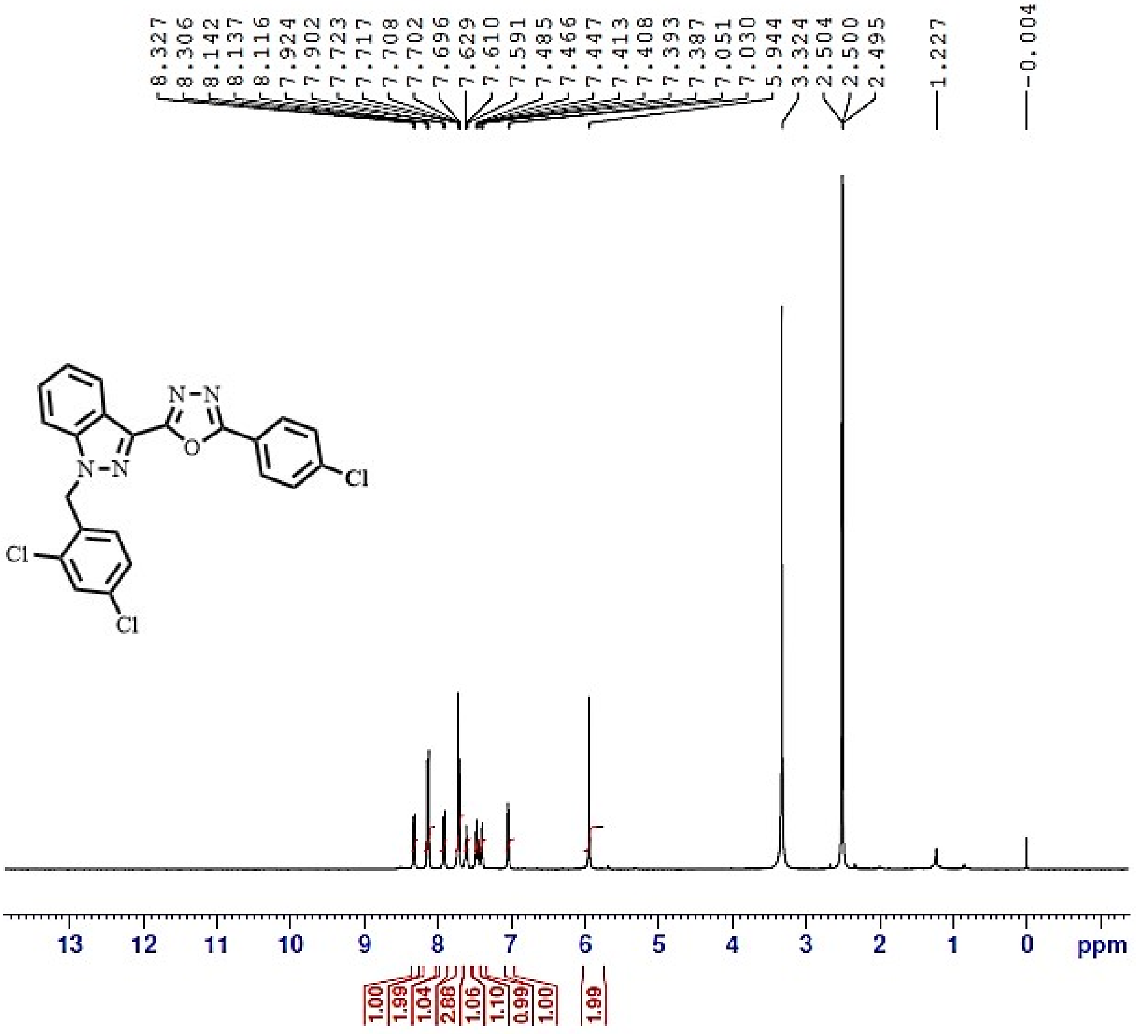

Fig S12: ^{1}HNMR spectra of 2-(4-chlorophenyl)-5-(1-(2,4-dichlorobenzyl)-1*H*-indazol-3-yl)-1,3,4-oxadiazole **(7d)**

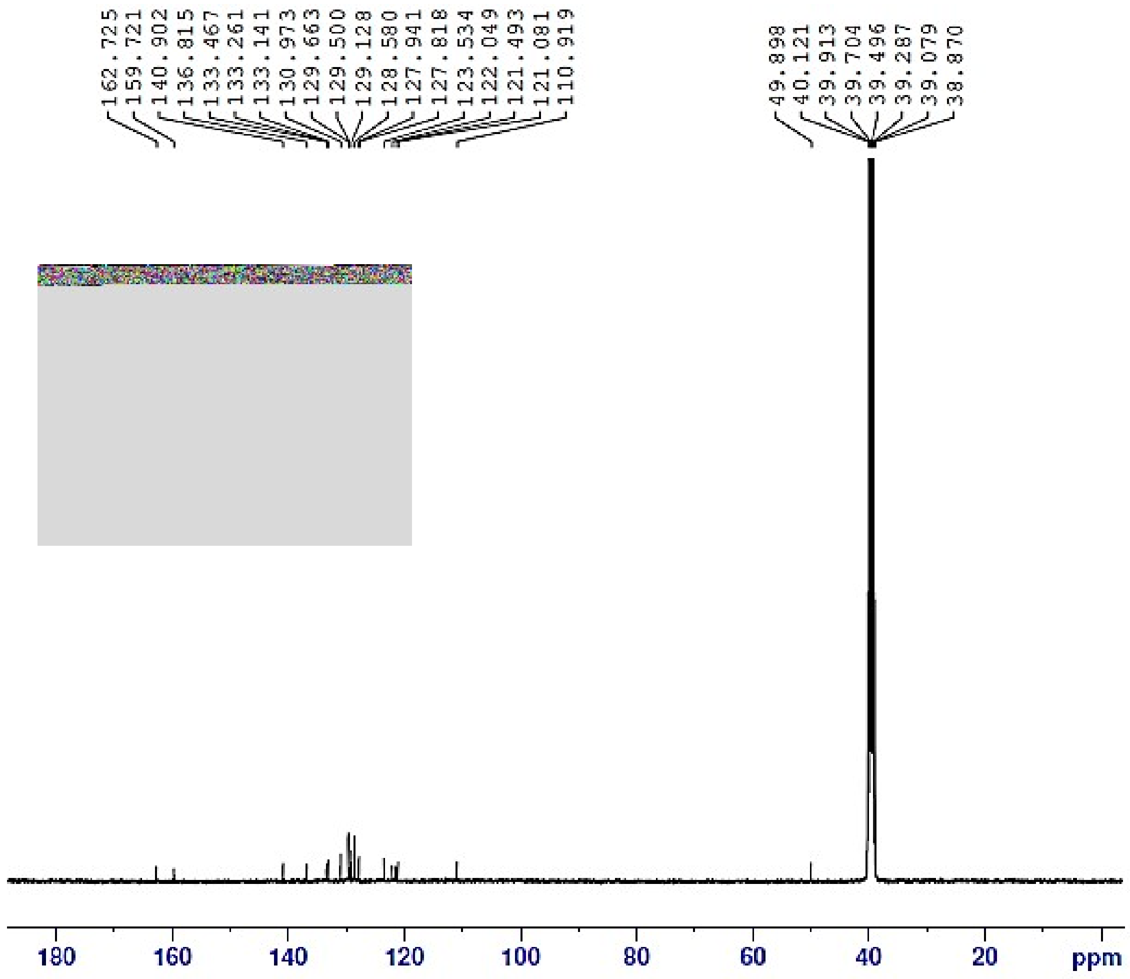

Fig S13: ^{13}CNMR spectra of 2-(4-chlorophenyl)-5-(1-(2,4-dichlorobenzyl)-1*H*-indazol-3-yl)-1,3,4-oxadiazole **(7d)**

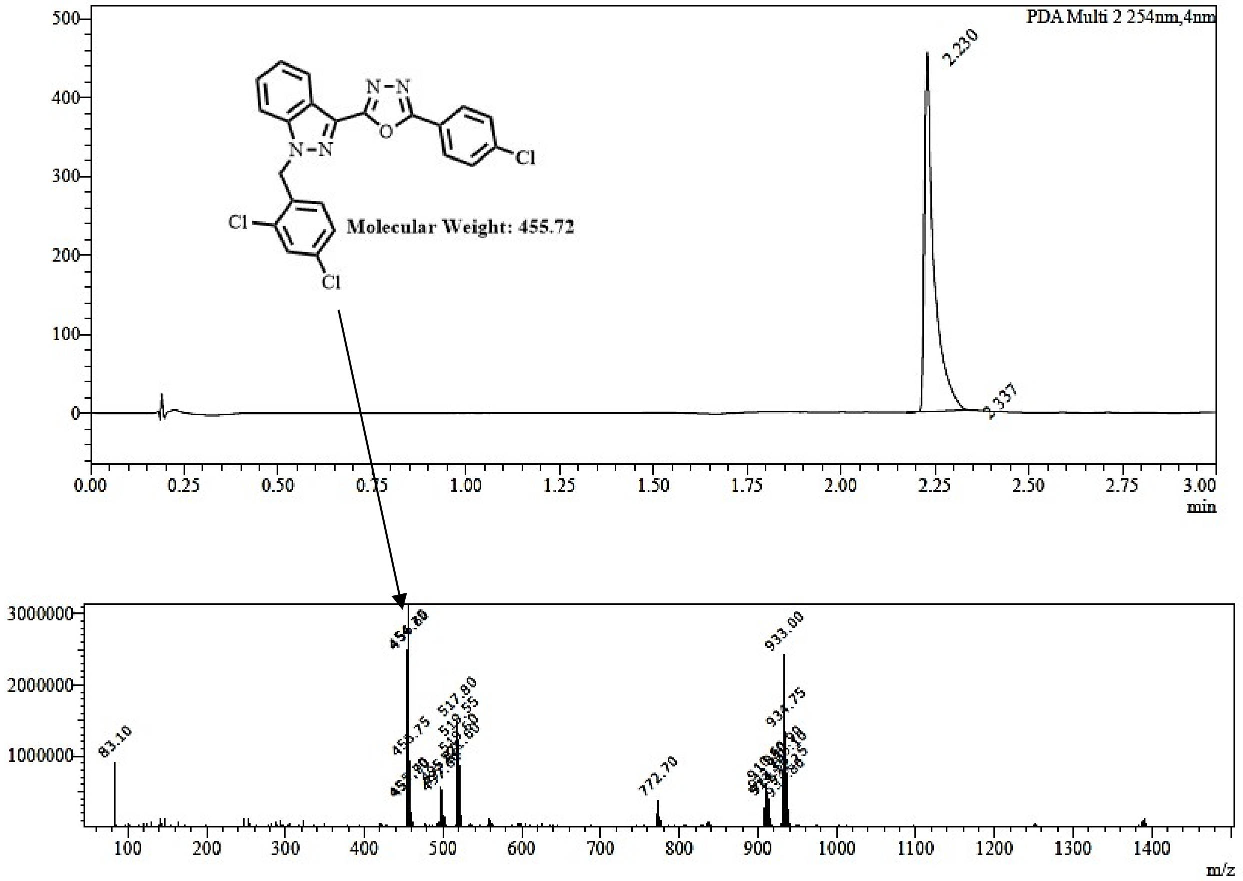

Fig S14: LCMS of 2-(4-chlorophenyl)-5-(1-(2,4-dichlorobenzyl)-1*H*-indazol-3-yl)-1,3,4-oxadiazole **(7d)**

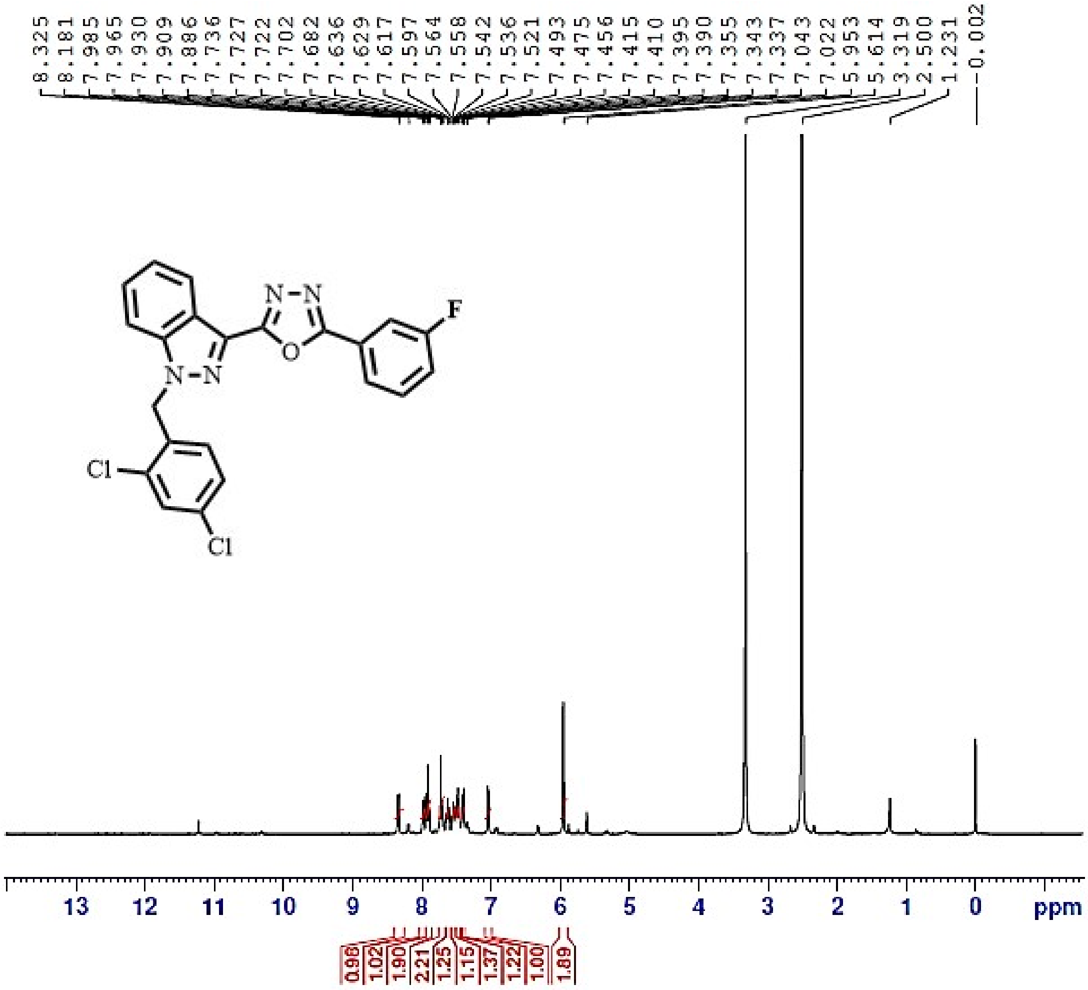

Fig S15: ^{1}HNMR spectra of 2-(1-(2,4-dichlorobenzyl)-1*H*-indazol-3-yl)-5-(3-fluorophenyl)-1,3,4-oxadiazole **(7e)**

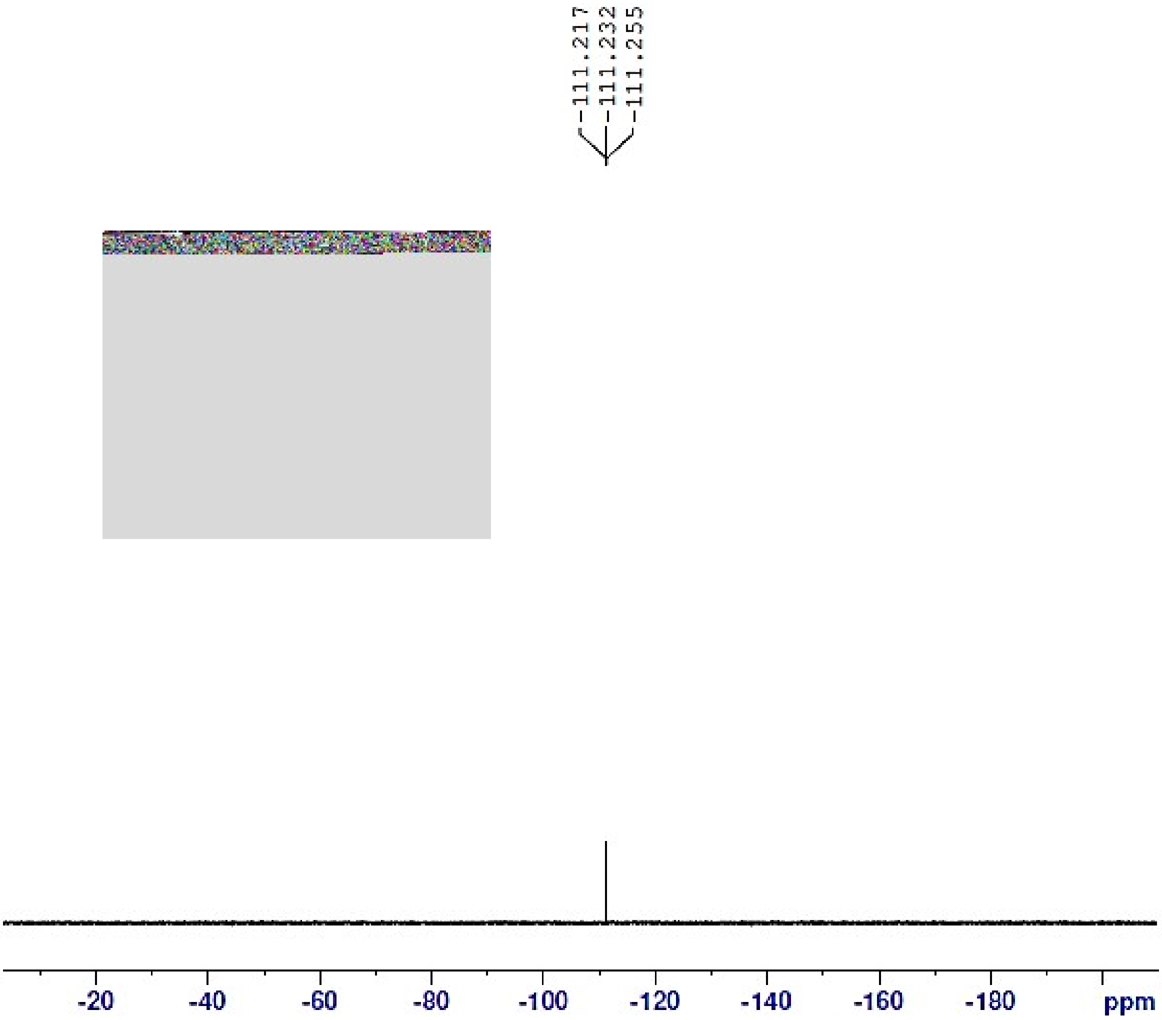

Fig S16: ^{19}FNMR spectra of 2-(1-(2,4-dichlorobenzyl)-1*H*-indazol-3-yl)-5-(3-fluorophenyl)-1,3,4-oxadiazole **(7e)**

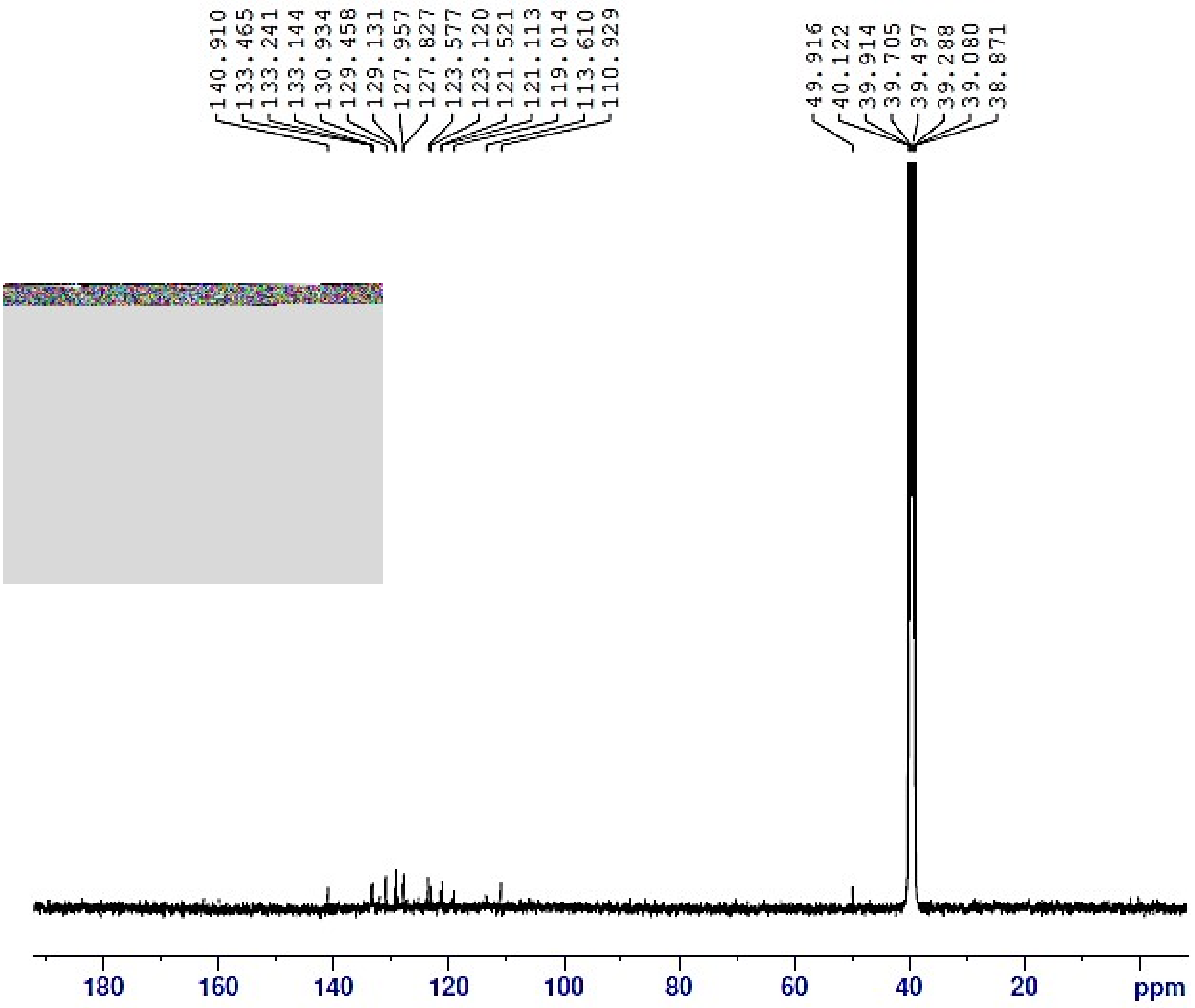

Fig S17: ^{13}CNMR spectra of 2-(1-(2,4-dichlorobenzyl)-1*H*-indazol-3-yl)-5-(3-fluorophenyl)-1,3,4-oxadiazole **(7e)**

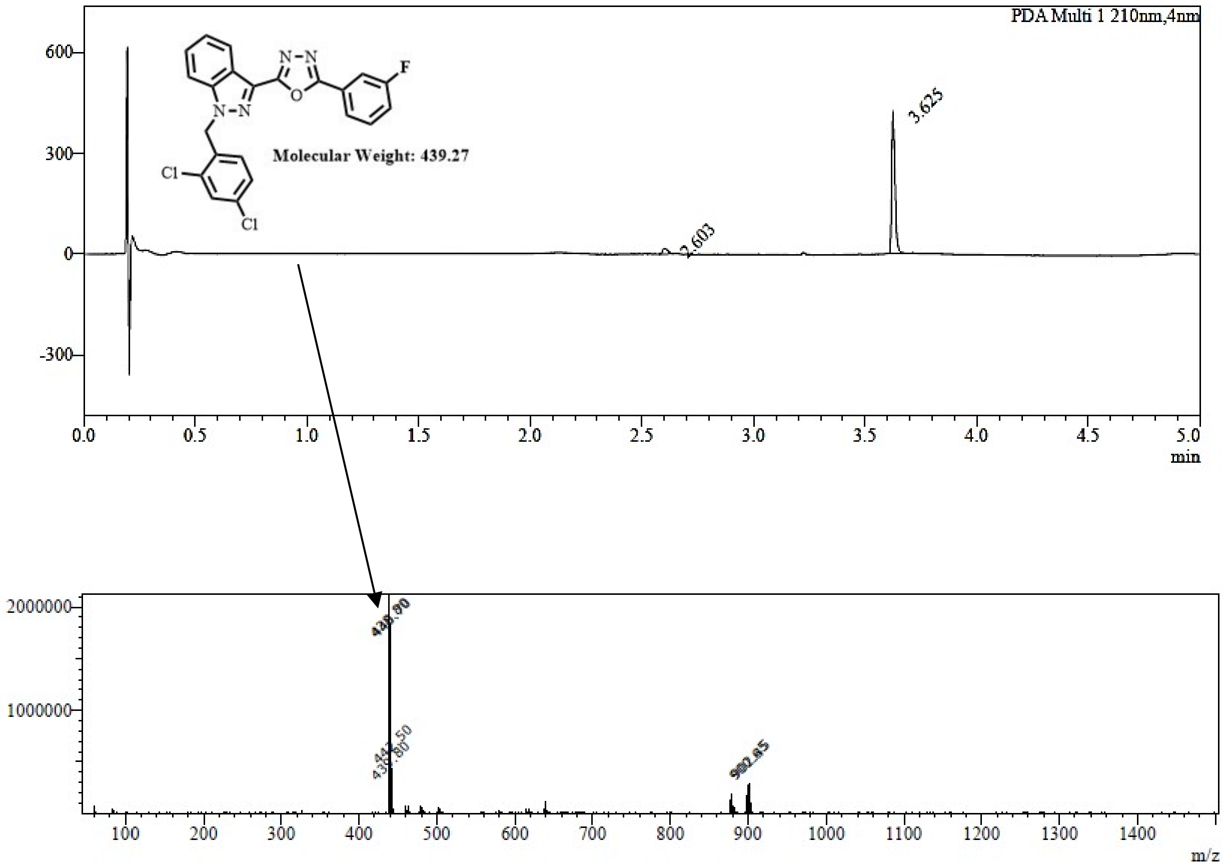

Fig S18: LCMS of 2-(1-(2,4-dichlorobenzyl)-1*H*-indazol-3-yl)-5-(3-fluorophenyl)-1,3,4-oxadiazole **(7e)**

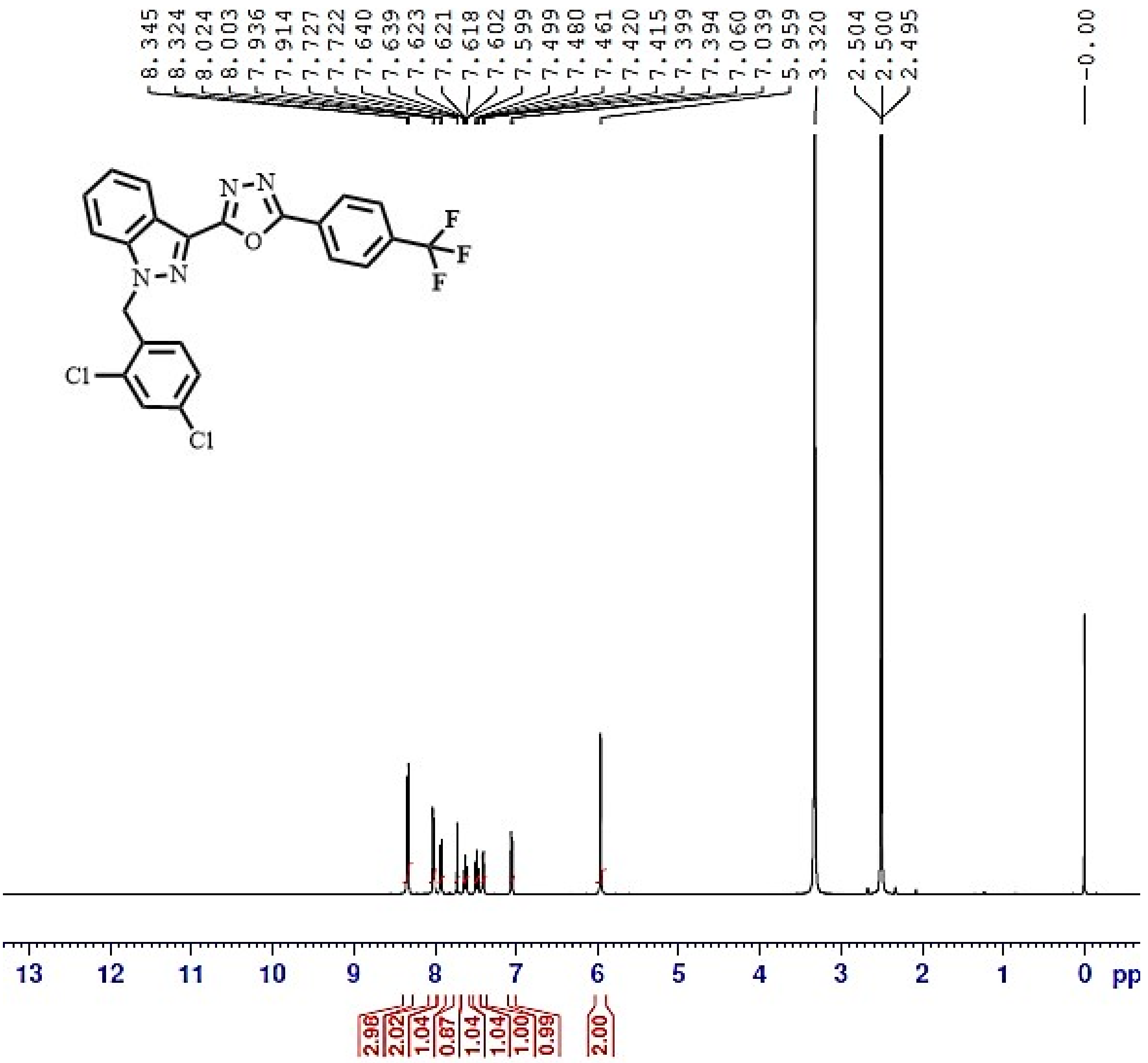

Fig S19: ^{1}HNMR spectra of 2-(1-(2,4-dichlorobenzyl)-1*H*-indazol-3-yl)-5-(4-(trifluoromethyl) phenyl)-1,3,4-oxadiazole **(7f)**

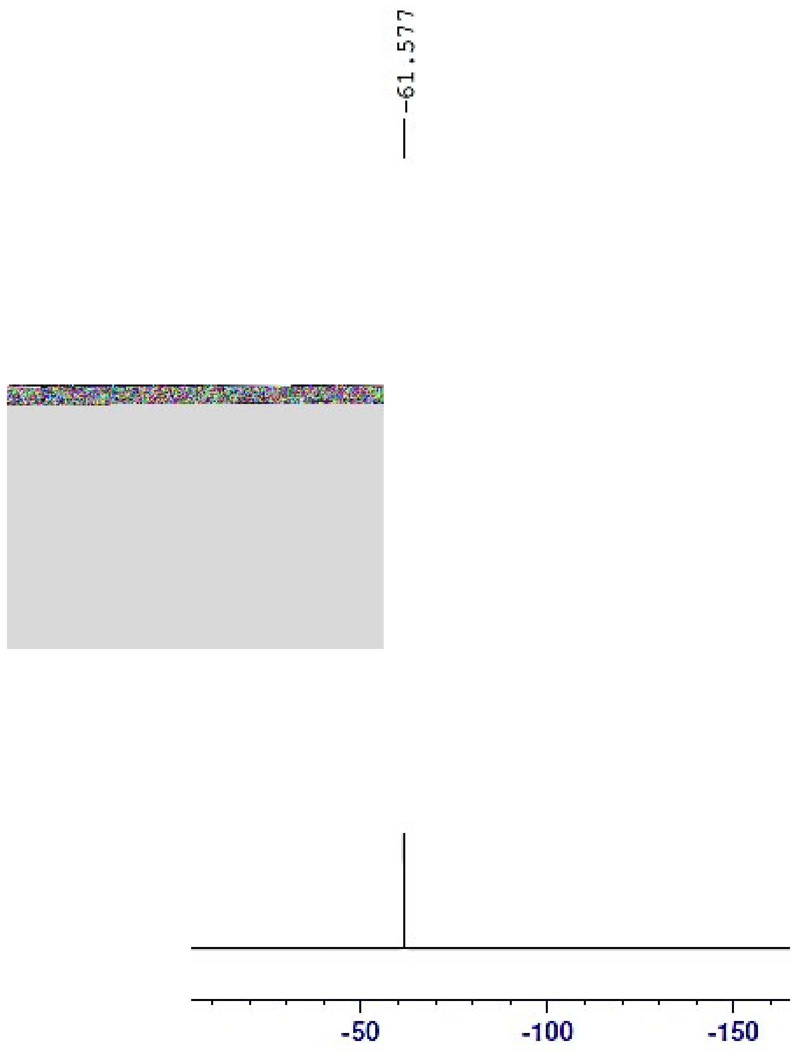

Fig S20: ¹⁹FNMR spectra of 2-(1-(2,4-dichlorobenzyl)-1*H*-indazol-3-yl)-5-(4-(trifluoromethyl) phenyl)-1,3,4-oxadiazole **(7f)**

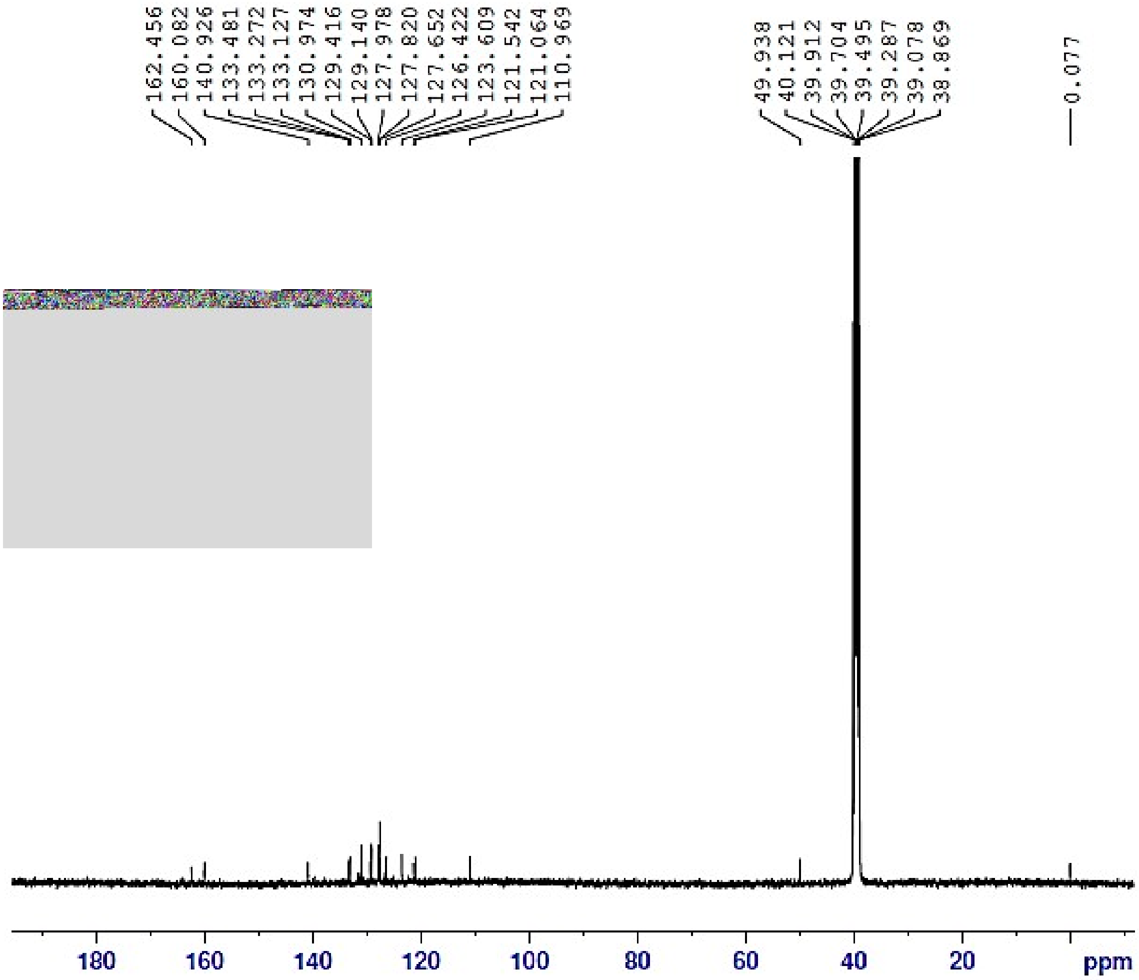

Fig S21: ^{13}CNMR spectra of 2-(1-(2,4-dichlorobenzyl)-1*H*-indazol-3-yl)-5-(4-(trifluoromethyl) phenyl)-1,3,4-oxadiazole **(7f)**

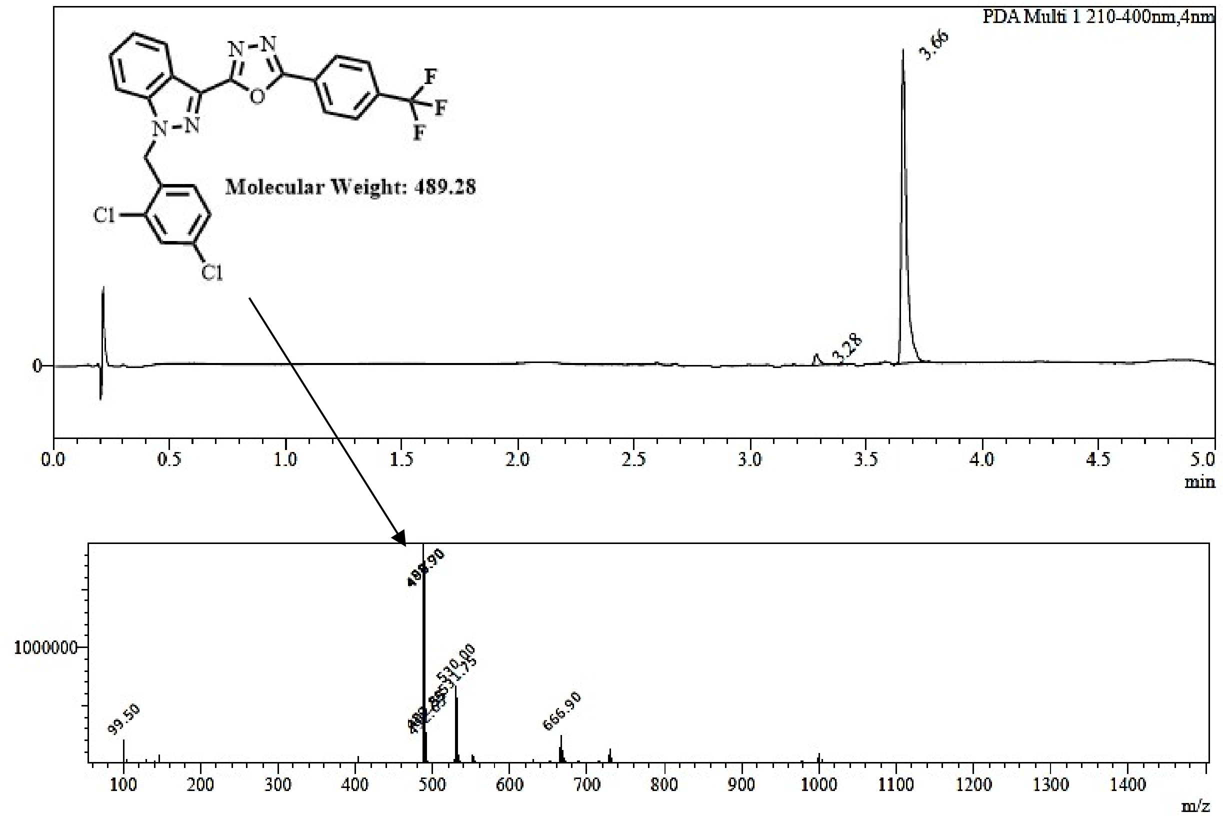

Fig S22: LCMS of 2-(1-(2,4-dichlorobenzyl)-1*H*-indazol-3-yl)-5-(4-(trifluoromethyl) phenyl)-1,3,4-oxadiazole **(7f)**

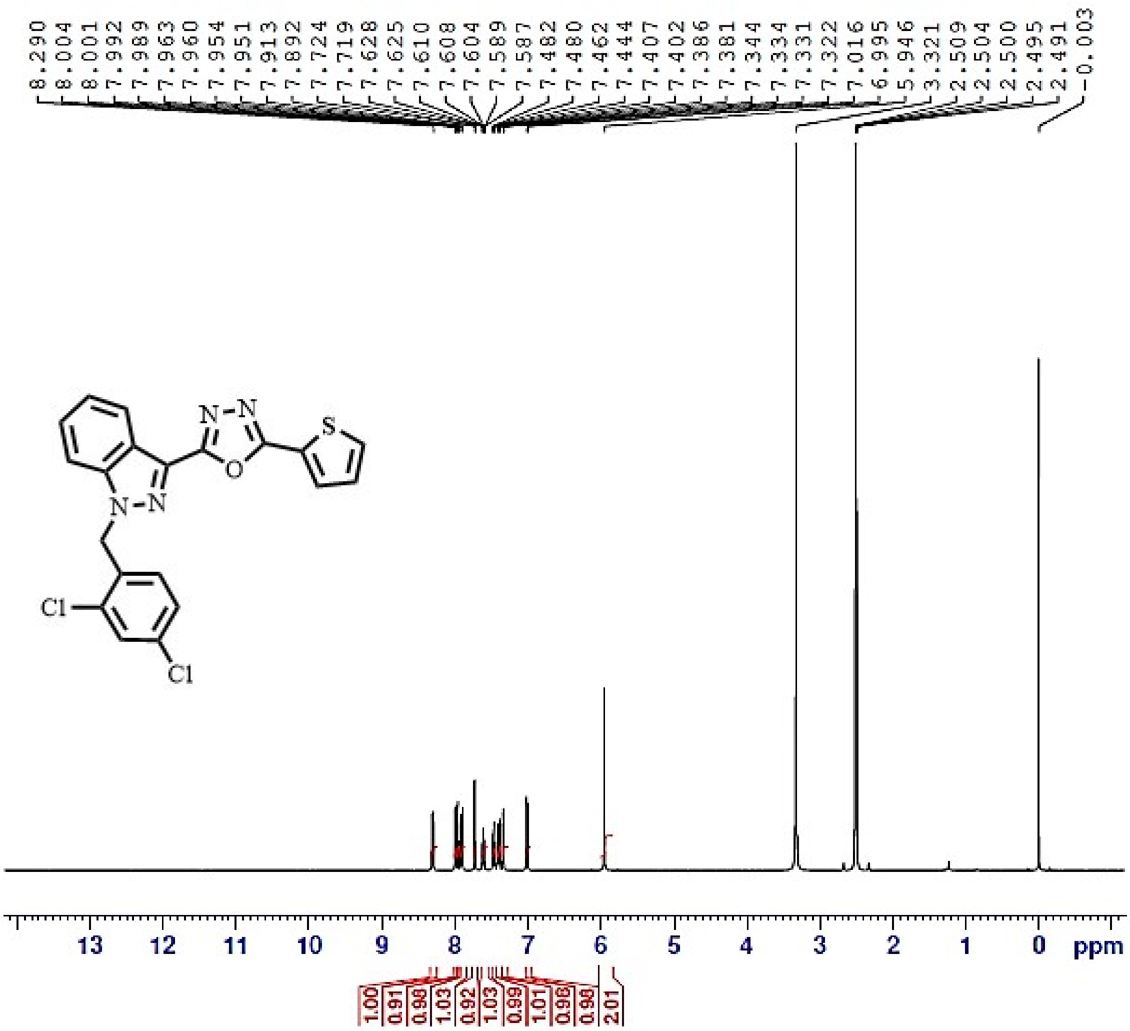

Fig S23: ^{1}HNMR spectra of 2-(1-(2,4-dichlorobenzyl)-1*H*-indazol-3-yl)-5-(thiophen-2-yl)-1,3,4-oxadiazole **(7g)**

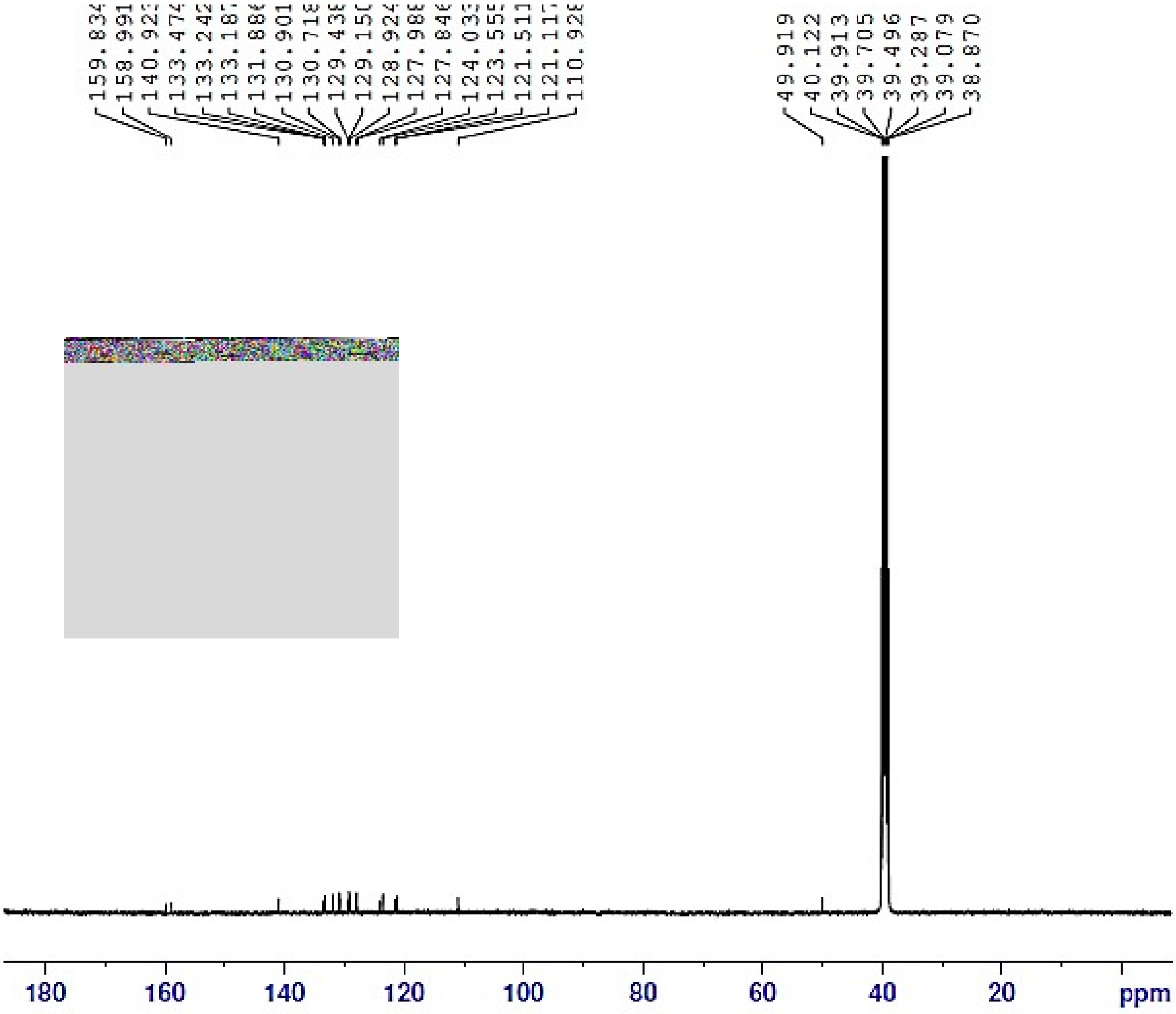

Fig S24: ^{13}CNMR spectra of 2-(1-(2,4-dichlorobenzyl)-1*H*-indazol-3-yl)-5-(thiophen-2-yl)-1,3,4-oxadiazole **(7g)**

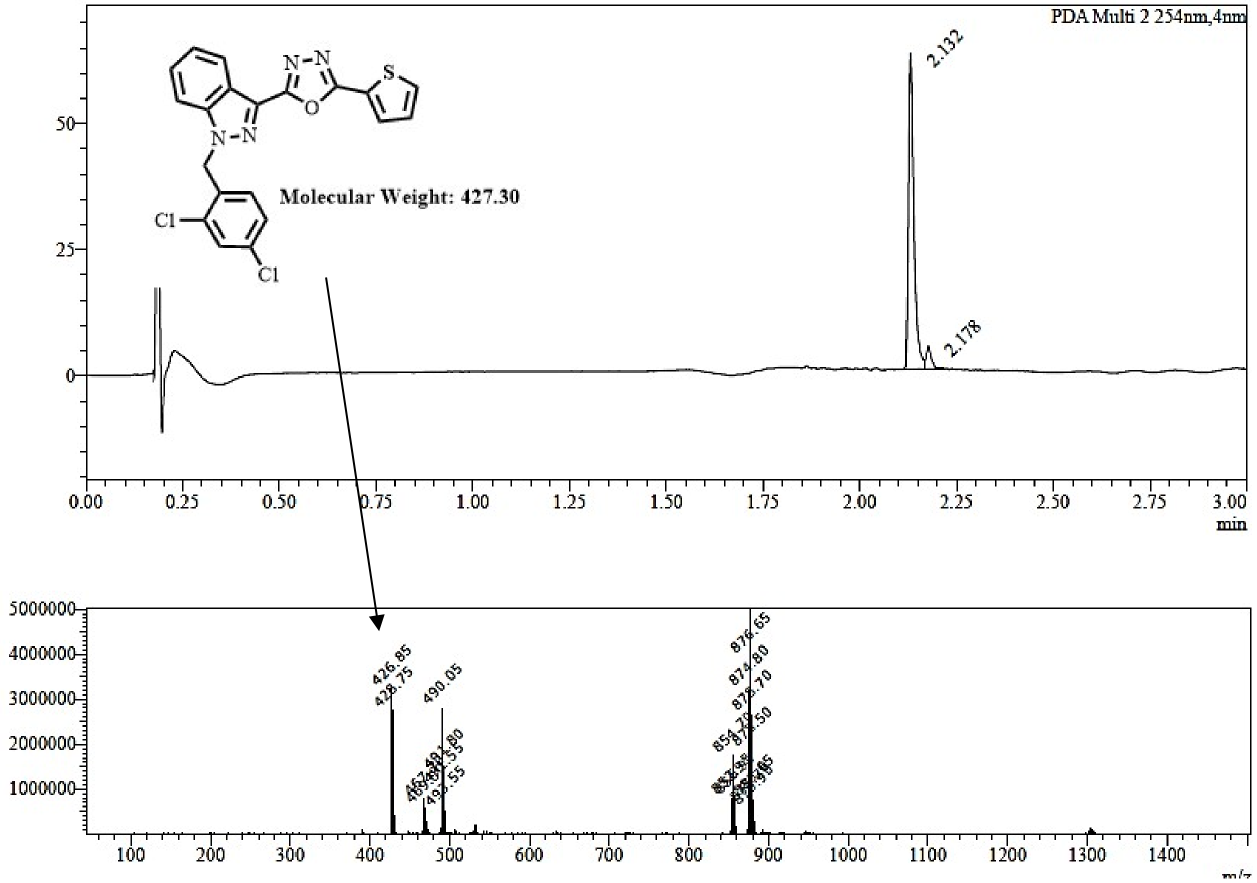

Fig S25: LCMS of 2-(1-(2,4-dichlorobenzyl)-1*H*-indazol-3-yl)-5-(thiophen-2-yl)-1,3,4-oxadiazole **(7g)**

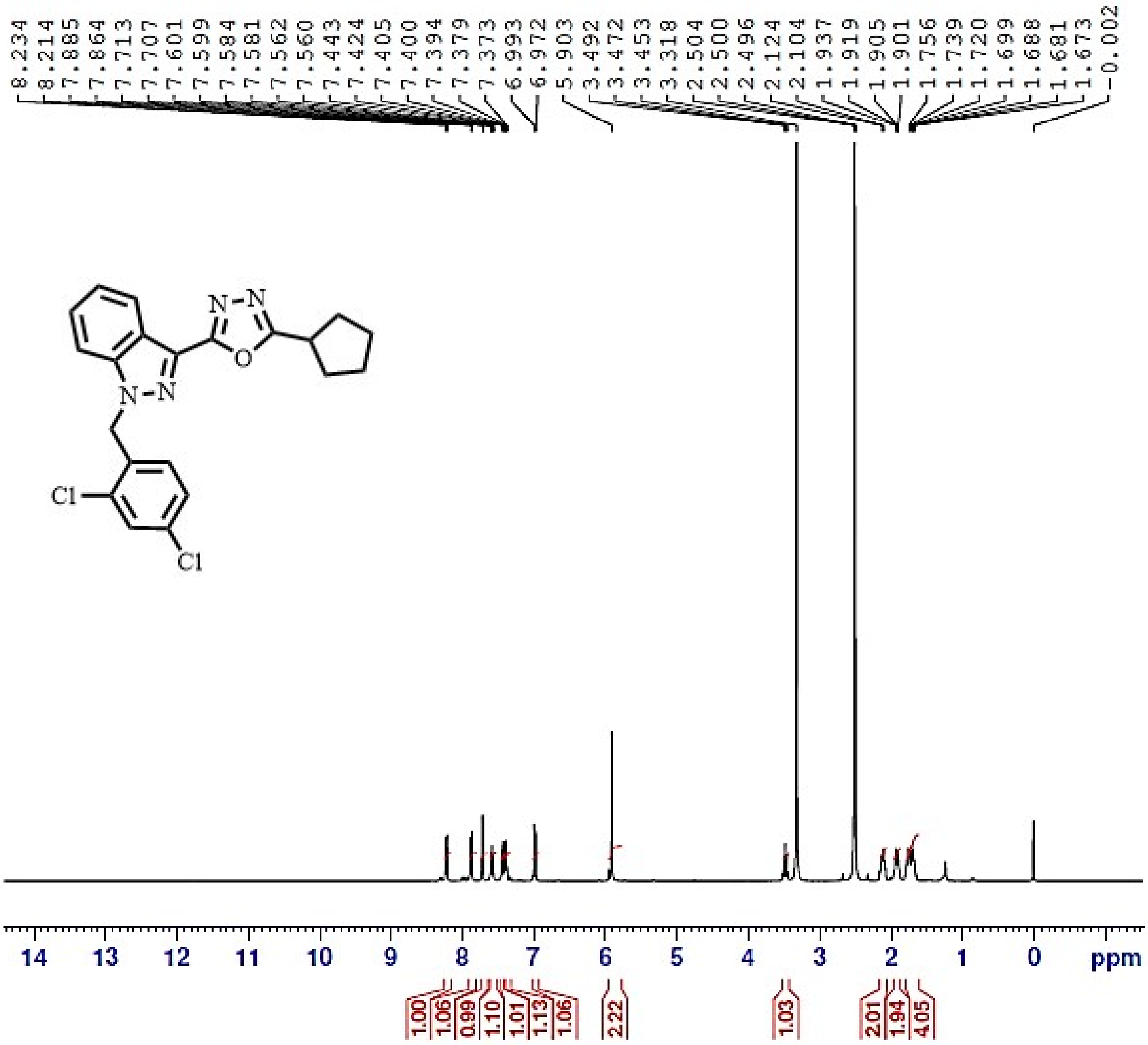

Fig S26: ^{1}HNMR spectra of 2-cyclopentyl-5-(1-(2,4-dichlorobenzyl)-1*H*-indazol-3-yl)-1,3,4-oxadiazole **(7h)**

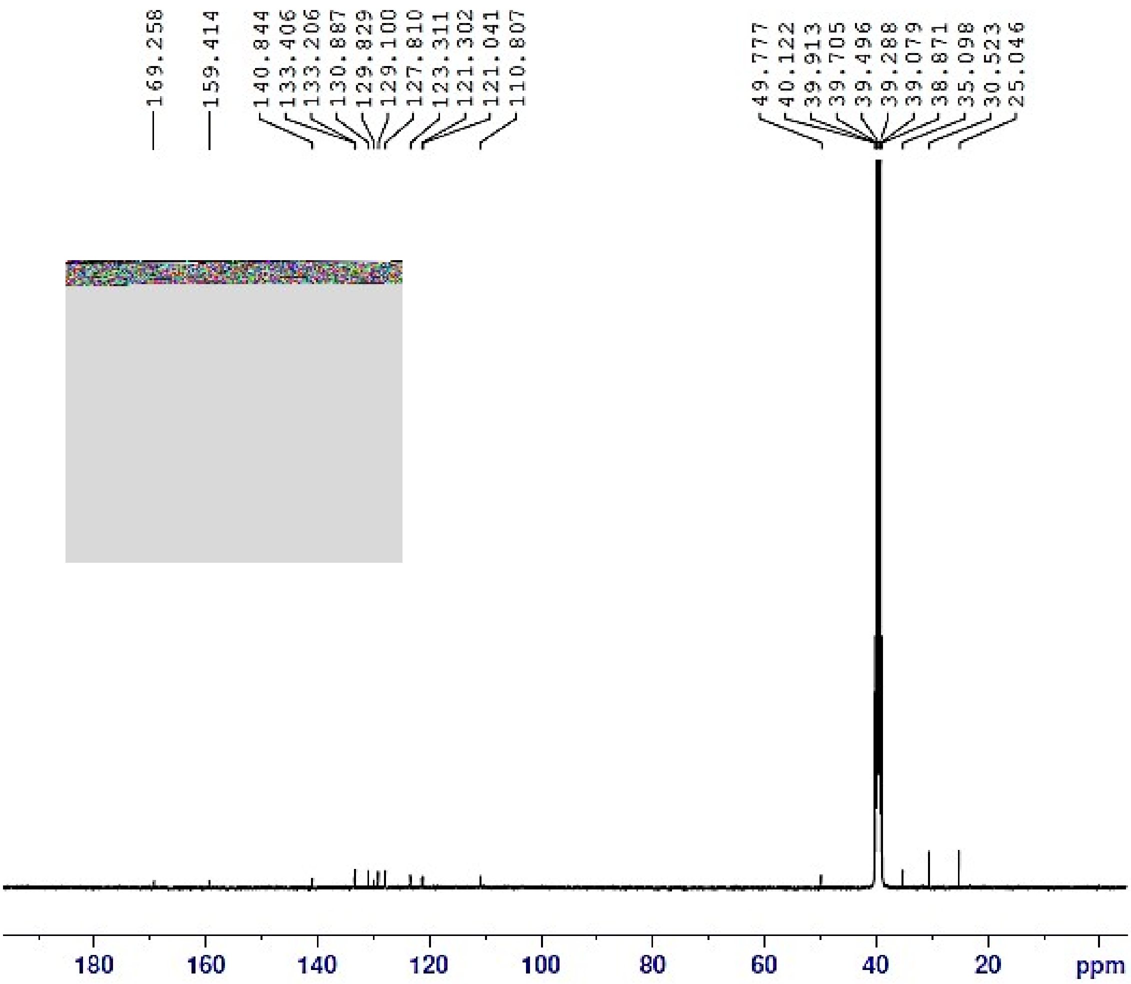

Fig S27: ^{13}CNMR spectra of 2-cyclopentyl-5-(1-(2,4-dichlorobenzyl)-1*H*-indazol-3-yl)-1,3,4-oxadiazole **(7h)**

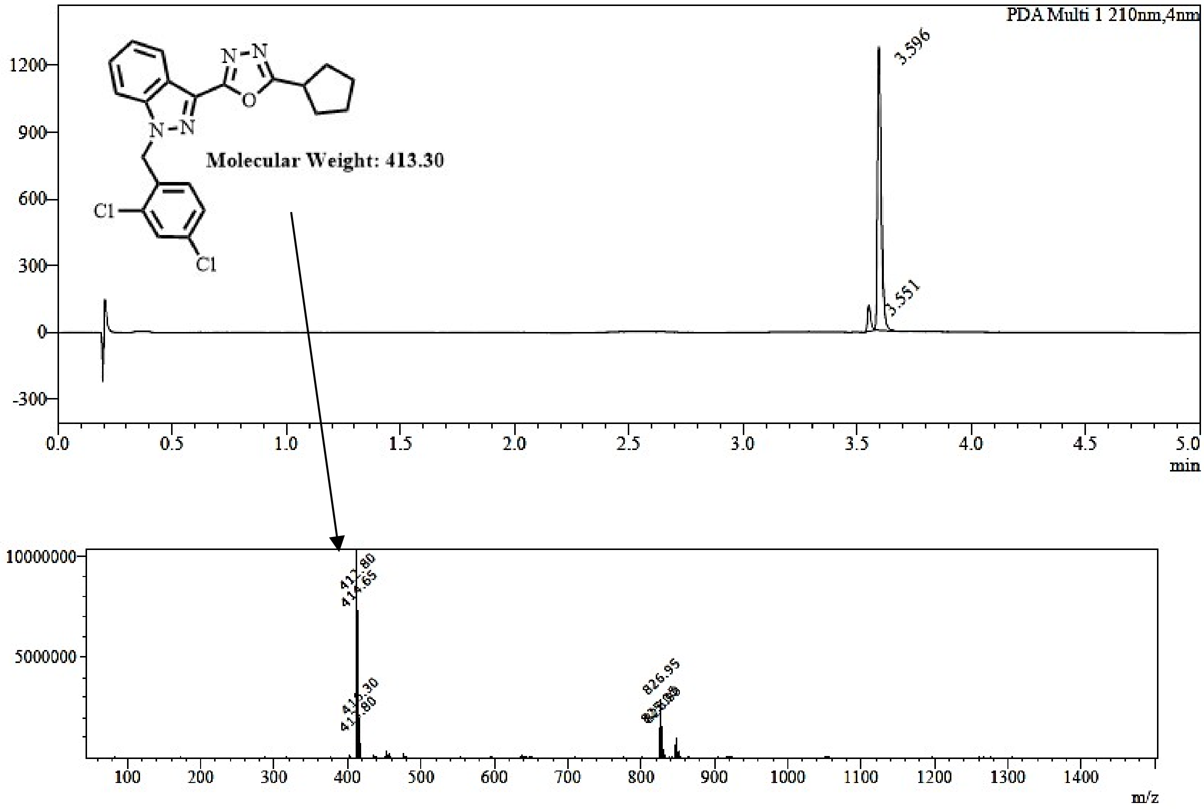

Fig S28: LCMS of 2-cyclopentyl-5-(1-(2,4-dichlorobenzyl)-1*H*-indazol-3-yl)-1,3,4-oxadiazole **(7h)**

2: Molecular Docking results of compounds 7(a-h)

Positive Control (LND)

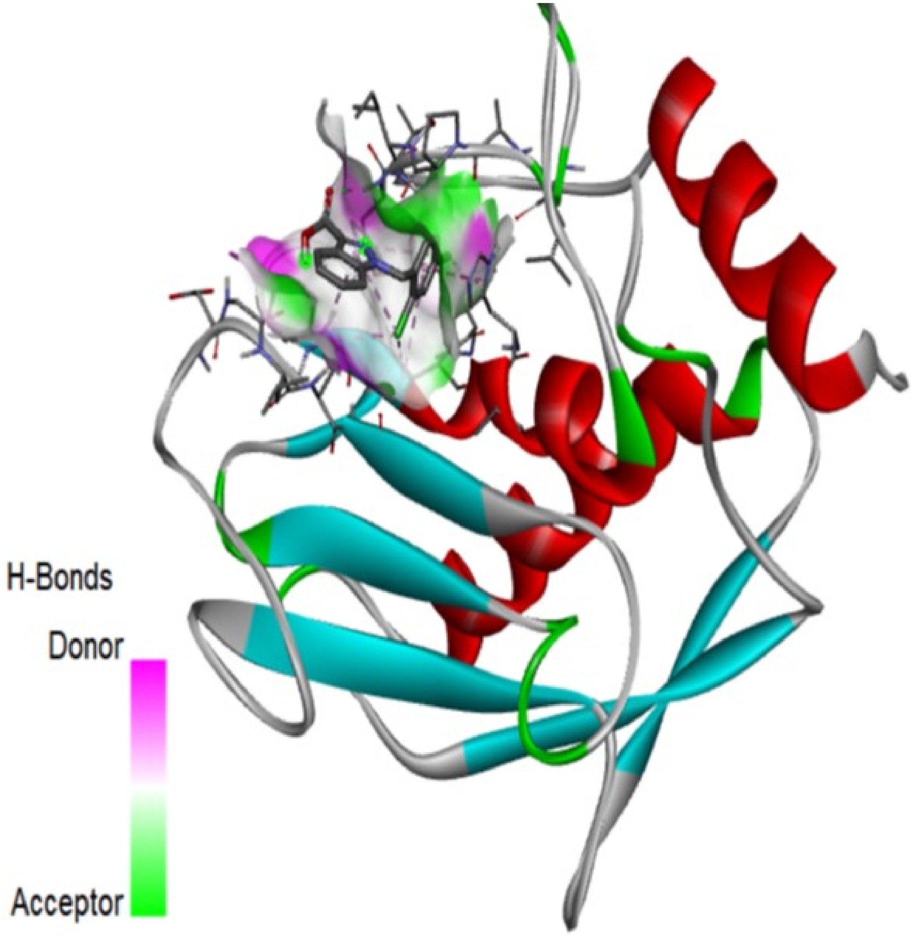

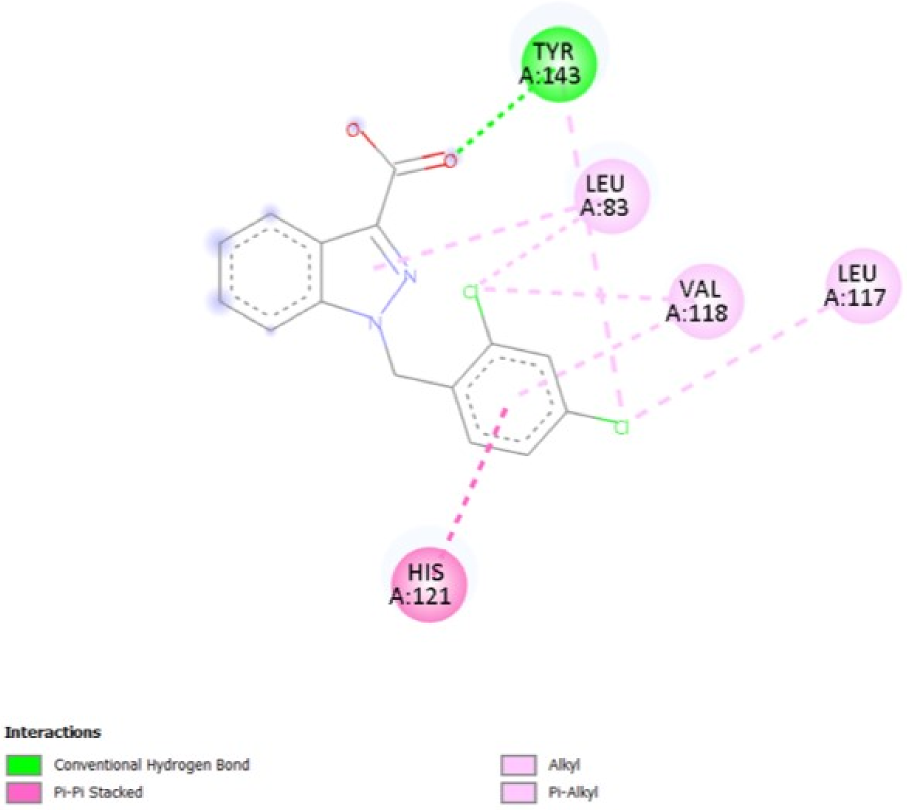

7a

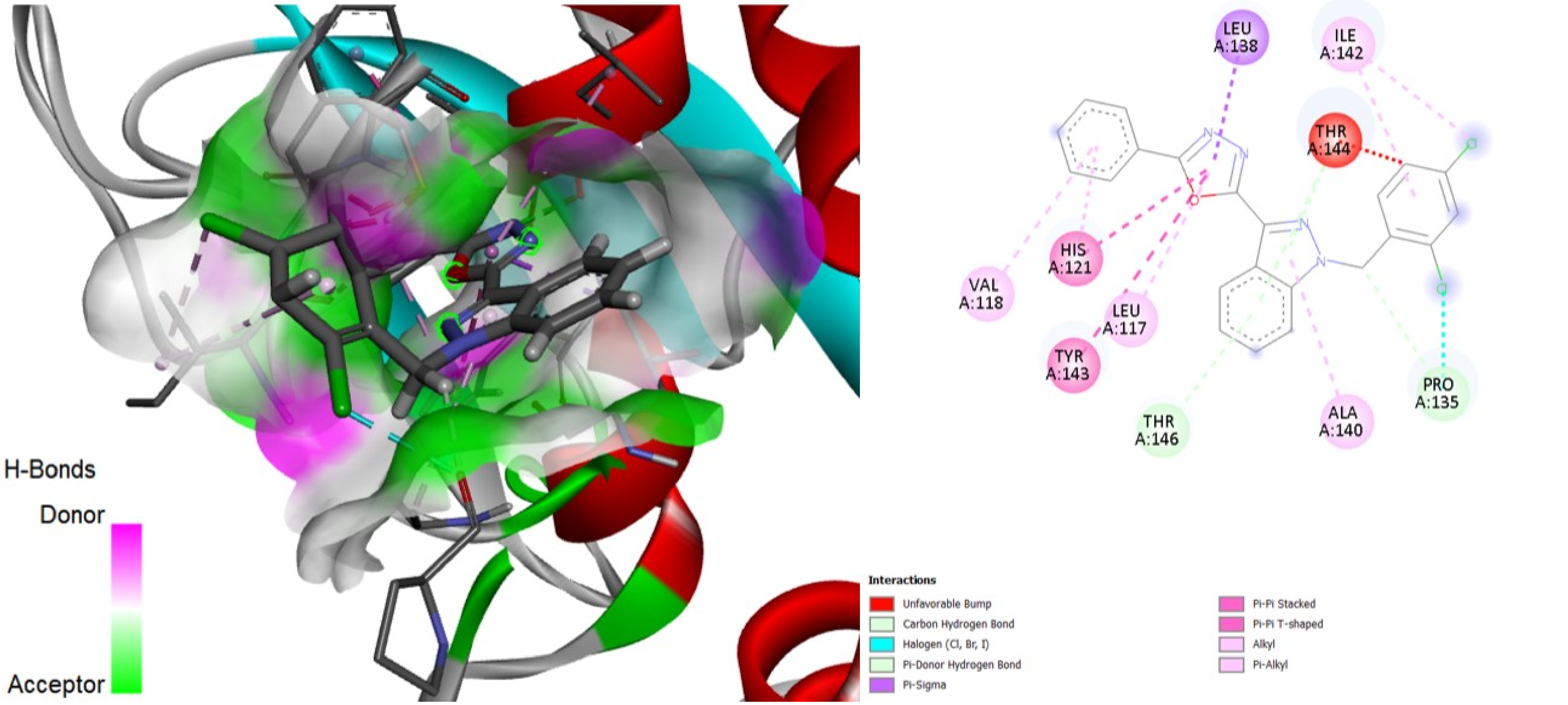
H-Bonds
Donor
Acceptor
LEU A:138
ILE A:142
THR A:144
HIS A:121
VAL A:118
LEU A:117
TYR A:143
THR A:146
ALA A:140
PRO A:135
Interactions
Unfavorable Bump
Carbon Hydrogen Bond
Halogen (Cl, Br, I)
Pi-Donor Hydrogen Bond
Pi-Sigma
Pi-Pi Stacked
Pi-Pi T-shaped
Alkyl
Pi-Alkyl

7b

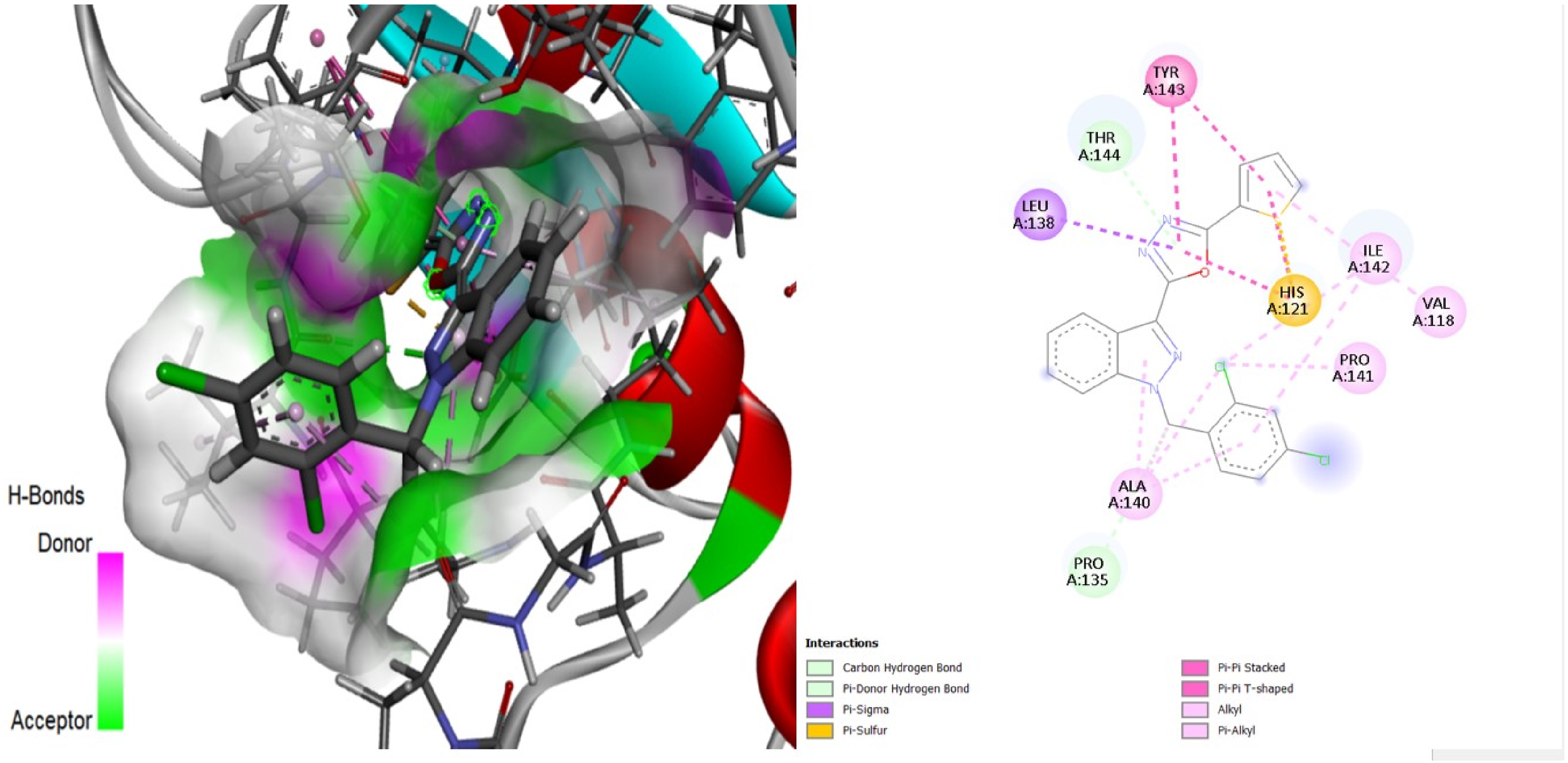
H-Bonds
Donor
Acceptor
TYR A:143
THR A:144
LEU A:138
ILE A:142
HIS A:121
VAL A:118
PRO A:141
ALA A:140
PRO A:135
Interactions
Carbon Hydrogen Bond
Pi-Donor Hydrogen Bond
Pi-Sigma
Pi-Sulfur
Pi-Pi Stacked
Pi-Pi T-shaped
Alkyl
Pi-Alkyl

7c

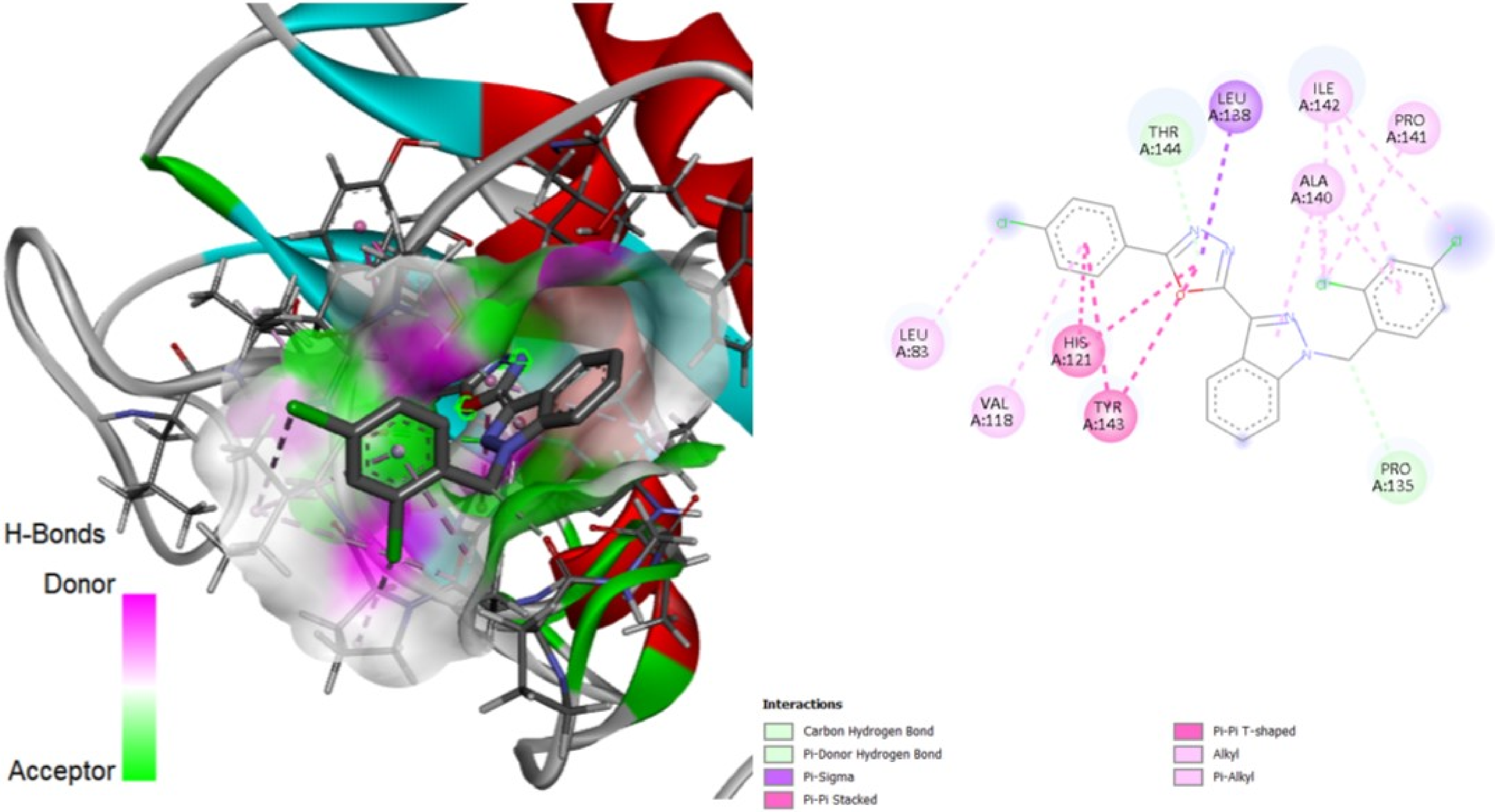
H-Bonds
Donor
Acceptor
THR A:144
LEU A:138
ILE A:142
PRO A:141
ALA A:140
LEU A:83
HIS A:121
VAL A:118
TYR A:143
PRO A:135
Interactions
Carbon Hydrogen Bond
Pi-Donor Hydrogen Bond
Pi-Sigma
Pi-Pi Stacked
Pi-Pi T-shaped
Alkyl
Pi-Alkyl

7d

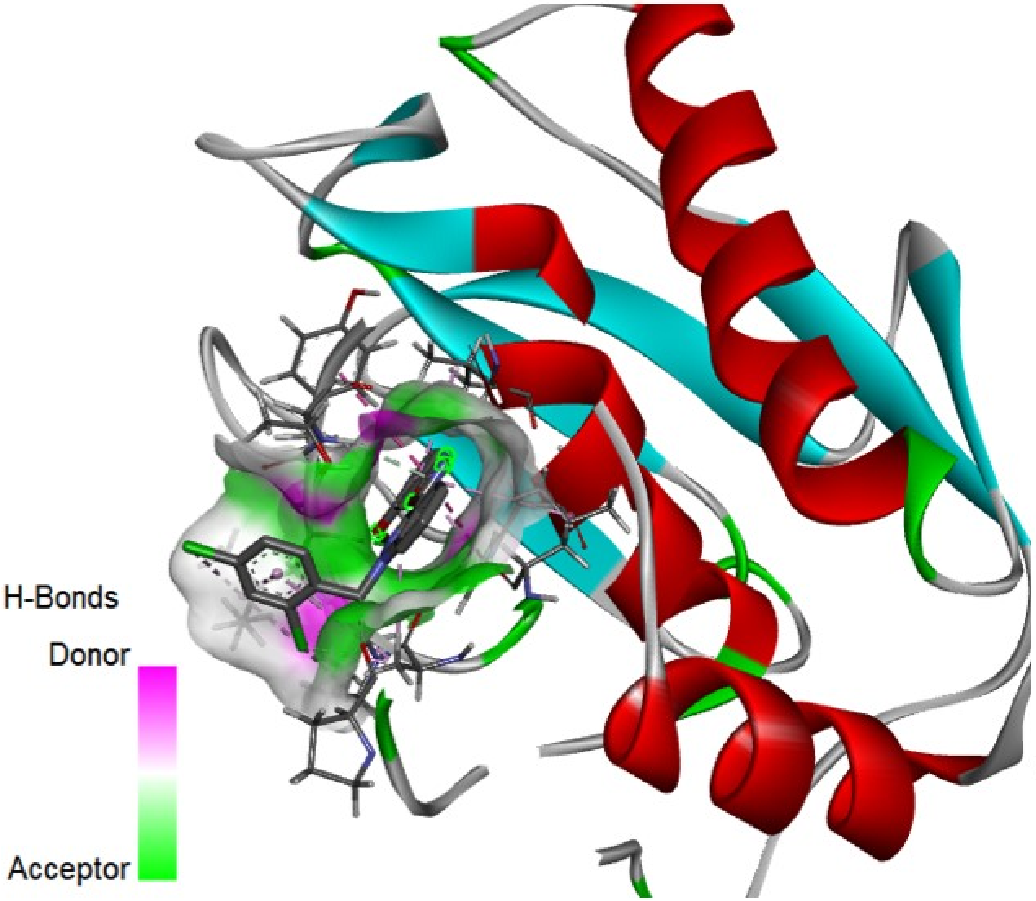
H-Bonds
Donor
Acceptor

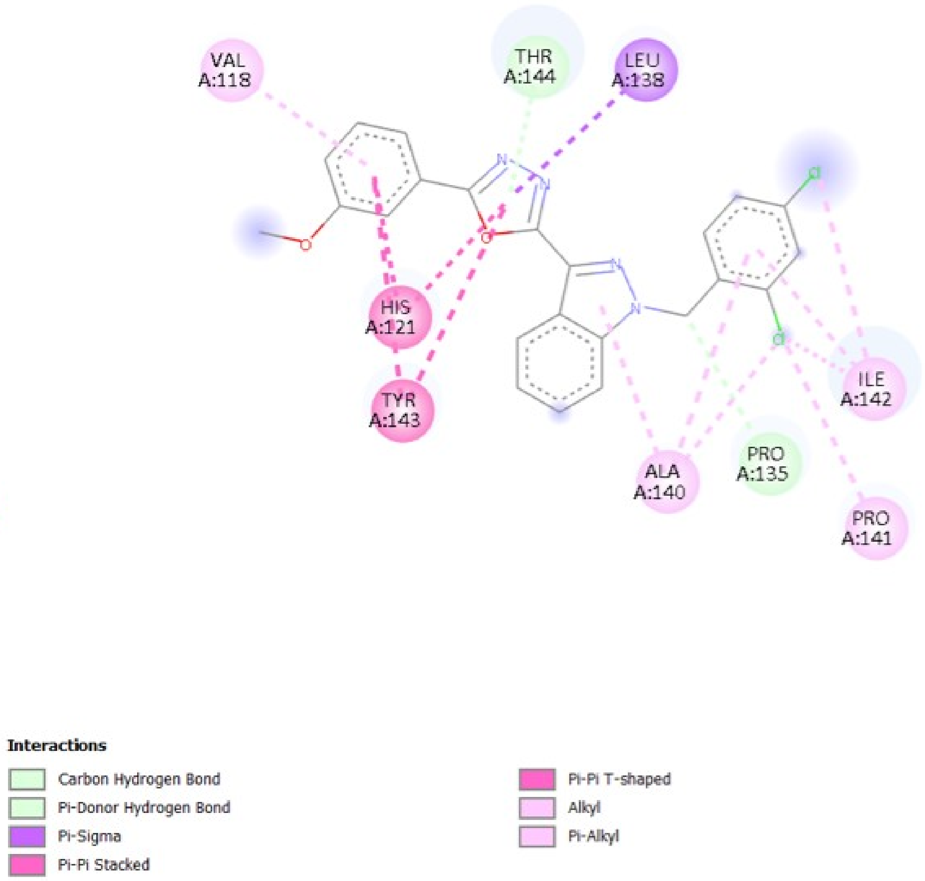
VAL A:118
THR A:144
LEU A:138
HIS A:121
TYR A:143
ILE A:142
ALA A:140
PRO A:135
PRO A:141
Interactions
Carbon Hydrogen Bond
Pi-Donor Hydrogen Bond
Pi-Sigma
Pi-Pi Stacked
Pi-Pi T-shaped
Alkyl
Pi-Alkyl

7e

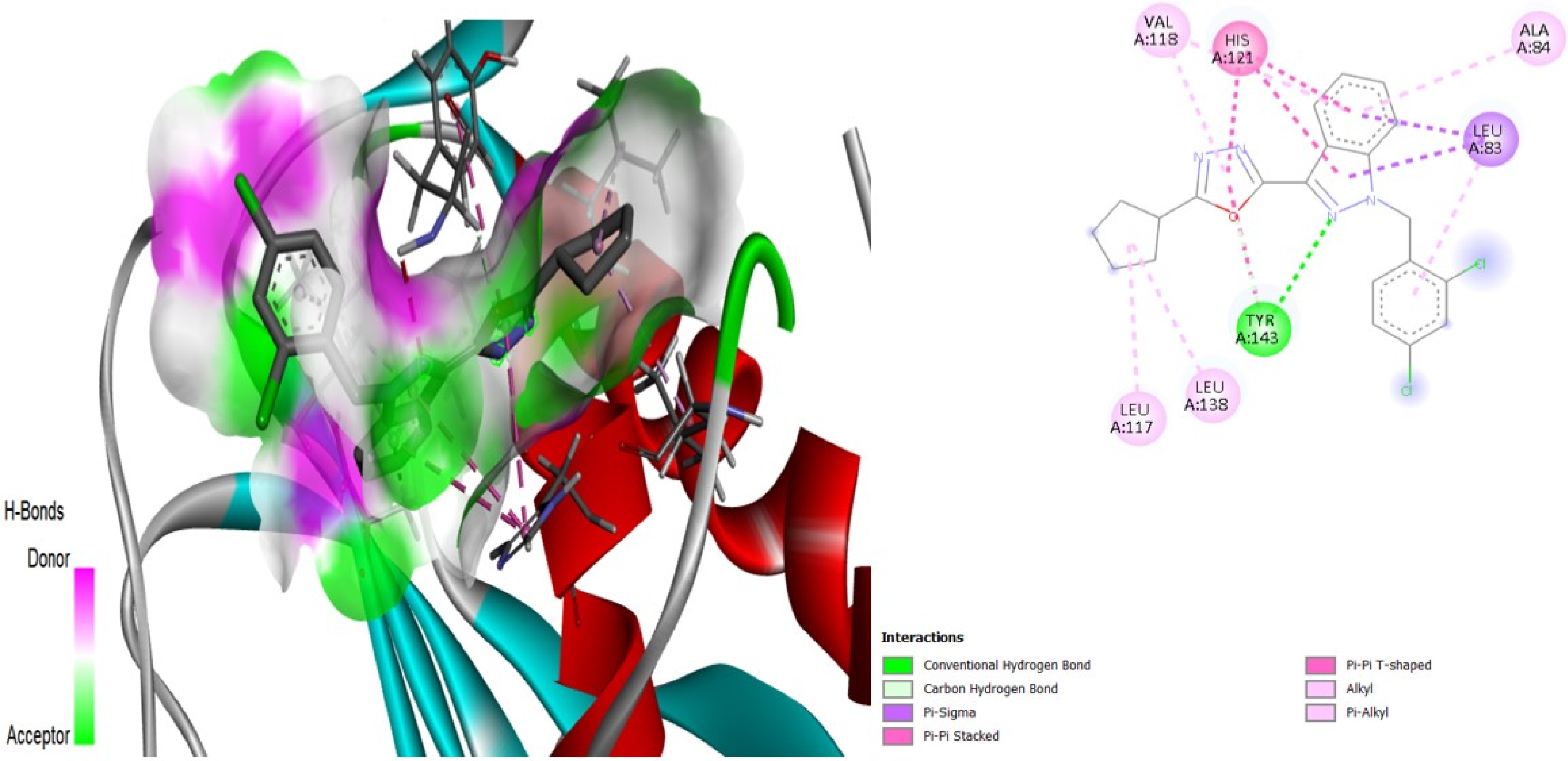
H-Bonds
Donor
Acceptor
VAL A:118
HIS A:121
ALA A:84
LEU A:83
TYR A:143
LEU A:138
LEU A:117
Interactions
Conventional Hydrogen Bond
Carbon Hydrogen Bond
Pi-Sigma
Pi-Pi Stacked
Pi-Pi T-shaped
Alkyl
Pi-Alkyl

7f

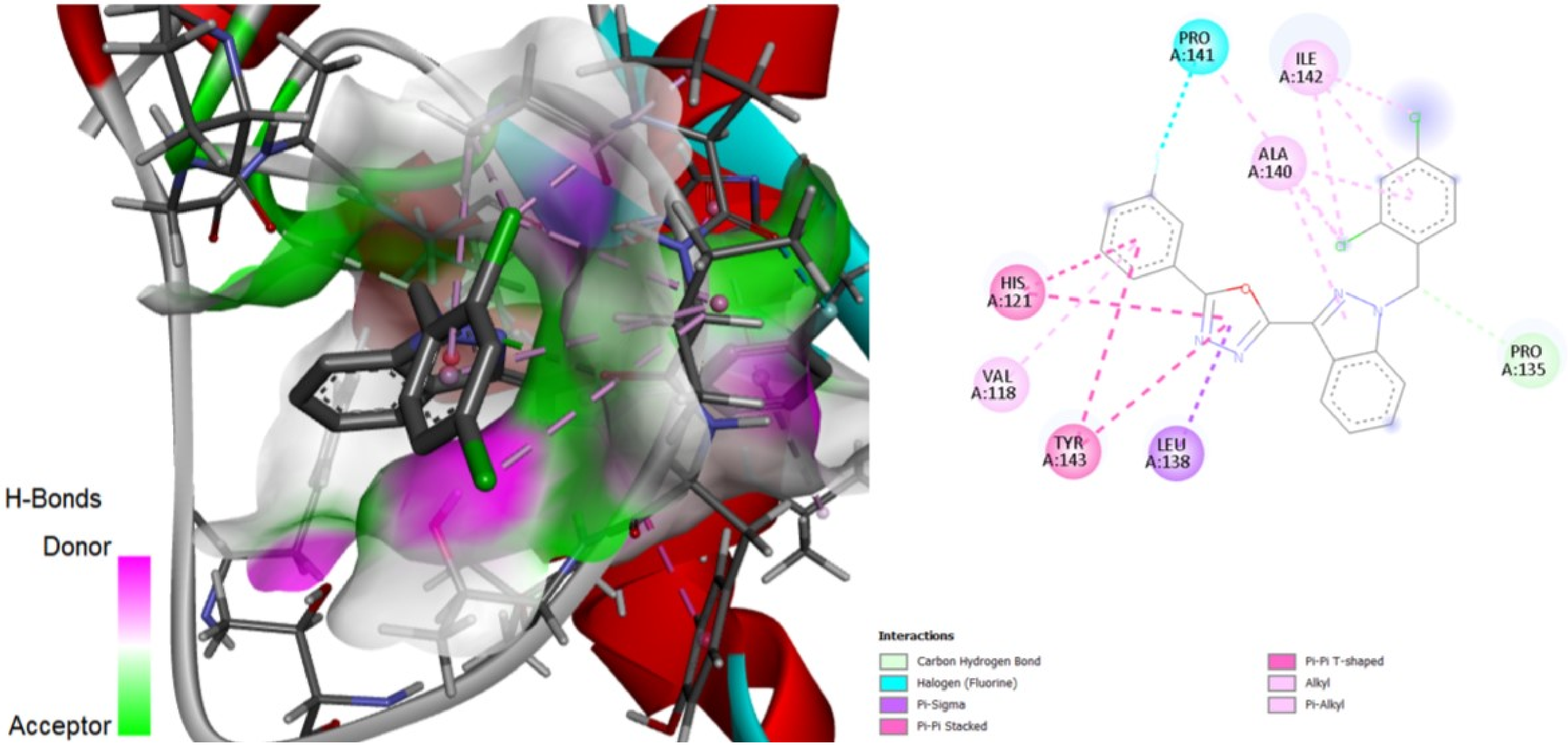
H-Bonds
Donor
Acceptor
PRO A:141
ILE A:142
ALA A:140
HIS A:121
VAL A:118
TYR A:143
LEU A:138
PRO A:135
Interactions
Carbon Hydrogen Bond
Halogen (Fluorine)
Pi-Sigma
Pi-Pi Stacked
Pi-Pi T-shaped
Alkyl
Pi-Alkyl

7g

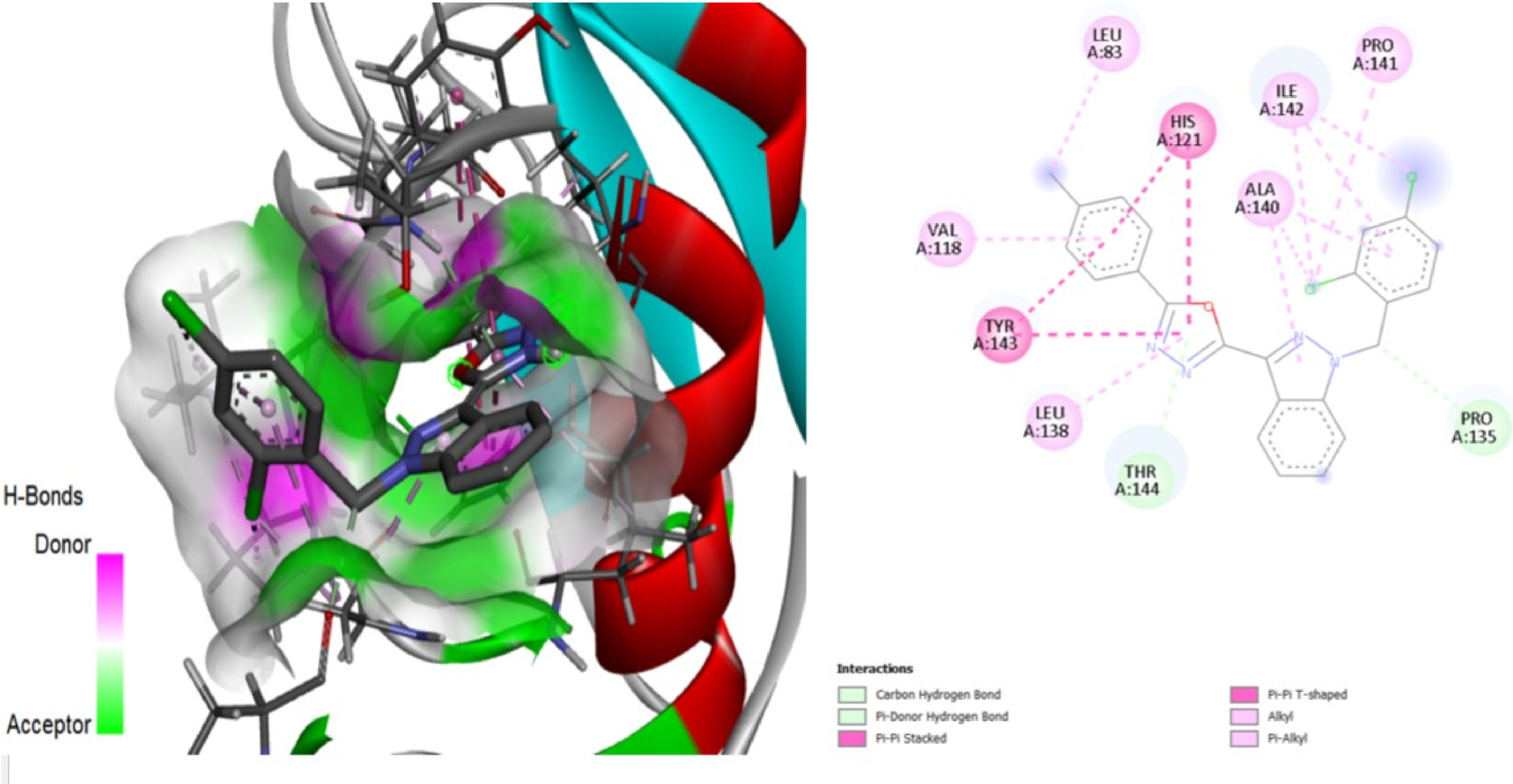
H-Bonds
Donor
Acceptor
LEU A:83
PRO A:141
ILE A:142
HIS A:121
ALA A:140
VAL A:118
TYR A:143
LEU A:138
THR A:144
PRO A:135
Interactions
Carbon Hydrogen Bond
Pi-Donor Hydrogen Bond
Pi-Pi Stacked
Pi-Pi T-shaped
Alkyl
Pi-Alkyl

7h

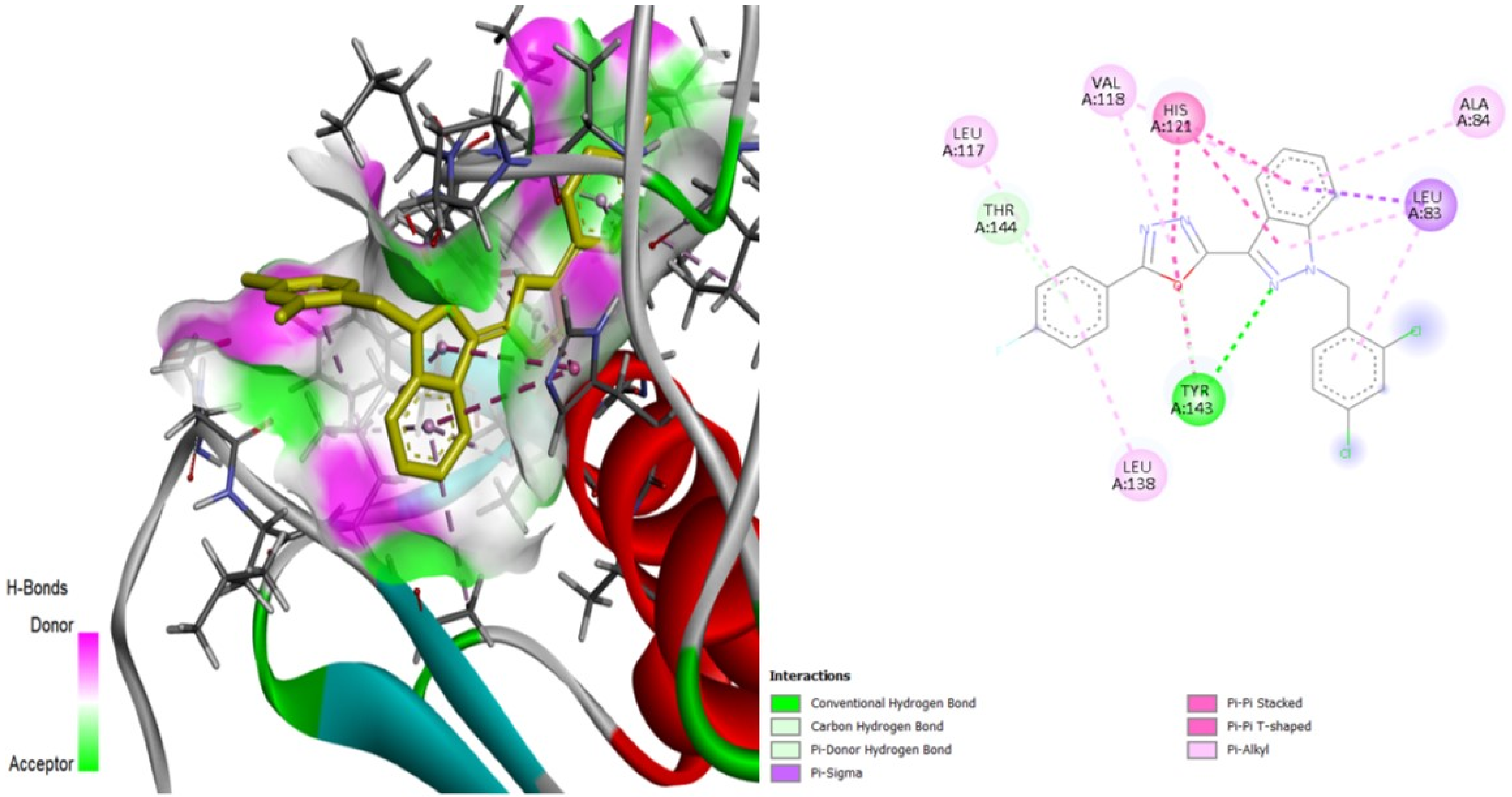
H-Bonds
Donor
Acceptor
VAL A:118
HIS A:121
ALA A:84
LEU A:117
THR A:144
LEU A:83
TYR A:143
LEU A:138
Interactions
Conventional Hydrogen Bond
Carbon Hydrogen Bond
Pi-Donor Hydrogen Bond
Pi-Sigma
Pi-Pi Stacked
Pi-Pi T-shaped
Pi-Alkyl

3. Bioavailability radar chart of all derivatives from the Swiss ADME web server

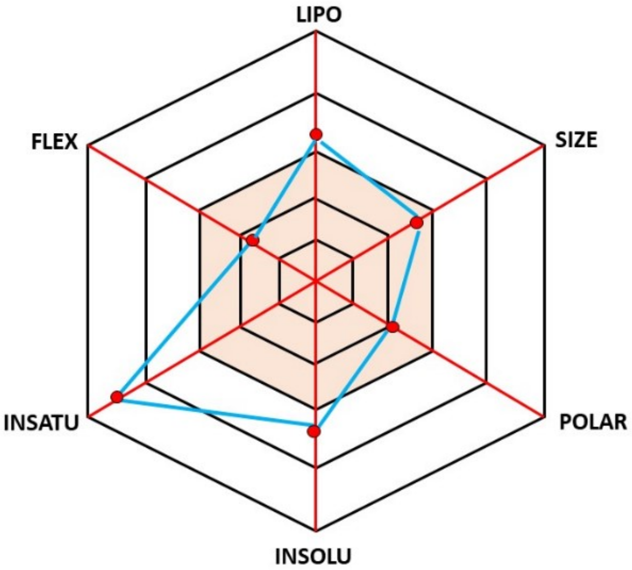

7a

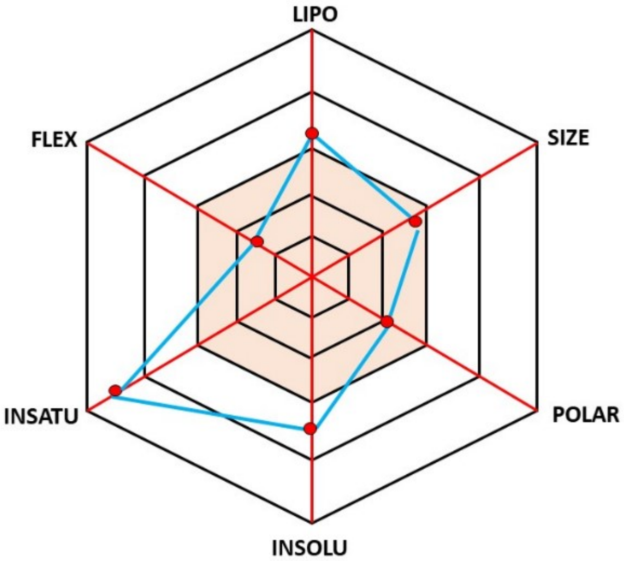

7b

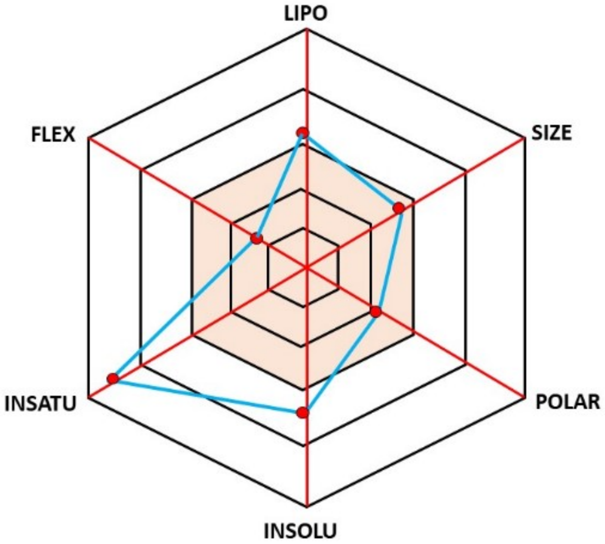

7e

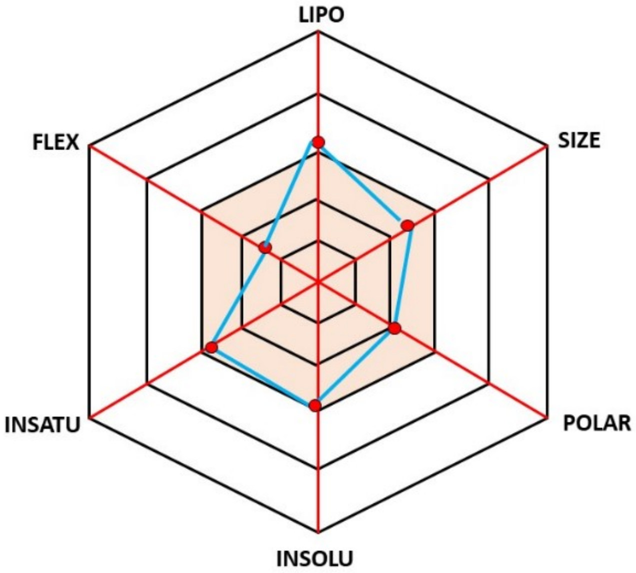

7f

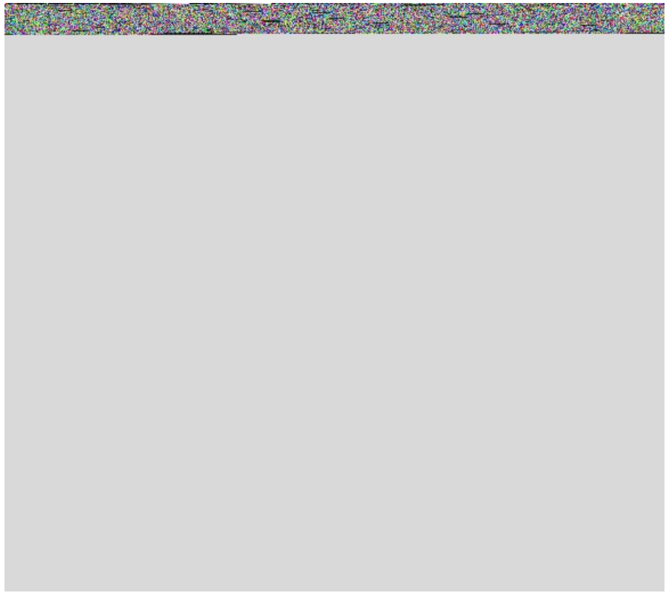

7h

4. HPLC Method Information

Compound-7a

HPLC Method:

Flow	1.0 ml/min
Column name & Dimension	LUNA OMEGA C18 150X4.6mm, 5.0 µm
Column Temperature	40°C
Mobile Phase(A)	0.1% HCOOH in Water
Mobile Phase(B)	Acetonitrile
Gradient :(Time/ % of (Mobile phase B)	0/30,1/70,6/100,8/100,10/30,12/30
Instrument Make & model	Waters ARC with Empower -3 Software
Run time	12 min

Fig S29: HPLC data for 2-(1-(2,4-dichlorobenzyl)-1H-indazol-3-yl)-5-phenyl-1,3,4-oxadiazole **(7a)**

Compound-7b

HPLC Method:

Flow	0.8 ml/min

Flow : 1.0ml/min
Mobile phase : (A) 0.1% FORMIC ACID IN WATER, (B) ACN
Column : LUNA OMEGA C18 150X4.6mm, 5.0 μm
Gradient : T/%B 0/30,1/70,6/100,8/100,10/30,12/30
Column Temp : 40°C 29/11/2024

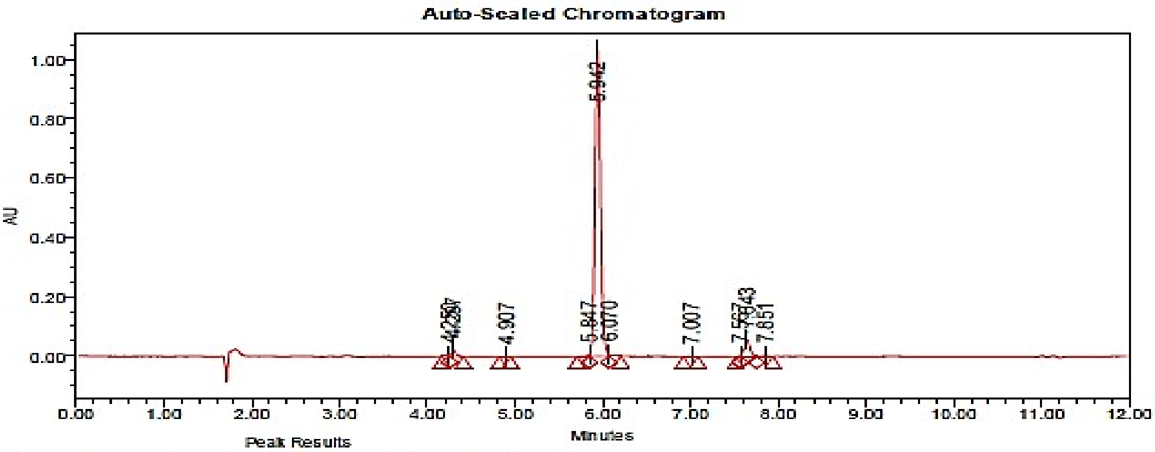

Peak Results

	Name	RT	Area	% Area	Height	Amount	Units
1		4.250	8076	0.17	3002		
2		4.297	70533	1.49	21178		
3		4.907	3585	0.08	1225		

Peak Results

	Name	RT	Area	% Area	Height	Amount	Units
4		5.847	8832	0.19	6152		
5		5.942	4367906	92.19	1034128		
6		6.070	16715	0.35	6409		
7		7.007	6981	0.15	1711		
8		7.567	1531	0.03	1906		
9		7.643	242136	5.11	54408		
10		7.851	11736	0.25	1995		

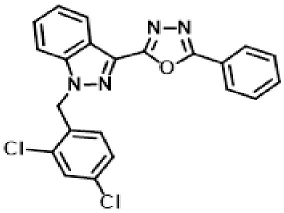

Column name & Dimension	WATERS XBRIDGE C18 ,3.5µm ,4.6*50 mm
Column Temperature	40°C
Mobile Phase(A)	0.1% HCOOH in Water
Mobile Phase(B)	Acetonitrile
Gradient :(Time/ % of (Mobile phase B)	0/30,1/70,6/100,8/100,10/30,12/30
Instrument Make & model	Waters ARC with Empower -3 Software
Run time	12 min

Fig 30: HPLC data 2-(1-(2,4-dichlorobenzyl)-1H-indazol-3-yl)-5-(p-tolyl)-1,3,4-oxadiazole **(7b)**

Analysis Method : E:\DATA\1\METHODS\AGILENT_010_X-BRIDGE C18_10MIN.M
Moblie Phase : 0.1% HCOOH in Water (A) / ACN (B)
Gradient Time Vs % B : 0.0/2,1/2,2.5/50,5/98,8/98,8.1/2,10/2,
Column :WATERS XBRIDGE C18 ,3.5µm ,4.6*50 mm, Flow :0.8ml /min, Temp->40°C MSD1:-Positive Mode, MSD2:-Negative Mode

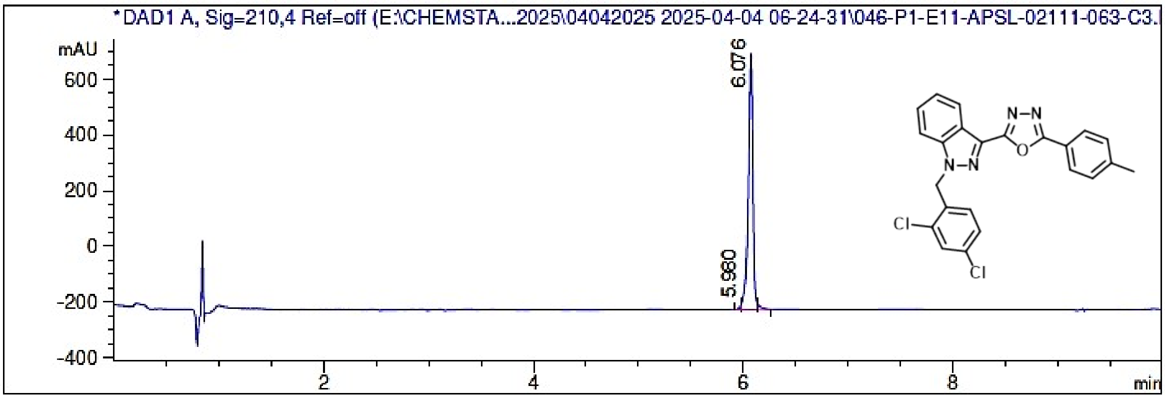

Signal 1: DAD1 A, Sig=210,4 Ref=off

Peak #	RT [min]	Width [min]	Height	Area	Area %	Name
1	5.980	0.019	15.297	17.346	0.620	
2	6.076	0.050	919.622	2748.474	98.281	
3	6.144	0.031	16.717	30.718	1.098	
				932.179	Average	

Compound-7c

HPLC Method:

Flow	1 ml/min
Column name & Dimension	WATERS X-SELECT CSH C18 150X4.6mm, 3.5.0 µm
Column Temperature	40°C
Mobile Phase(A)	0.1% HCOOH in Water
Mobile Phase(B)	Acetonitrile
Gradient :(Time/ % of (Mobile phase B)	0/30,1/70,6/100,8/100,10/30,12/30
Instrument Make & model	Waters ARC with Empower -3 Software
Run time	12 min

Flow : 1.0ml/min
Mobile phase : (A) 0.1% FORMIC ACID IN WATER, (B) ACN
Column : WATERS X-SELECT CSH C18 150X4.6mm, 3.5.0 µm
Gradient : T/%B 0/30,1/70,6/100,8/100,10/30,12/30
Column Temp : 40°C 25/01/2025

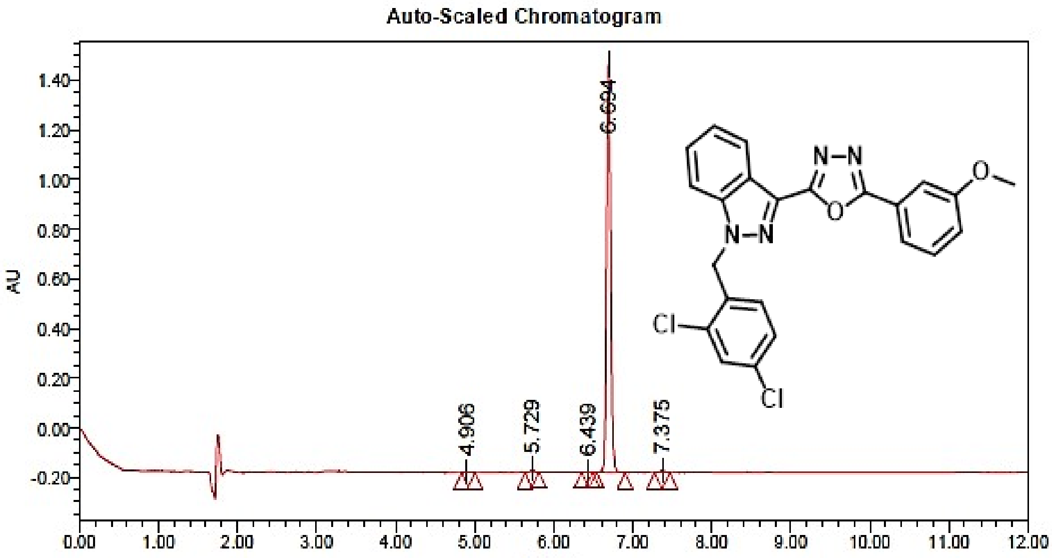

Peak Results

	Name	RT	Area	% Area	Height	Amount	Units
1		4.906	7983	0.14	2203		
2		5.729	43965	0.77	13512		
3		6.439	6144	0.11	2015		
4		6.694	5646520	98.33	1648462		
5		7.375	37985	0.66	11463		

Fig 31: HPLC data for 2-(1-(2,4-dichlorobenzyl)-1H-indazol-3-yl)-5-(3-methoxyphenyl)-1,3,4-oxadiazole **(7c)**

Compound-7d

HPLC Method:

Flow	1 ml/min
Column name & Dimension	WATERS X-SELECT CSH C18 150X4.6mm, 3.5.0 µm
Column Temperature	40°C
Mobile Phase(A)	0.1% HCOOH in Water
Mobile Phase(B)	Acetonitrile
Gradient :(Time/ % of (Mobile phase B)	0/30,1/70,6/100,8/100,10/30,12/30
Instrument Make & model	Waters ARC with Empower -3 Software
Run time	11 min

Fig 32. HPLC data for 2-(4-chlorophenyl)-5-(1-(2,4-dichlorobenzyl)-1H-indazol-3-yl)-1,3,4-oxadiazole **(7d)**

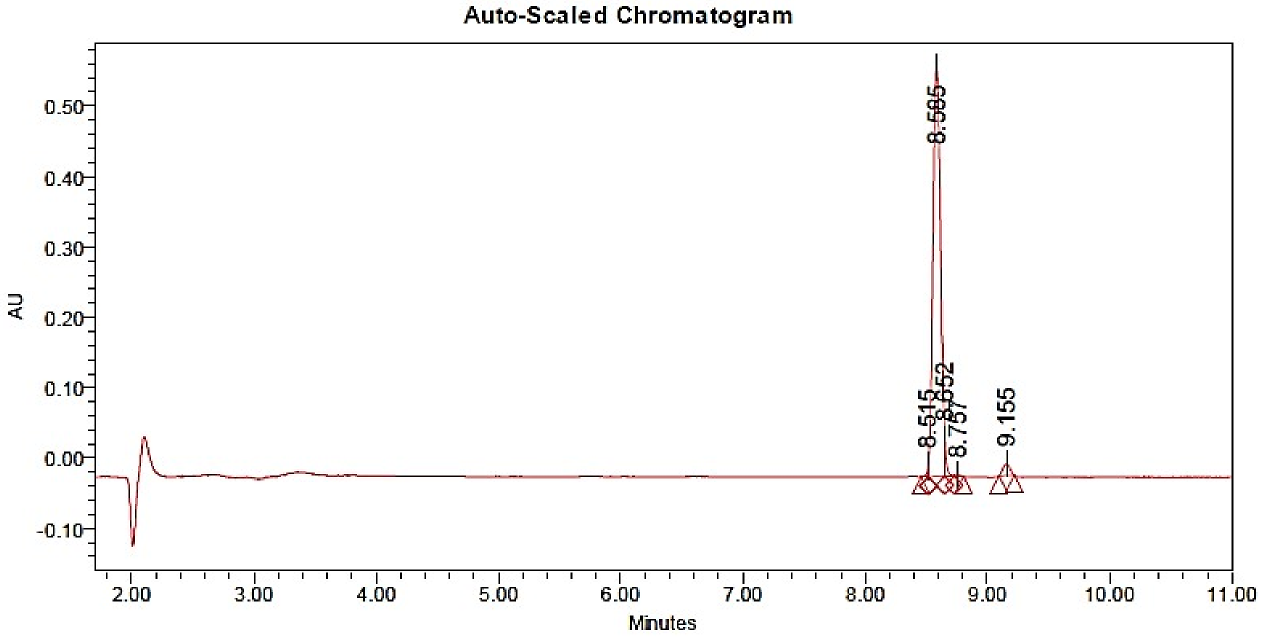

Peak Results

	Name	RT	Area	% Area	Height	Amount	Units
1		8.515	9159	0.35	15641		

Peak Results

	Name	RT	Area	% Area	Height	Amount	Units
2		8.585	2451640	94.89	582115		
3		8.652	48930	1.89	53168		
4		8.757	5385	0.21	1687		
5		9.155	68666	2.66	16827		

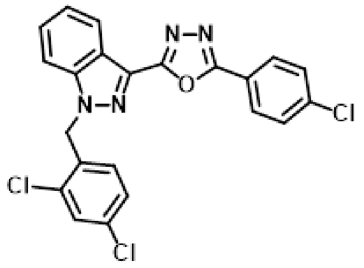

Compound-7e

HPLC Method:

Flow	1 ml/min
Column name & Dimension	LUNA OMEGA C18 150X4.6mm, 5.0 µm
Column Temperature	40°C
Mobile Phase(A)	0.1% HCOOH in Water
Mobile Phase(B)	Acetonitrile
Gradient :(Time/ % of (Mobile phase B)	0/30,1/70,6/100,8/100,10/30,12/30
Instrument Make & model	Waters ARC with Empower -3 Software
Run time	12 min

Flow : 1.0ml/min
Mobile phase : (A) 0.1% FORMIC ACID IN WATER, (B) ACN
Column : LUNA OMEGA C18 150X4.6mm, 5.0 µm
Gradient : T/%B 0/30,1/70,6/100,8/100,10/30,12/30
Column Temp : 40°C 04/04/2025

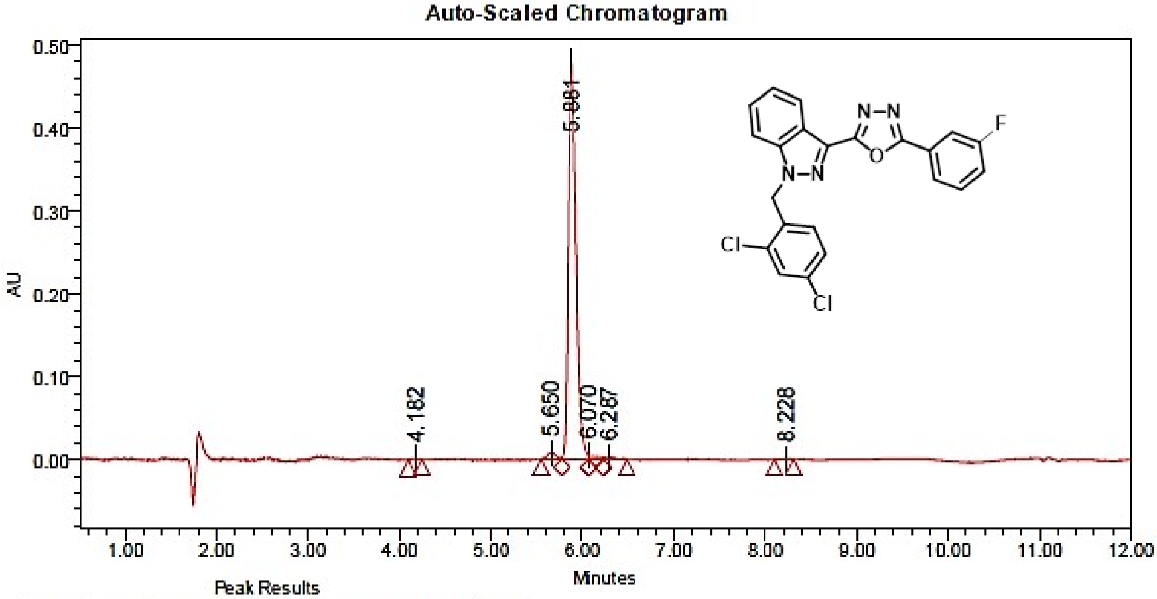

Peak Results

	Name	RT	Area	% Area	Height	Amount	Units
1		4.182	8394	0.27	1623		
2		5.650	50040	1.62	7776		
3		5.881	2959574	96.09	480167		

Fig 33. HPLC data for 2-(1-(2,4-dichlorobenzyl)-1H-indazol-3-yl)-5-(3-fluorophenyl)-1,3,4-oxadiazole **(7e)**

Compound-7f

HPLC Method:

Flow	1 ml/min
Column name & Dimension	LUNA OMEGA C18 150X4.6mm, 5.0 µm
Column Temperature	40°C
Mobile Phase(A)	0.1% HCOOH in Water
Mobile Phase(B)	Acetonitrile
Gradient :(Time/ % of (Mobile phase B)	0/30,1/70,6/100,8/100,10/30,12/30
Instrument Make & model	Waters ARC with Empower -3 Software
Run time	12 min

Fig 34. HPLC data for 2-(1-(2,4-dichlorobenzyl)-1H-indazol-3-yl)-5-(4-fluorophenyl)-1,3,4-oxadiazole **(7f)**

Compound-7g

HPLC Method:

Flow : 1.0ml/min
Mobile phase : (A) 0.1% FORMIC ACID IN WATER, (B) ACN
Column : LUNA OMEGA C18 150X4.6mm, 5.0 µm
Gradient : T/%B 0/30,1/70,6/100,8/100,10/30,12/30
Column Temp : 40°C 04/04/2025

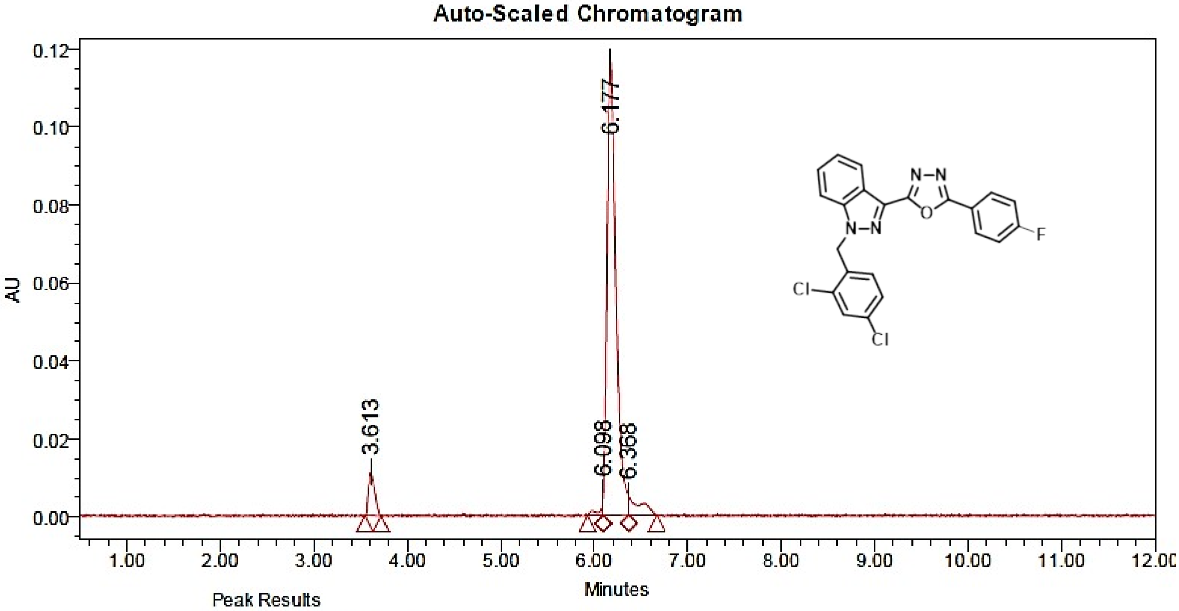

Peak Results

	Name	RT	Area	% Area	Height	Amount	Units
1		3.613	49859	6.04	11065		
2		6.098	9779	1.18	5599		
3		6.177	724603	87.76	116275		

Flow	1 ml/min
Column name & Dimension	WATERS X-SELECT CSH C18 150X4.6mm, 3.5µm
Column Temperature	40°C
Mobile Phase(A)	0.1% HCOOH in Water
Mobile Phase(B)	Acetonitrile
Gradient :(Time/ % of (Mobile phase B)	0/30,1/70,6/100,8/100,10/30,12/30
Instrument Make & model	Waters ARC with Empower -3 Software
Run time	12 min

Fig 35. HPLC data for 2-(1-(2,4-dichlorobenzyl)-1H-indazol-3-yl)-5-(thiophen-2-yl)-1,3,4-oxadiazole **(7g)**

Flow : 1.0ml/min
Mobile phase : (A) 0.1% FORMIC ACID IN WATER, (B) ACN
Column : WATERS X-SELECT CSH C18 150X4.6mm, 3.5µm
Gradient : T/%B 0/30,1/70,6/100,8/100,10/30,12/30
Column Temp : 40°C 18/02/2025

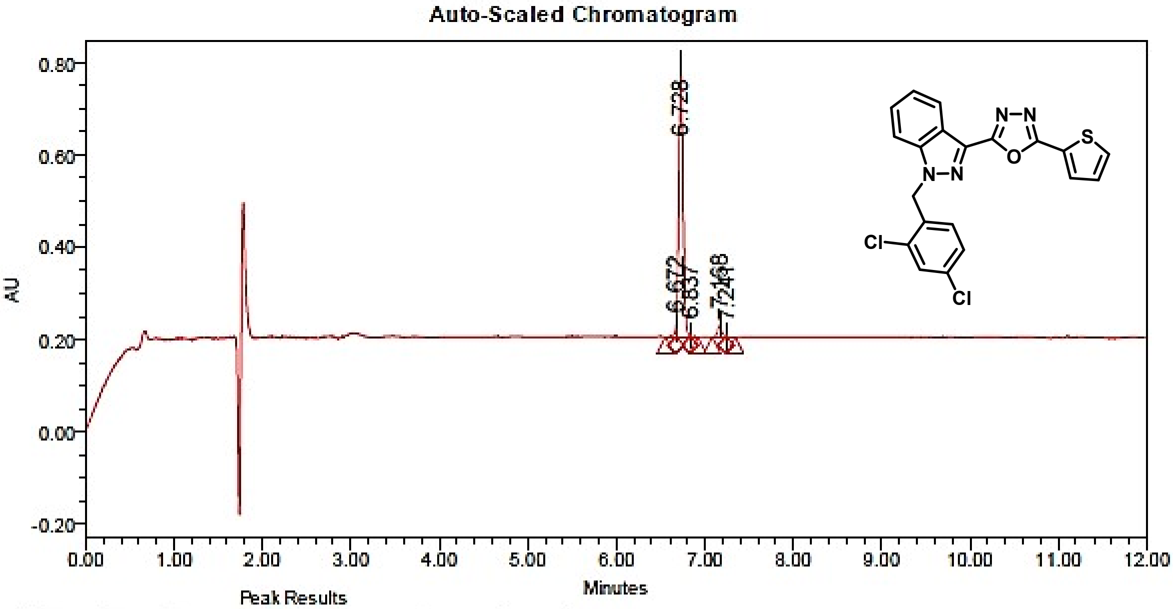

Peak Results

	Name	RT	Area	% Area	Height	Amount	Units
1		6.672	20813	0.99	18004		
2		6.728	1989456	94.96	592939		
3		6.837	2428	0.12	1882		

Compound-7h

HPLC Method:

Flow	1 ml/min
Column name & Dimension	WATERS X-SELECT CSH C18 150X4.6mm, 3.5.0 µm
Column Temperature	40°C
Mobile Phase(A)	0.1% HCOOH in Water
Mobile Phase(B)	Acetonitrile
Gradient :(Time/ % of (Mobile phase B)	0/30,1/70,6/100,8/100,10/30,12/30
Instrument Make & model	Waters ARC with Empower -3 Software
Run time	12 min

Flow : 1.0ml/min
Mobile phase : (A) 0.1% FORMIC ACID IN WATER, (B) ACN
Column : WATERS X-SELECT CSH C18 150X4.6mm, 3.5.0 µm
Gradient : T/%B 0/30,1/70,6/100,8/100,10/30,12/30
Column Temp : 40°C 25/01/2025

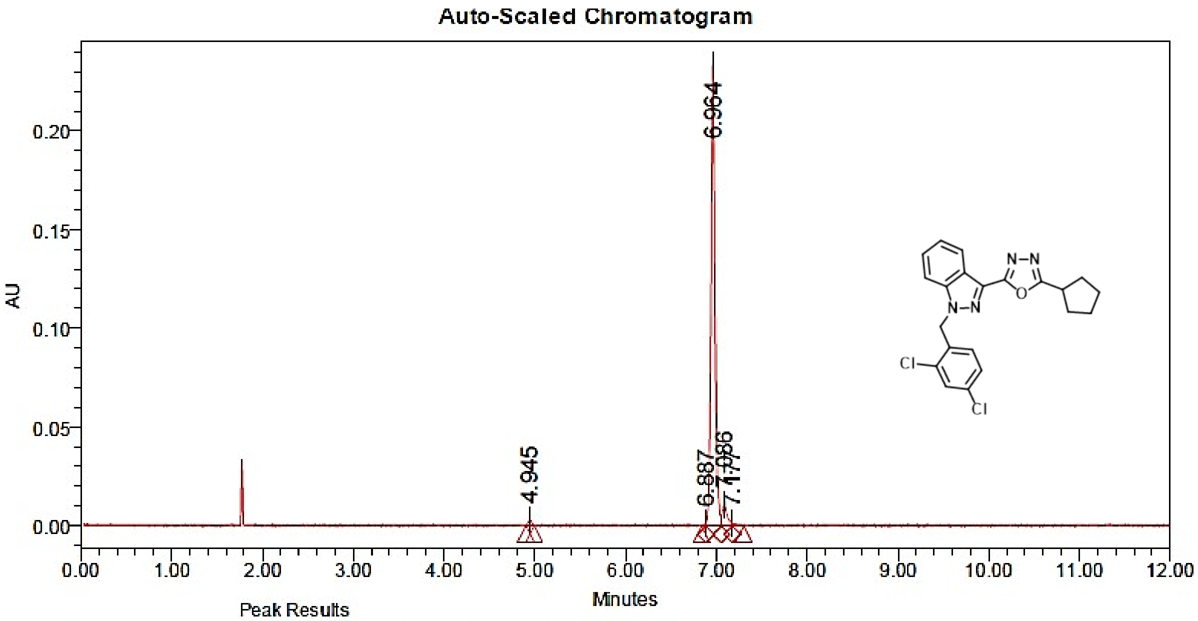

Peak Results

	Name	RT	Area	% Area	Height	Amount	Units
1		4.945	7420	1.01	2749		
2		6.887	1172	0.16	1201		
3		6.964	684352	92.73	233557		

Fig 36. HPLC data for 2-cyclopentyl-5-(1-(2,4-dichlorobenzyl)-1H-indazol-3-yl)-1,3,4-oxadiazole **(7h)**

5. DFT Studies

All computational studies were conducted using the Gaussian16W software, and the results were compared with experimental results. Density functional theory (DFT) was performed using the basis set B3LYP/6-31G++ to optimise the molecule in the ground state.

3.3.3.1 Frontier molecular orbital (FMO) analysis

To study the stability, reactivity, and physicochemical features of the newly synthesised derivatives (**7c**, **7d** and **7g**), their frontier molecular orbitals (FMOs) were computationally analysed. The DFT calculations were done at the B3LYP/6-311G++ level of theory in the gaseous phase to calculate the energies of the highest occupied molecular orbital (HOMO) and lowest unoccupied molecular orbital (LUMO), the orbital energy gap (Egap), and the dipole moment. The HOMO-LUMO energy gap is an important factor in photochemical, electrical, and chemical reactions. In general, the HOMO-LUMO energy gap is a significant factor in the design and optimization of OLED materials, especially for producing blue light emissions in these technologically relevant devices. The HOMO, LUMO, and optimal molecular geometry of the synthesized compounds are displayed in detail in **Table 1**. To gain insight into the chemical reactivity and stability of molecules, global reactivity parameters were calculated using the following equations **(1) to (6)**.

$$\eta = \frac{(IP - EA)}{2} \qquad (1)$$

$$\chi = \frac{(IP + EA)}{2} \qquad (2)$$

$$V = -\frac{(IP + EA)}{2} \qquad (3)$$

$$\mu = -\chi \qquad (4)$$

$$S = \frac{1}{\eta} \qquad (5)$$

$$\omega = \frac{\mu^2}{2\eta} \qquad (6)$$

Table 1. Optimised molecular geometry and HOMO, LUMO of the synthesized molecule

Comp	Optimized	HOMO	LUMO
7c	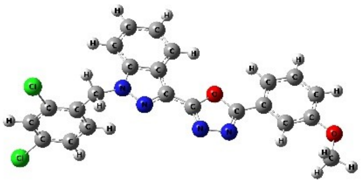	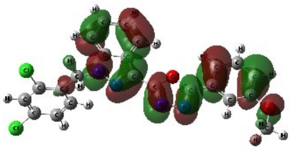	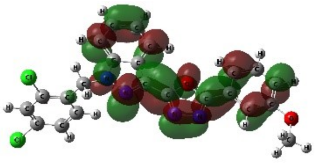
7d	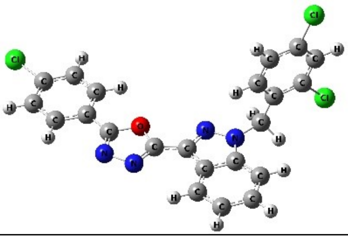	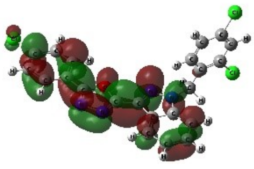	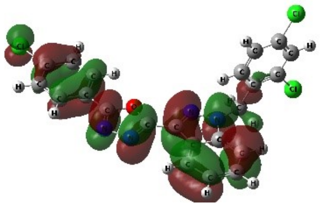
7g	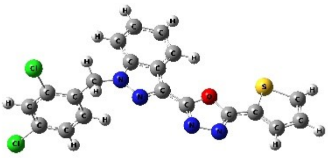	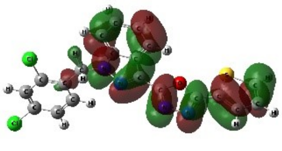	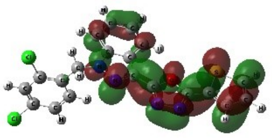

The GCRD parameters can be found in **Table 2,** and the energy gap between HOMO and LUMO corresponds to the chemical stability and reactivity of the molecule. Typically, lower

gaps possess higher reactivity but lower stability, whereas more significant gaps correspond to higher strength and lower reactivity. The LUMO, which accepts electrons during molecular interactions, is related to the electron affinity (EA = $-E_{LUMO}$), while the HOMO, representing electron donors, is related to the ionization potential (IP = $-E_{HOMO}$). A molecule with a large HOMO-LUMO energy gap is kinetically stable and has low chemical reactivity because adding an electron to the high-lying LUMO or removing electrons from the low-lying HOMO is energetically unfavourable. The energy gaps of **(7c)** (4.545 eV) and (7d) (4.543 eV) are approximately equal, representing comparable stability and low chemical reactivity. Compound **(7g)** shows a smaller gap (4.364 eV), suggesting relatively lower stability and higher chemical reactivity. In contrast, compounds with a short HOMO-LUMO energy gap are less stable and more chemically reactive. Consequently, the compound is harder and more stable (less reactive) according to the GCRD measure [1-4]. This could be due to the substitution of the indazole moiety, which produces the greatest energy gap by being non-planarly attached to oxadiazole. These substituents make the molecules more reactive.

Table 2. The Global Chemical Reactivity Descriptors (GCRD)

**Compounds**	**7c**	**7d**	**7g**
E_{HOMO} (eV)	-7.079	-7.252	-7.056
E_{LUMO} (eV)	-2.534	-2.709	-2.692
Ionization potential IP (eV)	7.079	7.252	7.056
Electron affinity EA (eV)	2.534	2.709	2.692
Energy gap Eg (eV)	4.545	4.543	4.364
Electronegativity χ (eV)	4.807	4.981	4.874

Chemical potential V (eV)	-4.8065	-4.981	-4.874
Chemical hardness η (eV)	2.273	2.272	2.182
Softness S(eV)	0.440	0.440	0.458
Chemical potential μ (eV)	-4.807	-4.981	-4.874
Electrophilicity index ψ (eV)	5.083	5.460	5.444

The molecular electron cloud's deformation is significantly influenced by minor perturbations, showcasing the inherent resistance that influences the hardness observed in chemical processes. Unlike soft molecules, which are larger and more polarizable, hard molecules are comparatively smaller and have limited polarizability. Since hardness and softness have an inverse relationship, hardness is essential for communicating a molecule's stability and intermolecular reactivity. The ability of an electrophile to acquire an extra electrical charge is closely related to electrophilicity phenomena. It is a chemical characteristic that shows the molecule's resistance to electron exchange with its environment, thereby exposing its hardness and chemical potential-two factors that are essential to comprehending stability. The electrophilicity index is a crucial property used to assess a compound's reactivity and selectivity, particularly regarding toxicity. The energy reduction brought about by the maximum electron flow between a donor and an acceptor is measured by this index, which is derived from the energy values of the LUMO and HOMO. A strong nucleophile with a lower ω value is more likely to donate electrons, whereas a strong electrophile with a higher ω value is more likely to accept electrons. Thus, robust electrophiles are organic molecules with an electrophilicity scale (ω) larger than 1.5 eV. Molecules below 0.8 eV are classified as weaker electrophiles, while those between 0.8 eV and 1.5 eV are regarded as moderate electrophiles

[5-8]. Our extensive investigation indicates that the synthesized compounds in this work have strong electrophilic properties, as evidenced by their increased ω values.

The chemical potential (μ) determines how easily electrons can leave a stable system. It is worth noting that a complex with a negative chemical potential value is considered stable and does not spontaneously degrade into its essential elements. As a result, the synthetic molecules, which have negative chemical potentials, do not undergo such disintegration. Furthermore, the HOMO-LUMO gap, a measure of chemical hardness, strongly suggests that these molecules have low polarizability and can withstand the deformation of the electron cloud inside the chemical system, even under significant shocks. Electrostatic potential maps are critical for understanding the electrical properties of blue OLEDs. They visibly represent electron density distribution around a molecule, providing insights into charge transport, molecular design, and intermolecular interactions, all of which influence OLED performance. Understanding charge distribution aids in designing molecules with favorable electrical characteristics, resulting in effective transport within the device's organic layers. The maps also show the highest occupied HOMO and LUMO levels, which help optimize the HOMO-LUMO energy gap for blue light emission. The energy gap between HOMO and LUMO indicates the molecule's chemical stability and reactivity. Typically, lower gaps possess higher reactivity but lower stability, whereas more significant gaps correspond to higher strength and lower reactivity. Such molecules, having less electrophilicity, are generally less reactive, very stable, and poor electron acceptors. [4,9].

3.3.3.2 Molecular electrostatic potentials (MEP) analysis

The MEP analysis is based on electron density and is beneficial to study the molecular properties such as hydrogen bonding, electrophilic, and nucleophilic attacks on the molecular site [5]. This study is helpful to find out the size, shape, and charge regions on the molecule in terms of the colour code [10]. The red colour demonstrates the highest negative charge, and

the blue colour shows the highest positive charge in the molecule [5]. The MEP surface is plotted on the optimised molecule and shown in **Figure S1**. The oxygen atom (O-1) of the **(7c)**, **(7d)** and **(7g)** is having a deep red colour, and it is responsible for the highest electrophilic attack. The nitrogen atom (N-3) and (N-4) of the **(7c)**, **(7d)** and **(7g)**, respectively, are the second-most responsible atoms for the electrophilic attack. The hydrogen atoms (H-35, H-42, H-45) for (7c), (H-34, H-35, H-37, H-38, H-39, H-40, H-41 and H-42) for **(7d)** and (H-30, H-31, H-34, H-35, and H-38) for **(7g)**, respectively, have a deep dark blue region. Hence, these atoms are considered the most responsible for the nucleophilic attack. The high proportion of the light blue colour probably represents the neutral region between the two extremes, red and blue. **Table 3** shows the Mulliken Charges and ground-state dipole moment; arrow marks show the direction of the dipole moment, which confirms the nucleophilic attack and electrophilic attack.

Table 3. Mulliken Charges and Ground-state dipole moment of molecules (**7c**, **7d**, and **7g**). [Arrow marks show the direction of the dipole moment]

**Comp**	**Mulliken charges**	**Direction of Dipole moment**
7c	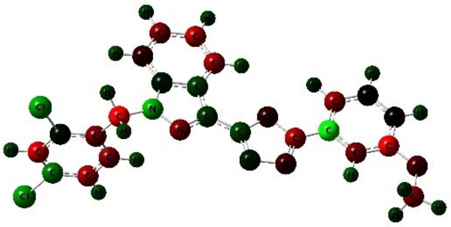	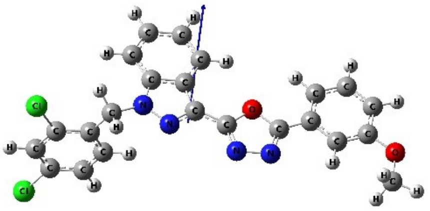
7d	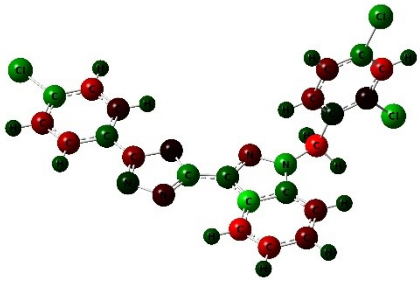	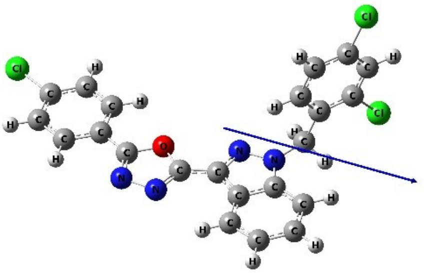

7g

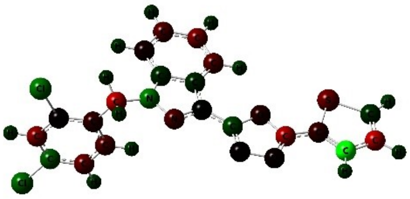

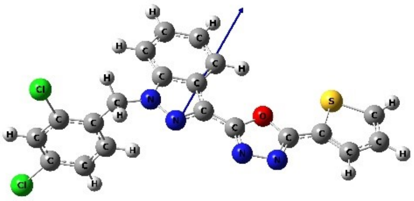

(7c)

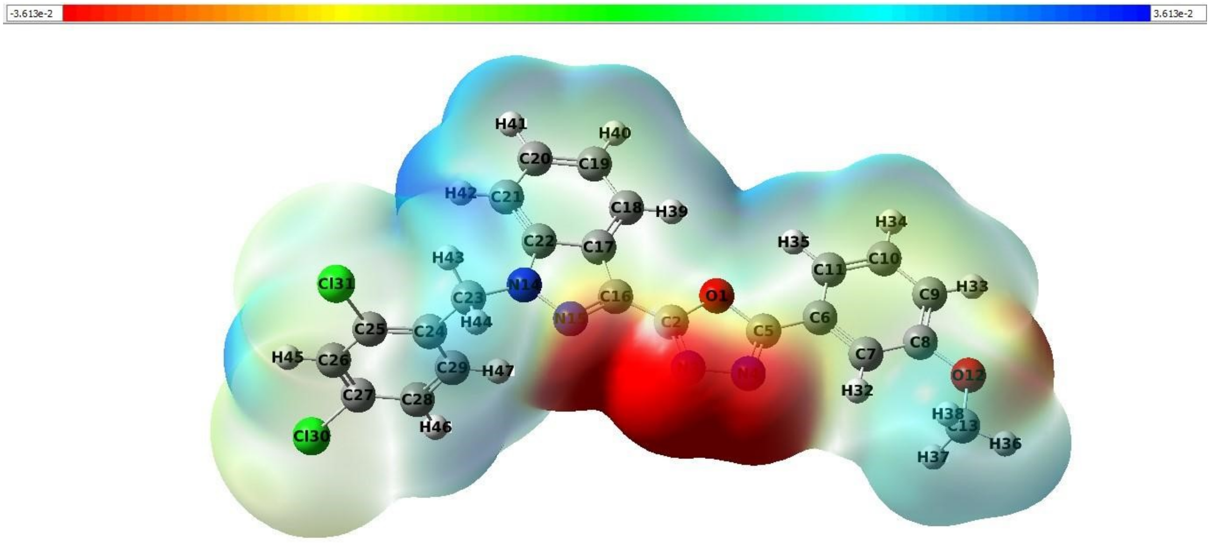
-3.613e-2
3.613e-2
H41
H40
C20
C19
H42
C21
C18
H39
C22
C17
H34
H35
C11
C10
H43
Cl31
N14
C23
C16
O1
C9
H33
N1
C2
C5
C6
C25
C24
H44
H45
C26
C29
H47
C7
C8
C27
C28
H32
O12
H38
C13
H36
Cl30
H46
H37

(7d)

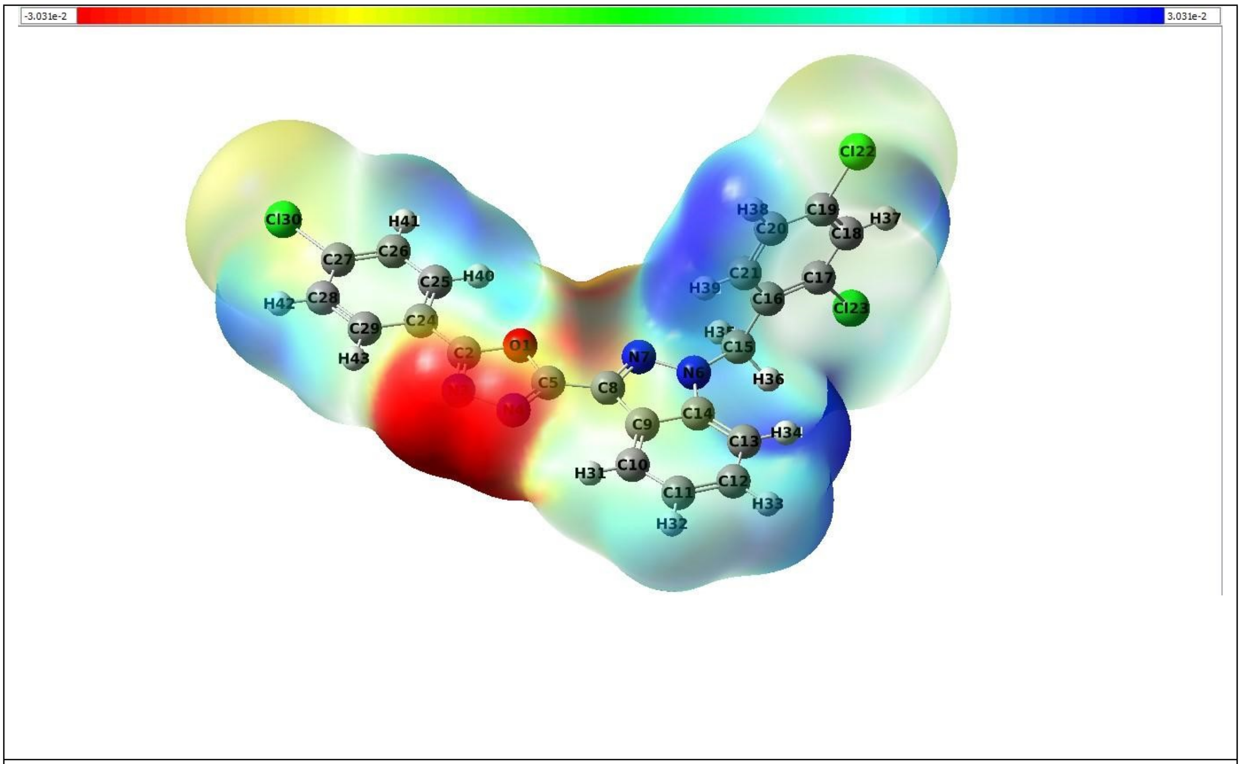

(7g)

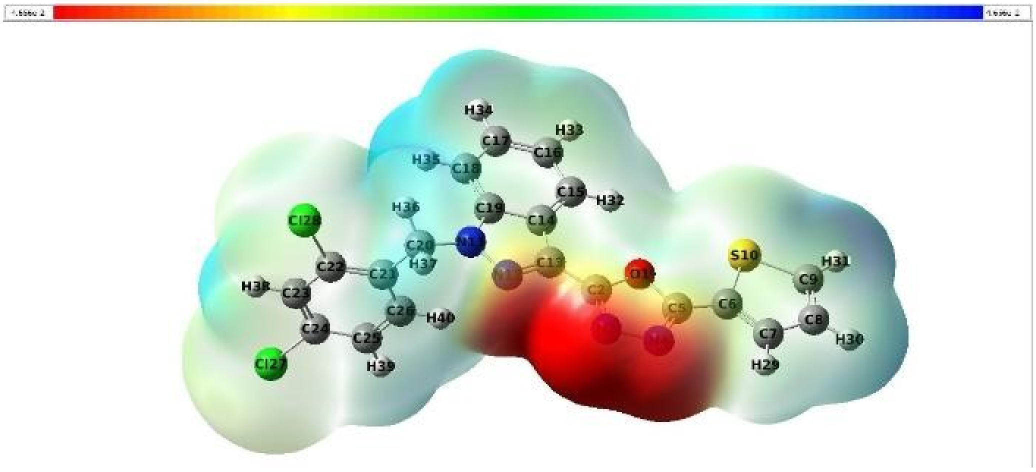

Fig S1. Electrostatic potential map of molecules **7c**, **7d**, and **7g**

6. Video of molecular simulation study

a) For compound **7a**

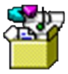

7a.mp4

b) For compound **7c**

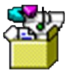

7c.mp4

c) For compound **7d**

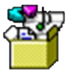

7d.mp4

d) For compound **7g**

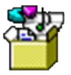

7g.mp4

References

[1] M.A. Doddagaddavalli, M. Madar, J. Seetharamappa, Fluorescent schiff bases with phenothiazine and coumarin moieties: synthesis, characterization, photophysical, electrochemical, computational and biological studies, J Fluoresc 35 (2024) 3003–3017. https://doi.org/10.1007/s10895-024-03705-w.

[2] A. Irfan, M. Imran, N. Khalid, M. Ahmad, A.R. Chaudhry, M. Hussien, S.Y. DaifAllah, A.G. Al-Sehemi, H.D. Almalki, M.A. Qayyum, A. Saral, A. Manikandan, S. Muthu, Molecular level interaction, HOMO-LUMO, MEP, UV–Vis, Hirshfeld, topological analysis, and *in-vitro* of isoflavones from Eremostachys Vicaryi Benth. Ex Hook. f., J Mol Struct 1303 (2024) 137581. https://doi.org/10.1016/j.molstruc.2024.137581.

[3] V. Marrakkur, B.K. Sarojini, M. Madar, S.-H. Dhanur, T.M. Sridhara, C.-G.-D. Raj, H. Pavankumar, H.-M. Savanur, V.-R. Shetty, L. Naik, Computational and experimental investigation on biological and photophysical properties of high yielded novel aryl-substituted

pyrazolone analogue, J Mol Struct 1276 (2023) 134790. https://doi.org/10.1016/j.molstruc.2022.134790.

[4] V. Hegde, R. Bhat, V. Sharma, V. Adimule, R. Keri, P. Kumar, S. D V, G. V. Muddapur, S. Nandi, Structural characterization of photoluminescent, light-driven fluorescence and phosphorescence decay properties of new azobenzene dyes containing alkoxy side chain and their DFT studies, J Mol Struct 1336 (2025) 142092. https://doi.org/10.1016/j.molstruc.2025.142092.

[5] R. Palled, V. Srinivasan, B.A. Al-Asbahi, L. Naik, A. S G, M.P. Eelager, A. Sidarai, M. Madar, A comprehensive investigation of ethyl 2-(3-methoxybenzyl) acrylate substituted pyrazolone analogue: Synthesis, computational and biological studies, Chemical Physics Impact 8 (2024) 100531. https://doi.org/10.1016/j.chphi.2024.100531.

[6] R.G. Parr, Density functional theory of atoms and molecules, in: horizons of quantum chemistry, Springer Netherlands, Dordrecht, (1980) 5–15. https://doi.org/10.1007/978-94-009-9027-2_2.

[7] V. Choudhary, A. Bhatt, D. Dash, N. Sharma, DFT calculations on molecular structures, HOMO–LUMO study, reactivity descriptors and spectral analyses of newly synthesized diorganotin(IV) 2-chloridophenylacetohydroxamate complexes, J Comput Chem 40 (2019) 2354–2363. https://doi.org/10.1002/jcc.26012.

[8] L. Domingo, M. Ríos-Gutiérrez, P. Pérez, Applications of the conceptual density functional theory indices to organic chemistry reactivity, Molecules 21 (2016) 748. https://doi.org/10.3390/molecules21060748.

[9] N.B. Gummagol, D.A. Yaraguppi, S.B. Patil, P.S. Patil, N.R. Patil, N.H. Ayachit, Exploring the anticancer potential of novel chalcone derivatives: Synthesis, characterization, computational analysis, and biological evaluation against breast cancer, J Mol Struct 1320 (2025) 139586. https://doi.org/10.1016/j.molstruc.2024.139586.

[10] J.G. Polashi, B.B.A. Mulla, G. V. Muddapur, N. Al-Zaqri, S. H P, V. Nayak, A.H. Sidarai, Photophysical and quantum chemical insights into coumarin derivatives for optoelectronics applications, J Fluoresc (2025). https://doi.org/10.1007/s10895-025-04438-0.
